# Transcriptomic responses in the nervous system and correlated behavioural changes of a cephalopod exposed to ocean acidification

**DOI:** 10.1186/s12864-024-10542-5

**Published:** 2024-06-25

**Authors:** Jodi T. Thomas, Roger Huerlimann, Celia Schunter, Sue-Ann Watson, Philip L. Munday, Timothy Ravasi

**Affiliations:** 1 Australian Research Council Centre of Excellence for Coral Reef Studies, James Cook University, Townsville, QLD 4811 Australia; 2 Marine Climate Change Unit, Okinawa Institute of Science and Technology Graduate University, Okinawa, Japan; 3 Swire Institute of Marine Science, School of Biological Sciences, The University of Hong Kong, Pok Fu Lam Road, Hong Kong SAR China; 4 College of Science and Engineering, James Cook University, Townsville, QLD 4811 Australia; 5 Biodiversity and Geosciences Program, Queensland Museum Tropics, Queensland Museum, Townsville, QLD 4810 Australia

**Keywords:** Carbon dioxide, Climate change, Gene expression, Behaviour, Neurotransmission, Neuroplasticity, Immune, Oxidative stress

## Abstract

**Background:**

The nervous system is central to coordinating behavioural responses to environmental change, likely including ocean acidification (OA). However, a clear understanding of neurobiological responses to OA is lacking, especially for marine invertebrates.

**Results:**

We evaluated the transcriptomic response of the central nervous system (CNS) and eyes of the two-toned pygmy squid (*Idiosepius pygmaeus*) to OA conditions, using a *de novo* transcriptome assembly created with long read PacBio ISO-sequencing data. We then correlated patterns of gene expression with CO_2_ treatment levels and OA-affected behaviours in the same individuals. OA induced transcriptomic responses within the nervous system related to various different types of neurotransmission, neuroplasticity, immune function and oxidative stress. These molecular changes may contribute to OA-induced behavioural changes, as suggested by correlations among gene expression profiles, CO_2_ treatment and OA-affected behaviours.

**Conclusions:**

This study provides the first molecular insights into the neurobiological effects of OA on a cephalopod and correlates molecular changes with whole animal behavioural responses, helping to bridge the gaps in our knowledge between environmental change and animal responses.

**Supplementary Information:**

The online version contains supplementary material available at 10.1186/s12864-024-10542-5.

## Background

As human-induced environmental changes progress, establishing how animals respond to projected future environmental conditions, and why these responses occur, is critical [1]. A thorough understanding of why biological responses are occurring is especially useful for gaining insight into why some individuals or species are more sensitive to environmental change than others, and improving predictions of how organisms and populations will respond over the timescales at which environmental change is occurring [2]. The nervous system forms the fundamental link between the environment and animal’s responses [3, 4]. Thus, the neurobiological impacts of anthropogenic environmental change are key to understanding how animals will respond as environmental change progresses, yet the role of the nervous system in biological responses to environmental change has been little explored [3].

The uptake of anthropogenic carbon dioxide (CO_2_) by the ocean is causing seawater CO_2_ levels to rise, decreasing seawater pH and altering the concentration of carbonate ions, in a process known as ocean acidification (OA) [5]. These chemical changes can fundamentally affect marine organisms and the ecosystems they inhabit [6]. OA affects a wide variety of physiological processes, life history traits and behaviours of marine invertebrates [7–11]. Invertebrates are vital components of marine ecosystems, comprising over 92% of species in the ocean, are essential to the function of ecosystem processes, and support the livelihoods of human societies across the globe [12, 13]. Animal behaviour influences an individual’s own fitness, complex interactions with other individuals and species, and key ecological processes that shape the structure of marine communities and ecosystems [14]. Consequently, any behavioural effects of elevated CO_2_ on marine invertebrates could potentially have wide-ranging ecological, social and economic consequences.

Despite many studies assessing the behavioural responses of marine invertebrates to OA the link between the environment and behavioural responses, the nervous system, has been largely understudied. The work that has addressed the neurobiological impacts of OA has focused on the functioning of GABA_A_ receptors. The GABA hypothesis was first proposed in fish and suggests acid-base regulatory mechanisms occurring at elevated CO_2_ conditions alter ionic gradients across neuronal membranes, consequently disturbing GABA_A_ receptor function and causing behavioural alterations [15]. A range of research has supported the GABA hypothesis in fish (reviewed in Heuer, Hamilton [16]), and more recently pharmacological studies have also supported the GABA hypothesis in molluscs [17–19], but not a crustacean [20]. However, OA may also have a range of other neurobiological impacts, including altering the function of other ligand-gated ion channels that are similar to the GABA_A_ receptor [18] and affecting synaptic plasticity [21, 22].

Transcriptomics provides a powerful non-targeted, holistic approach to identify functional responses to environmental change. Indeed, transcriptomics has widely been taken up by the OA research community to understand the response of marine animals to elevated CO_2_ [23]. However, there is less research assessing the transcriptomic response of nervous tissue to elevated CO_2_. Recently, studies have examined the transcriptomic response of the fish nervous system to elevated CO_2_ conditions, including in coral reef fishes [24–28], temperate marine fishes [21, 29, 30] and ocean-phase salmon [31]. In marine invertebrates, two transcriptomic studies assessing the whole-body response of pteropod molluscs to elevated CO_2_ identified altered expression of genes involved in nervous system function [32, 33]. However, whole body measurements cannot determine if non-tissue-specific transcripts are responding to elevated CO_2_ in a system-wide manner, or only within specific tissues. Furthermore, due to the heterogeneity and complexity of gene expression, measurements at the whole-body level may mask transcriptomic responses in specific tissues, such as the nervous system.

Here, we investigated the transcriptomic response to OA in the central and peripheral nervous system of a cephalopod, the two-toned pygmy squid (*Idiosepius pygmaeus* [34]), and then correlated the molecular responses with behavioural changes recorded in the same individuals. Cephalopods have complex nervous systems and behaviours rivalling those of fishes [35], making them a useful taxon to investigate the neurobiological impacts of elevated CO_2_. *I. pygmaeus* is a diurnal, tropical squid inhabiting shallow, inshore waters of the Indo-Pacific, including Northern and North-eastern Australia [36, 37]. They are a small, short-lived squid growing to a maximum mantle length of 2 cm [36], and have a lifespan of up to 80 days [38]. *I. pygmaeus* is an ideal species to use as previous research in this species found elevated CO_2_ alters a range of behaviours [18, 39, 40].

In this study, we used RNA from the central nervous system (CNS) and eyes (peripheral sense organ) from squid exposed to current-day (~ 400 µatm) or elevated (~ 1,000 µatm) CO_2_ levels for 7 days in a previous study by Thomas, Spady [18]. In these squid, elevated CO_2_ exposure increased activity levels as well as visually-guided, conspecific-directed attraction and aggression [18]. Here, we created a *de novo* transcriptome assembly, providing a reference which we used to determine the transcriptomic response of the squid CNS and eyes to elevated CO_2_. We used the eyes because cephalopods, including squid, are highly visual animals with many visually-guided behaviours [41–43]. Furthermore, we have shown elevated CO_2_-induced disturbances of visually-guided behaviour in the same squid used in this study [18]. As we had transcriptomic and behavioural data from the same individual squid, we also correlated patterns of gene expression with CO_2_ treatment levels and OA-affected behaviours to determine key genes and processes in the cephalopod CNS and eyes potentially contributing to OA-induced behavioural changes. The results from this study help us understand, at a molecular level, the neurobiological impacts of ocean acidification in a marine invertebrate with a complex nervous system.

## Methods

### Animal collection and experimental setup

**Fig. 1:**
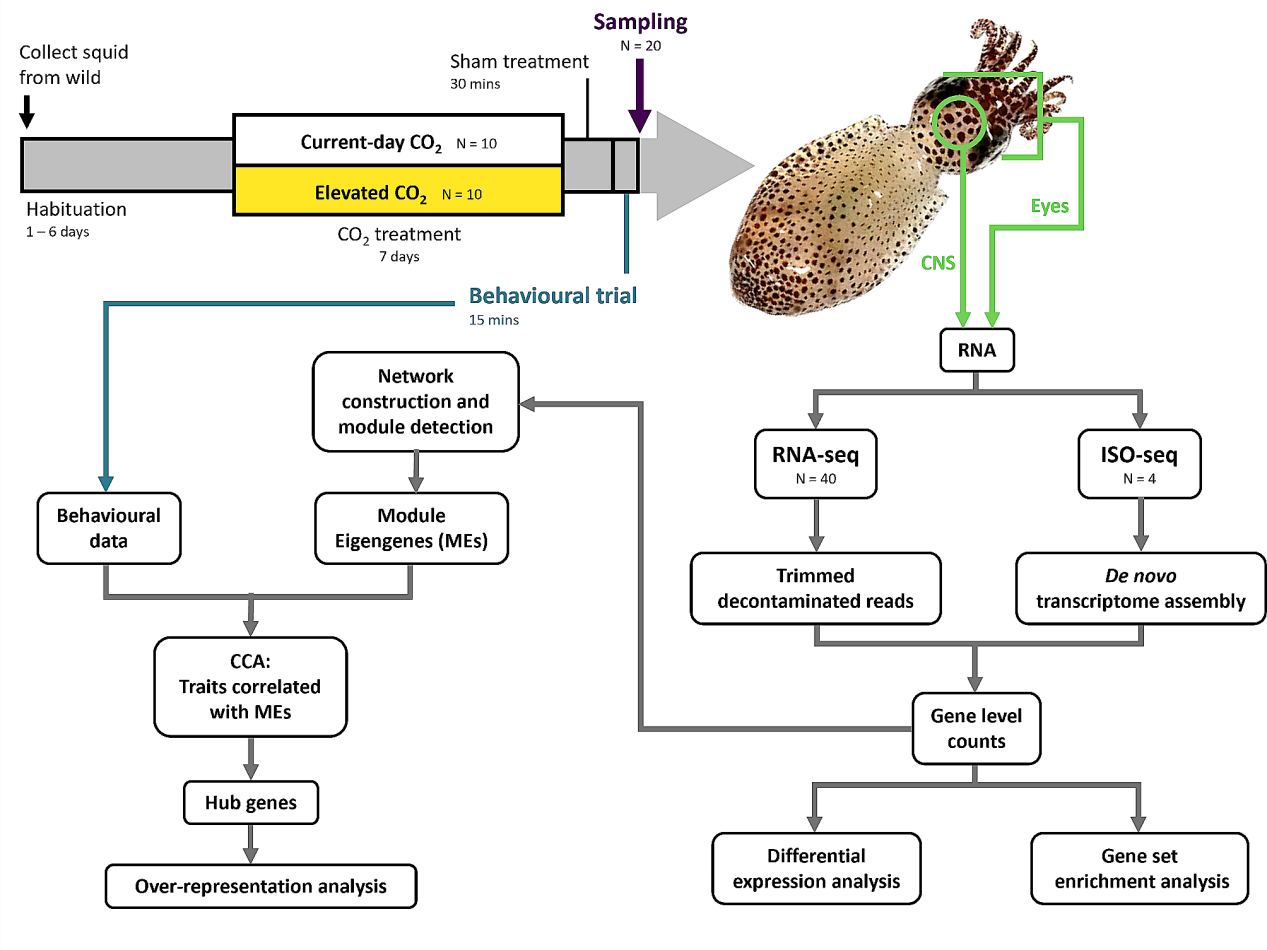
Experimental design overview. CCA = canonical correlation analysis. *Idiosepius pygmaeus* photograph by Jodi Thomas

The squid tissues and behavioural data used in this study came from a previous experiment. Specifically, we used sham-treated squid from the picrotoxin experiment in Thomas, Spady [18] (Fig. 1). As described in Thomas, Spady [18], male two-toned pygmy squid (*Idiosepius pygmaeus*) were collected from the wild and acclimated in groups at current-day seawater conditions for 1–6 days before transferral to individual treatment tanks set at either current-day (~ 400 µatm) or elevated (~ 1,000 µatm) CO_2_ levels, consistent with CO_2_ levels projected for 2100 following the representative concentration pathway RCP8.5 scenario [5]. Experiments were carried out in four interconnected 8,000 L recirculating seawater systems; two untreated seawater systems were used for current-day CO_2_ treatments, and two seawater systems were dosed with CO_2_ using a custom-built pH control system for elevated CO_2_ treatments. The CO_2_ conditions achieved were current-day CO_2_: 407 ± 58 µatm *p*CO_2_, pH_T_ = 8.09 ± 0.10 and elevated CO_2_: 1,071 ± 71 µatm *p*CO_2_, pH_T_ = 7.73 ± 0.03 (mean ± SD). Refer to Thomas, Spady [18] for further details. CO_2_ variation is common in coastal habitats [44]. However, the coastal waters where we collected *I. pygmaeus* show little daily variation in seawater *p*CO_2_ levels; average daily range 20.3 ± 8.6 µatm CO_2_ (mean ± SD) (Supplementary Text S1 and Figure S1). Thus, our experimental CO_2_ levels are ecologically relevant to the population of *I. pygmaeus* used.

### Behavioural analysis

After 7 days of current-day or elevated CO_2_ treatment, squid underwent sham treatment by being individually placed in 100 mL of aerated seawater from their CO_2_ treatment containing 0.2% ethanol for 30 min, as part of the experiment by Thomas, Spady [18]. Visually-guided behaviour was then tested for 15 min by placing squid individually in a tank (30 × 30 × 15 cm) filled to 3 cm depth with seawater from their CO_2_ treatment and with a mirror taking up the entire area of one wall. Three aspects of squid behaviour shown to be altered by elevated CO_2_ in Thomas, Spady [18], activity level, aggressive conspecific-directed behaviour and exploratory conspecific-directed behaviour (Table 1), were used here. See Thomas, Spady [18] for a detailed description of the behavioural analysis and results.

**Table 1:** Squid behaviours shown to be altered by OA in Thomas, Spady [18]

Behaviour	CO_2_ Treatment Effect
Activity	
Active time (s)	↑
Total distance moved (cm)	↑
Average speed (cm/s)	↑
**Visually-mediated aggressive conspecific-directed behaviour**	
Proportion of squid displaying one or more aggressive interactions	↑
Number of aggressive interactions per individual	↑
**Visually-mediated exploratory conspecific-directed behaviour**	
Time spent in Zone A (3 cm closest to the mirror)	↑
Proportion of squid displaying one or more exploratory interactions	↑
Number of exploratory interactions per individual	↑

These behaviours are used alongside transcriptomic data from the same individual squid within the current study. ↑ = increase in the behaviour at elevated compared with current-day CO_2_ conditions

### Tissue sample collection

Immediately after each behavioural trial, squid were euthanised with AQUI-S (1:1000). The head was separated from the mantle, rinsed in distilled water, and blotted dry. The skin, tentacles, beak, and buccal mass were removed and the eyes and central nervous system (CNS, containing the oesophagus running through the middle) were dissected and snap frozen in liquid nitrogen within 4.18 ± 0.55 (mean ± SD) minutes after euthanasia. Tissues were then transferred to -80^O^C for storage. This study was approved by and followed the animal ethics guidelines at James Cook University (JCU animal ethics number A2644).

### RNA extraction

Total RNA was extracted from the entire CNS, and both eyes combined for each squid (n_current−day CO2_ = 10, n_elevated CO2_ = 10 for each tissue) (Fig. 1). Each tissue sample was homogenised in RLT-Plus Buffer (Qiagen) with sterile zirconia/silica beads (1 mm diameter, BioSpec Products) in a Mini-BeadBeater 96 (BioSpec Products) for a total of 2 min. Total RNA was extracted using an AllPrep DNA/RNA Mini Kit (Qiagen). RNA integrity of all 40 samples was measured on an Agilent 2200 TapeStation (High Sensitivity RNA ScreenTape, Agilent) (Supplementary File S1).

### RNA sequencing

The Sequencing Section, Okinawa Institute of Science and Technology Graduate University, Japan carried out library preparation and sequencing on all 40 RNA samples. RNA was quantified by Qubit Flex Fluorometer (Qubit RNA BR assay kit, Thermo Fisher Scientific Inc.). The NEBNext^®^ Poly(A) mRNA Magnetic Isolation Module (New England BioLabs Inc.) was followed according to the manufacturers protocol to isolate mRNA. One library was prepared for each sample, using the NEBNext^®^ Ultra II Directional RNA Library Prep Kit for Illumina^®^ (New England BioLabs Inc.) following the manufacturers protocol, using ten PCR cycles. Libraries were sequenced on two lanes of a NovaSeq6000 with a S2 flow cell paired end to the length of 150 bp.

### RNA-seq read pre-processing

For a detailed workflow of the bioinformatic and statistical analyses, see Supplementary Figure S2. Raw reads were inspected with FastQC (v0.11.9) [45] and MultiQC (v1.9) [46] and trimmed with Fastp (v0.21.1) [47] using a sliding window of 4 bp, a mean Phred score of 30 and reads < 30 bp were trimmed. Kraken2 (v2.0.9) [48] was used with a confidence of 0.3 to remove any contamination using the NCBI bacterial and archaeal reference libraries (downloaded 08/2020).

### Read mapping and counting

As a reference for gene expression quantification, we created and annotated a *de novo* transcriptome assembly of *I. pygmaeus* CNS and eye tissues using long read PacBio ISO-sequencing data. Refer to Supplementary Text S2 for a description of the methods for ISO-sequencing, *de novo* transcriptome assembly, and transcriptome annotation. The transcriptome assembly (fasta file) can be found at NCBI BioProject PRJNA798187 https://www.ncbi.nlm.nih.gov/bioproject/?term=PRJNA798187+ [49] and the annotated transcriptome assembly (OmicsBox and csv files) are available from DOI 10.25903/ha66-mm11.

 [50]. The trimmed and decontaminated RNA-seq reads were mapped against the transcriptome assembly using salmon (v1.3.0) [51]. Correction for sequence-specific biases and fragment-level GC biases was used, the quantification step was skipped, and the flags ‘--validateMappings’ and ‘--hardFilter’ were also used. Corset (v1.09) [52] was run on the salmon equivalence class files from all 40 samples to cluster the transcripts to gene-level and produce gene-level counts. In Corset, we provided the four groups/treatments (eyes current-day CO_2_, eyes elevated CO_2_, CNS current-day CO_2_ and CNS elevated CO_2_), the log likelihood ratio test was switched off to prevent differentially expressed transcripts being split into different clusters, and the links between contigs were removed if the link was supported by less than 10 reads.

### Statistical analyses

All statistical analyses (as described below) were carried out in R (v4.0.4) [53], primarily using RStudio (v 1.4.1106) [54].

#### Differential expression analysis

DESeq2 (v1.30.1) [55] using the Wald test was used to compare gene expression between current-day and elevated CO_2_ conditions for the CNS and eyes separately. Genes with an adjusted p-value (padj, Benjamini-Hochberg method) < 0.05 were reported as differentially expressed (DE). Log2fold change estimates were shrunk with the ashr method [56] to increase their accuracy.

#### Gene set enrichment analysis

Gene set enrichment analysis (GSEA) was run in clusterProfiler (v3.18.1) [57] for each tissue separately to determine if sets of genes from the same gene ontology (GO) term/functional category showed significant, concordant differences between current-day and elevated CO_2_ conditions. Unweighted GSEA was run using the DESeq2 log2 fold-change values of all genes and the annotated GO terms as the ‘gene sets’. A minimum and maximum gene set size of 15 and 500, respectively, was used. GSEA determines if genes from the same functional category are significantly more likely to occur at the top or bottom of the log2 fold-change list and therefore whether these functional categories are up- or down-regulated at elevated CO_2_, respectively. P-values were adjusted for multiple comparisons using the Benjamini-Hochberg method and a significance threshold of padj < 0.05 was used. The GSEA results were imported into Cytoscape (v3.8.2) [58] where EnrichmentMap (v3.3.1) [59] was used to create a network to visualise the functional enrichment results. All significant functional categories were included in the network as a circular node. Functional categories with > 0.25 similarity were linked by edges. Similar functional categories were manually grouped into clusters and labelled.

#### Correlating gene expression profiles with CO_2_ treatment and OA-affected behaviours

To analyse the correlation between gene expression and behavioural traits of squid across CO_2_ treatments, we employed weighted gene co-expression network analysis (WGCNA) followed by canonical correlation analysis on the CNS and eyes, separately (Fig. 1). Refer to Supplementary Text S3 for a detailed description of the methods for gene co-expression network construction and module detection, module eigengene correlation with behavioural traits, module membership vs. gene significance, and identification of hub genes (Figures S3 – S21, Tables S1 and 2). Briefly, the gene-level counts from DESeq2 (v1.30.1) [55] were used in the WGCNA package (v1.70-3) [60] to construct a co-expression network and detect modules of genes. A module eigengene was calculated for each module of genes, representing the gene expression profiles of that module. Canonical correlation analysis using package CCA (v1.2.1) [61] was used to explore the correlations between the two sets of variables from the same individual squid: ME set = module eigengenes from each module; traits set = CO_2_ level (current-day or elevated) and behavioural traits (active time (s), distance (cm), speed (cm/s), time in Zone A (s), whether the squid displayed an exploratory/aggressive interaction (yes/no), number of exploratory/aggressive interactions). For those modules identified by canonical correlation analysis to be correlated with each trait, the Pearson correlation of module membership (MM, higher value indicates the gene is more highly connected to the given module) and gene significance (GS, higher value indicates a more biologically relevant gene) was calculated [60]. A correlation of GS and MM implies that genes more highly connected with a given module also tend to be more highly correlated with the given trait, providing another measure for the importance of this module with the given trait [60]. This identified the final modules of interest, within which the MM and GS values of each gene were used as a screening method to identify biologically relevant, highly interconnected hub genes [60, 62, 63], i.e. to find genes correlated with CO_2_ treatment and each behavioural trait. Hub genes were defined as those genes within the final modules of interest with a very strong correlation with the module (MM > 0.8) and a moderate correlation with the given trait (GS > 0.4).

All hub genes for CO_2_ treatment were compared across tissues to identify hub genes for CO_2_ treatment that are CNS-specific, eyes-specific or found in both tissues. Hub genes for CO_2_ treatment that were also a hub gene for one or more behavioural traits were identified as genes correlated with the associated OA-induced behavioural change. Finally, functional enrichment analysis was used for the CNS-specific CO_2_ treatment hub genes that were also hub genes for all three activity traits in the CNS using over-representation analysis (ORA) in clusterProfiler (v3.18.1) [57] with the hypergeometric test. This determined if any GO terms were significantly over-represented within the list of hub genes. All other groups of CO_2_ treatment hub genes had 25 or fewer genes and thus ORA was not used. P-values were adjusted for multiple comparisons using the Benjamini-Hochberg method and a significance threshold of padj < 0.05 was used.

## Results

### Transcriptome assembly and annotation

The *de novo* transcriptome assembly for *Idiosepius pygmaeus* was created from a total of 138.6 million PacBio ISO-sequencing subreads and resulted in 49,981 transcripts that were clustered into 27,420 genes. The transcriptome assembly had an N50 of 3,163 bp, 70.4% complete BUSCOs and an 82.1 ± 5.5% overall alignment rate of the RNA-seq reads (Table S3). A total of 69% of the transcripts received a functional annotation (Supplementary Table S4). The species distribution of the top blast hits was dominated by cephalopod species (Figure S22). Final mapping of RNA-seq reads against the transcriptome assembly had a 73.6 ± 6.9% mapping rate (Table S5).

### Differentially expressed genes

**Fig. 2:**
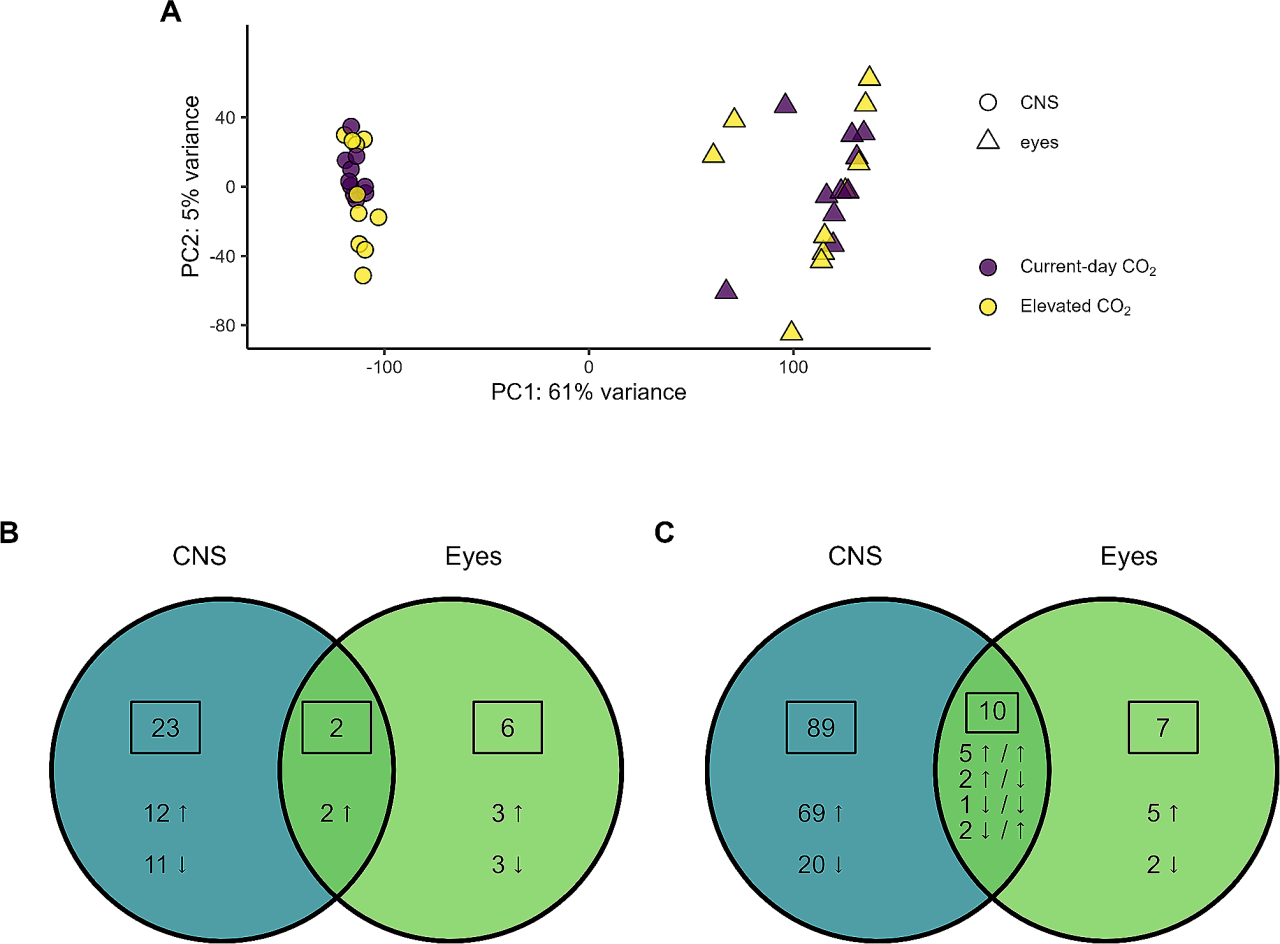
Differential expression and GSEA results. **A** PC1 and PC2 axes from the principal components analysis of all genes for the 40 samples. **B** Venn diagram comparing the DEGs between current-day and elevated CO_2_ levels in the CNS and eyes. **C** Venn diagram comparing the GO terms / functional categories found to be significantly different between current-day and elevated CO_2_ levels in the CNS and eyes by GSEA. Ο = CNS, △ = Eyes, ↑ = upregulated at elevated CO_2_ conditions, ↓ = downregulated at elevated CO_2_ conditions, purple = current-day CO_2,_ yellow = elevated CO_2_.

We compared gene expression between current-day and elevated CO_2_ conditions for the CNS and eyes separately. There was more variance in the eyes than the CNS (Fig. 2A). In the CNS, we identified 25 differentially expressed genes (DEGs) between current-day and elevated CO_2_ conditions; 14 upregulated and 11 downregulated with elevated CO_2_. In the eyes, there were eight DEGs; five upregulated and three downregulated at elevated, compared to current-day, CO_2_ conditions (Fig. 2B). See Figure S23 for a heatmap of all samples showing the expression pattern of all genes in the CNS and eyes, and Figure S24 for volcano plots of the CNS and eyes. Two genes were significantly upregulated with elevated CO_2_ in both the CNS and eyes; one essential for autophagy (*ykt6*) and another poorly characterised gene (*fam204a*). In both tissues, the DEGs play roles in neurotransmission (CNS: *folh1*, *syvn1-b*, *slc2a13*, *celsr3*, eyes: *maoa*, *slc18a1*, *cbs*), immune function (CNS: *psenen*, *syvn1*-*b, map4k5*, *tf*, nme6, *map1l3ca/b*, eyes: *pglyrp2*, *cbs, maoa*), the oxidative stress response (CNS: *tf*, *cyb561d2, syvn1-b*, *chrac1, ykt6*, eyes: *cbs, ykt6*), and transcription regulation (CNS: *nme6, chrac1, znf271*, eyes: *gtf2e2*) (Table S6).

### Small, coordinated changes in expression of genes belonging to the same functional categories

**Fig. 3:**
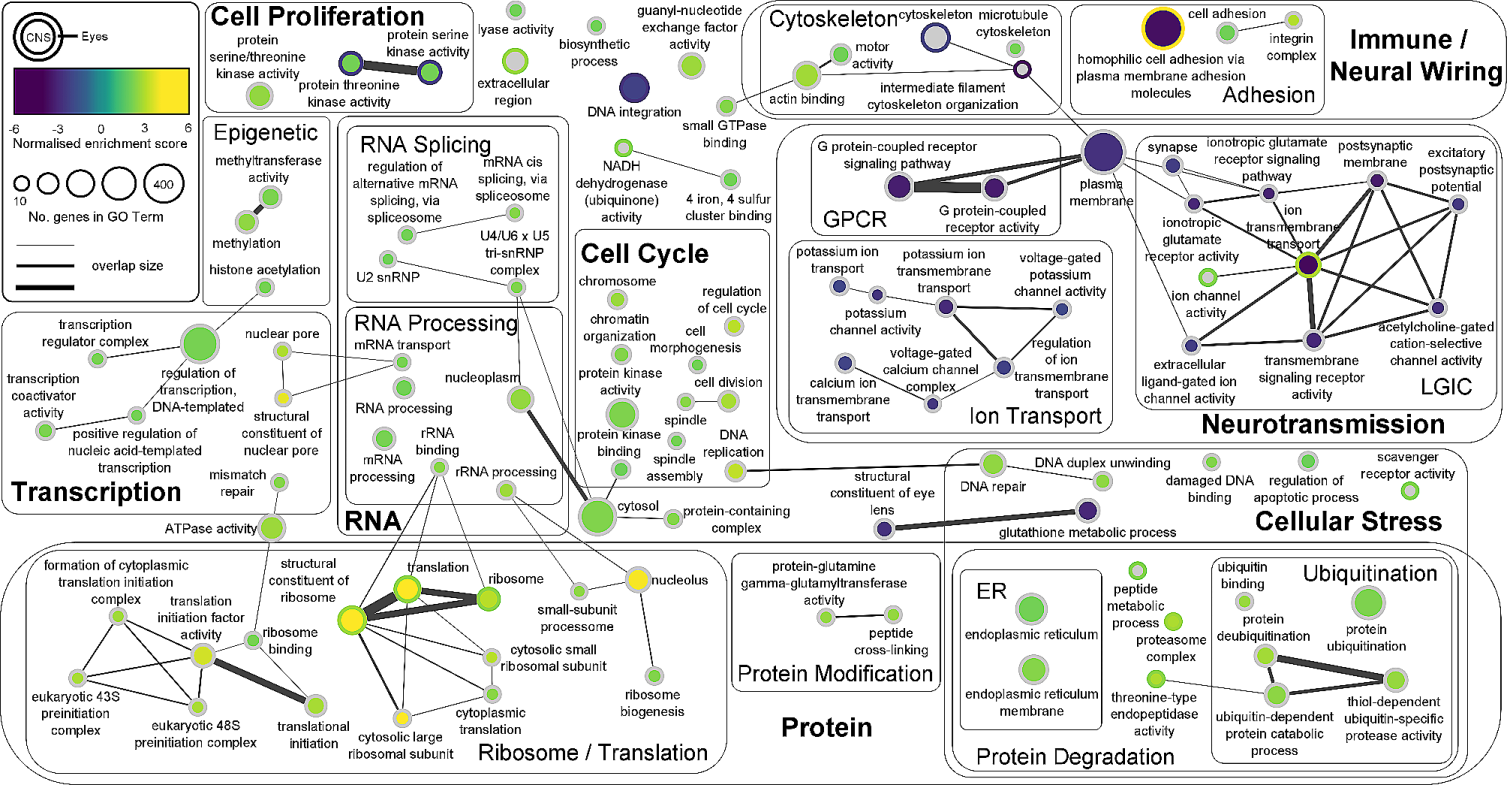
Enrichment map displaying the gene set enrichment analysis (GSEA) results in both the CNS and eyes. Significant GO terms/functional categories are represented by a circular node. Results from the CNS and eyes are represented by colouration of the inner node area and node border, respectively. Yellow represents functional categories upregulated at elevated CO_2_ and purple represents functional categories downregulated at elevated CO_2_. Colour represents the normalised enrichment score and node size the number of core enrichment genes in each functional category. Functional categories found not significant (padj > 0.05) are coloured grey for the corresponding tissue type. The nodes from functional categories with a similarity > 0.25 are connected by an edge, with edge width increasing with increasing similarity (increasing number of genes shared by the functional categories). Similar functional categories were manually grouped into clusters and assigned a label

Gene set enrichment analysis (GSEA) identified Gene Ontology (GO) terms/functional categories, across all three GO categories (biological process, molecular function, and cellular component), that were significantly affected by CO_2_ treatment, indicating small, coordinated changes in expression of the genes belonging to each of these functional categories. We identified ninety-nine significant functional categories in the CNS; 75 upregulated and 24 downregulated at elevated CO_2_ (Fig. 2C). These functional categories included those involved in transcription, RNA processing, translation, protein processing, the cell cycle and cell proliferation, and neurotransmission (Fig. 3 and S25). There were 17 significant functional categories in the eyes; 12 upregulated and 5 downregulated at elevated CO_2_ (Fig. 2C). Ten functional categories were significantly affected by CO_2_ treatment in both the CNS and eyes including functions related to the ribosome and translation, ion channels, kinase activity, protein degradation and cell adhesion (Fig. 3 and S25).

### Genes correlated with OA-induced behavioural change

**Fig. 4:**
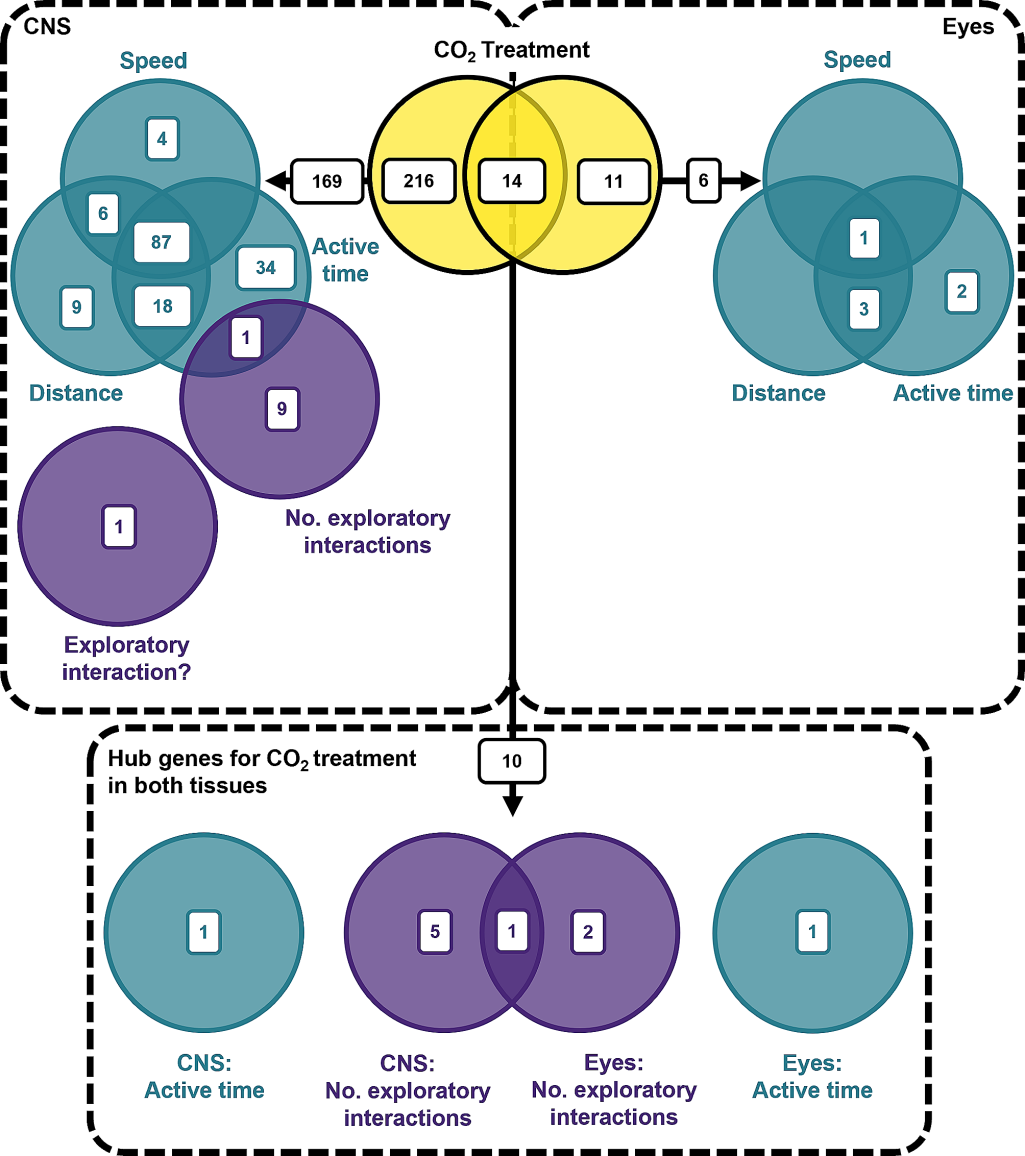
Venn diagram depicting the number of hub genes identified for CO_2_ treatment and behavioural traits in the CNS and eyes. The yellow Venn diagram in the centre depicts the number of hub genes for CO_2_ treatment that are CNS-specific (left) and eyes-specific (right), and the overlap represents the number of CO_2_ treatment hub genes shared by both tissues. CNS-specific and eyes-specific CO_2_ treatment hub genes also identified as a hub gene for one or more behavioural traits in the CNS or eyes are on the left and right, respectively. Hub genes for CO_2_ treatment found in both tissues, that are also a hub gene for a behavioural trait in one or both tissues, are shown at the bottom centre. CO_2_ treatment hub genes shared with activity traits (active time, distance and speed) and exploratory conspecific-directed behaviours (number of exploratory interactions and whether any exploratory interactions occurred) are in teal and purple, respectively. Exploratory interaction? = whether any exploratory interactions occurred (yes/no)

**Fig. 5:**
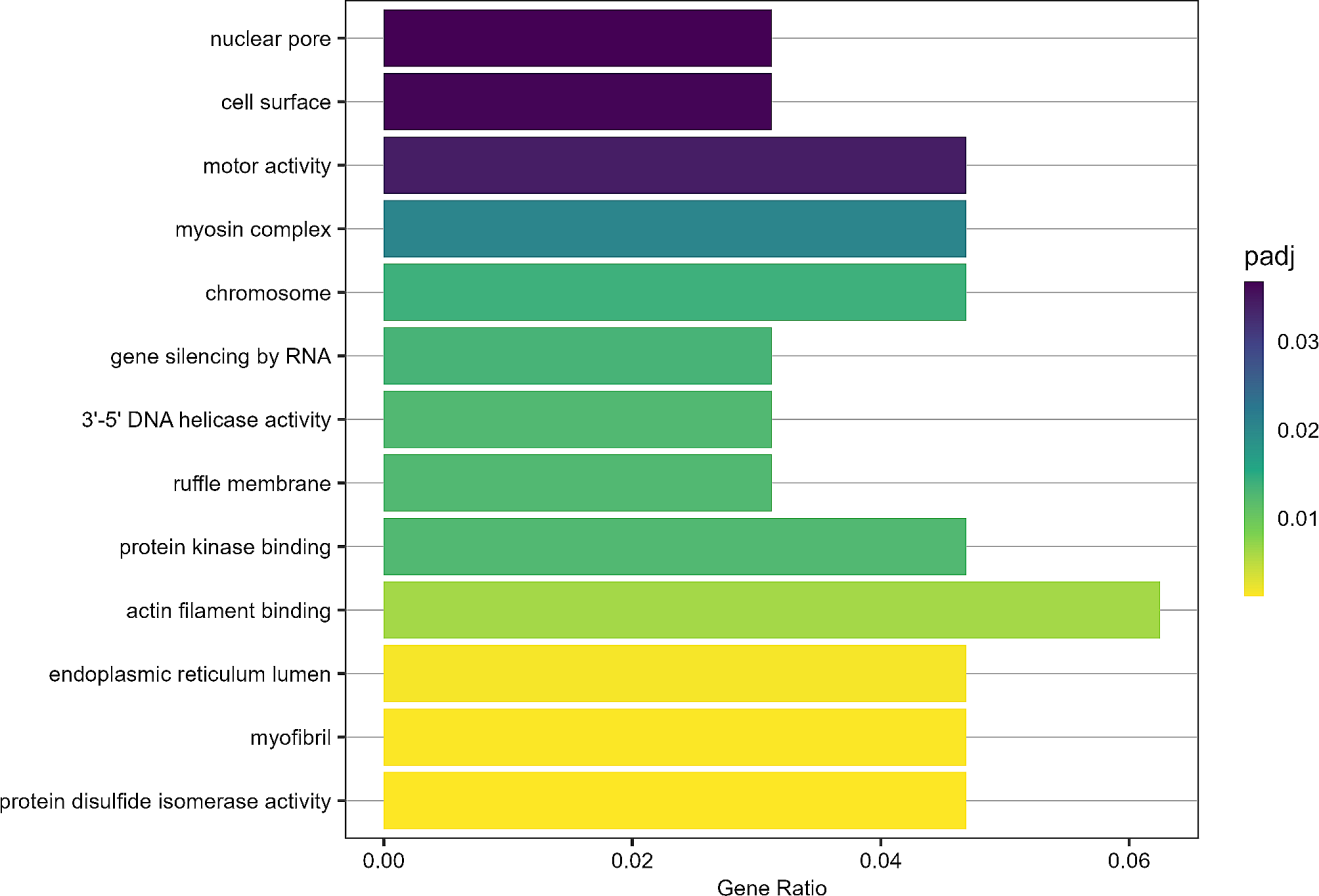
GO Terms/functional categories significantly enriched from the 87 genes positively correlated with CO_2_ treatment and all three activity traits in the CNS. Functional categories are ordered by their adjusted p-value. padj = adjusted p-value, yellow indicates a lower adjusted p-value / higher significance. Gene Ratio = the number of genes represented in the genes positively correlated with CO_2_ treatment and all three activity traits in the CNS, in comparison to all of the genes in the CNS.

We identified 230 and 25 hub genes for CO_2_ treatment in the CNS and eyes, respectively, with 14 CO_2_ treatment hub genes shared by both tissues. Of these CO_2_ treatment hub genes in the CNS, eyes and both tissues, 169, 6 and 10 genes were also identified as hub genes for one or more behavioural traits, respectively, indicating these genes as potentially correlated with CO_2_-induced behavioural changes (Fig. 4). Of the 169 genes in the CNS potentially contributing to CO_2_-induced behavioural changes, 87 were positively correlated with CO_2_ treatment and all three activity traits and were significantly enriched for 13 functional categories, including those playing a role in the cell cycle, cell migration, and protein synthesis and folding (Fig. 5). Four of these functional categories were also identified as significantly upregulated at elevated CO_2_ in the CNS by GSEA; ‘nuclear pore’, ‘motor activity’, ‘chromosome’ and ‘protein kinase binding’. The 6 transcripts in the eyes potentially contributing to CO_2_-induced behavioural changes had a match for two known genes; an acetylcholine receptor subunit (*chrna10*) and a gene essential for maintaining retinal tissue integrity (*crb*). The 10 transcripts in both tissues potentially contributing to CO_2_-induced behavioural changes had a match for eight known genes, again including *chrna10*, and genes with putative roles in cell proliferation (*gid-4, cdk10*) and protein processing (*srp72, vhl, zranb1*). See Table S7 – S10 for all hub genes identified by WGCNA and their putative functions.

### OA-affected genes and their functions

Genes affected by elevated CO_2_ and correlated with OA-induced behavioural change were identified in seven main functions: neurotransmission; cell cycle; cell proliferation and differentiation; neural wiring; transcription, RNA processing and protein processing; immune function; oxidative stress.

#### Neurotransmission

A range of genes and functional categories involved in various types of neurotransmission were significantly affected by CO_2_ treatment (Table 2). We identified small, coordinated downregulation of genes belonging to a cluster of nine functional categories in the CNS, and upregulation of two functional categories in the eyes, involved in ligand-gated ion channel-mediated neurotransmission. The genes contributing most to the up-/down-regulation of each of these functional categories (core enrichment genes) in the CNS and eyes included genes for components of acetylcholine, GABA_A_ and glutamate ion channel receptors (Table S11). There was also small, coordinated downregulation in the CNS of genes belonging to two functional categories involved in G protein-coupled receptor (GPCR)-mediated neurotransmission, including genes coding for components of metabotropic glutamate, GABA_B_, serotonin and dopamine receptors (Table S12). Notably, a subunit of nicotinic acetylcholine receptors (*chrna10*) was correlated with CO_2_ treatment and behaviours in both tissues. *Chrna10* as well as other genes coding for nicotinic acetylcholine receptor subunits (*chrna1*, *chrna3*, *chrna5*, and *chrnb1*) were core enrichment genes in eight functional categories significantly affected by CO_2_ treatment in both tissues. Genes coding for regulation of GABAergic neurotransmission were upregulated in both tissues (CNS: *syvn1-b*, eyes: *slc18a2*), and positively correlated with CO_2_ treatment and behaviours in the CNS (*phf24*, *rac1*), while *aldh5a1*, which is involved in the final degradation step of GABA [64], was negatively correlated with CO_2_ treatment and behaviours in the CNS. Furthermore, there was upregulation of *folh1* (glutamate synthesis) and downregulation of *celsr3* (mediator of glutamatergic synapse formation) in the CNS. There was also upregulation in the eyes of two key genes involved in monoaminergic (dopamine, serotonin (5-HT), norepinephrine, and epinephrine) neurotransmission (*maoa, slc18a2*).

**Table 2:** Results across all three analysis methods in the CNS and eyes related to neurotransmission

	DE			GSEA			WGCNA				
	Gene	CNS	Eyes	GO Term / Functional Category	CNS	Eyes	Gene	CNS		Eyes	
								CO_2_	Behaviour	CO_2_	Behaviour
**Neurotransmission**											
**Cholinergic**				‘acetylcholine-gated cation-selective channel activity’, ‘synapse*’, ‘postsynaptic membrane*’, ‘excitatory postsynaptic potential*’, ‘transmembrane signaling receptor activity*’, ‘extracellular ligand-gated ion channel activity*’	↓		*chrna10*	↓	↑	↑/↓	↓
				‘ion transmembrane transport*’	↓	↑					
				‘ion channel activity*’		↑					
**GABAergic**	*syvn1-b*	↑		‘extracellular ligand-gated ion channel activity*’, ‘G protein-coupled receptor signaling pathway*’	↓		*phf24, rac1*	↑	↑		
	*slc18a2*		↑	‘ion transmembrane transport*’	↓	↑	*aldh5a1*	↓	↓		
**Glutamatergic**	*folh1*	↑		‘ionotropic glutamate receptor signalling pathway’, ‘ionotropic glutamate receptor activity’, ‘synapse*’, ‘postsynaptic membrane*’, ‘’G protein-coupled receptor signaling pathway*’, ‘G protein-coupled receptor activity*’	↓						
	*celsr3*	↓		‘ion transmembrane transport*’	↓	↑					
				‘ion channel activity*’		↑					
**Monoaminergic**	*maoa, slc18a2*		↑	‘G protein-coupled receptor signaling pathway*’, ‘G protein-coupled receptor activity*’	↓						
**General Processes for Synaptic Neurotransmission**				‘potassium channel activity’, ‘potassium ion transmembrane transport’, ‘voltage-gated potassium channel activity’, ‘regulation of ion transmembrane transport’, ‘voltage-gated calcium channel complex’, ‘calcium ion transmembrane transport’	↓	↓	*futsch, dgkq*	↓	↓		
**GPCR Trafficking**							*tmed2*	↑	↑		

Genes identified as significantly differentially expressed (DE) between current-day and elevated CO_2_ conditions, GO Terms/functional categories significantly affected by elevated CO_2_ treatment as identified by gene set enrichment analysis (GSEA), and genes identified as correlated with both CO_2_ treatment and one or more OA-affected behaviours by weighted gene co-expression network analysis (WGCNA). DE: ↑ or ↓ = significant upregulation or downregulation of this gene at elevated compared to current-day CO_2_ conditions. GSEA: ↑ or ↓ = significant upregulation or downregulation of this GO Term/functional category, indicating small, coordinated upregulation or downregulation of the genes belonging to this functional category, at elevated compared to current-day CO_2_ conditions. WGCNA: CO_2_ ↑ or ↓ = positive or negative correlation between the expression of the gene and CO_2_ treatment, Behaviour ↑ or ↓ = positive or negative correlation between the expression of the gene and 1 or more OA-affected behaviours, ↑/↓ = different transcripts of the same gene were found to be positively and negatively correlated. * = GO Term contains core enrichment genes for this type of neurotransmission

Genes involved in the general processes required for synaptic neurotransmission also displayed altered expression after elevated CO_2_ exposure (Table 2). There was small, coordinated downregulation in the CNS of genes belonging to a cluster of seven functional categories involved in K^+^, Ca^2+^ and Na^+^ ion transport important for general processes involved in neurotransmission, including maintenance of membrane potential, action potential generation and neurotransmitter release (Table S13). Furthermore, genes involved in regulating synaptic neurotransmission (*futsch*, *dgkq*) were negatively correlated with CO_2_ treatment and activity traits in the CNS.

#### Cell cycle

In the CNS, we found small, coordinated upregulation of genes belonging to functional categories involved in the cell cycle, and genes positively correlated with CO_2_ treatment and all three activity traits were enriched for cell cycle functional categories (Table 3; Figs. 3 and 5). This included genes and functional categories involved in regulating the cell cycle, as well as those specifically involved in the cell cycle stages of interphase and mitosis. In particular, there was small, coordinated upregulation of genes belonging to the functional categories ‘chromosome’ and ‘protein kinase binding’ (Fig. 3), and these same functions were enriched in the genes positively correlated with CO_2_ treatment and all three activity traits (Fig. 5).

**Table 3:** Results across all methods related to the cell cycle, cell proliferation and differentiation, neural wiring

	DE			GSEA			WGCNA				
	Gene	CNS	Eyes	GO Term / Functional Category	CNS	Eyes	Gene	CNS		Eyes	
								CO_2_	Behaviour	CO_2_	Behaviour
**Cell cycle**											
**Interphase: G1 phase**							*cdt1, snd1*	↑	↑		
**Interphase: S phase**				‘DNA replication’	↑		*tubg1, nat10, psf2, cdt1, mcm5, dbf4, spt16, foxm1, bptf*	↑	↑		
**Interphase: G2 phase**							*ranbp2, donson*	↑	↑		
**Mitosis**				‘chromosome’, ‘chromatin organization’	↑		*ncaph, smc4, ttk, incenp, bub1, foxm1, cdt1*	↑	↑		
				‘spindle’, ‘spindle assembly’	↑		*bub3*	↑	↑		
				‘cell division’	↑		*zip, myh9/10, myl9, anln, prpf40a*	↑	↑		
**Cell cycle regulation**				‘regulation of cell cycle’, ‘protein kinase binding’, ‘protein kinase activity’	↑		*foxm1, eif3b, ccnb3, melk, ttk, bub1, dbf4, anb32a, klf10, ecd*	↑	↑		
							*stk10, calm*	↓	↓		
**Cell proliferation and differentiation**											
**Cell proliferation**				‘protein serine/threonine kinase activity’	↑		*ttk, eif3b, foxm1, melk, dbf4, srrt, tk1, tgfb1i1, pa2g4, ttc3, ptpr*	↑	↑		
				‘protein serine kinase activity’, ‘protein threonine kinase activity’	↑	↓	*gid-4*	↑		↑	↓
							*bcar3*	↓	↑		
**Cell differentiation**							*eif3b, rac1, ptpr, tbc1d1, tgfb1i1, itga4, pa2g4, slc4a11*	↑	↑		
							*btg1*	↓	↓		
**Neuronal differentiation**							*ttc3, rac1*	↑	↑		
**Neurogenesis**							*ncaph*	↑	↑		
							*cdk10*	↑/↓	↓	↓	↑
**Neural Wiring**											
**Cell migration**				‘motor activity’, ‘actin binding’	↑		*arpc5, anln, myl9, slit3, mbtp, prpf40a*	↑	↑		
							*dag*	↓	↓		
							*zranb1*	↓	↑	↓	↑
**Cell adhesion**				‘cell adhesion’, ‘integrin complex’	↑		*itga4, itga9, slc4a11, pcdh1, vcl, rac1, ptpr, pa2g4*	↑	↑		
				‘homophilic cell adhesion via plasma membrane adhesion molecules’	↓	↑	*pcdh1*	↓	↓		
**Neurite growth and synapse formation**							*adgrb3, rac1, apbb1, ptpr*	↑	↑		
							*futsch*	↓	↓		

Results are shown for all three analysis methods in the CNS and eyes. Genes identified as significantly differentially expressed (DE) between current-day and elevated CO_2_ conditions, GO Terms / functional categories significantly affected by elevated CO_2_ treatment as identified by gene set enrichment analysis (GSEA), and genes identified as correlated with both CO_2_ treatment and one or more OA-affected behaviours by weighted gene co-expression network analysis (WGCNA). See Table 2 for a description of the symbols

#### Cell proliferation and differentiation

Genes involved in cell proliferation and differentiation, including specifically for neuronal differentiation and neurogenesis were mostly positively correlated with CO_2_ treatment and behaviours in the CNS (Table 3). Genes involved in neuronal differentiation (*ttc3*), neural stem cell self-renewal (*srrt*), neural progenitor proliferation (*melk*), and neurogenesis (*ncaph, adgrb3*) were all positively correlated with CO_2_ treatment and activity traits. *Bcar3*, which promotes cell proliferation, migration and redistribution of actin fibres, and *psap*, which acts as a neurotrophic and myelinotrophic factor, were negatively correlated with CO_2_ treatment and positively correlated with number of exploratory interactions in the CNS. Several *cdk10* transcripts were identified as correlated with CO_2_-induced behavioural change. *Cdk10* codes for a protein kinase that plays pivotal roles in controlling a range of fundamental cellular processes including cell proliferation and neurogenesis (reviewed in Guen, Gamble [65]).

#### Neural wiring

A range of genes and functional categories involved in neural wiring, including cell migration and adhesion, and neurite growth and synapse formation were affected by CO_2_ treatment, also mostly exhibiting an upregulation in the CNS (Table 3). There was small, coordinated upregulation of genes involved in cell migration functional categories, including ‘motor activity’, ‘actin binding’, ‘cell adhesion’ and ‘integrin complex’. Those genes positively correlated with CO_2_ treatment and all three activity traits in the CNS were enriched for similar functional categories: ‘actin filament binding’, ‘myosin complex’, ‘myofibril’, ‘motor activity’ and ‘ruffle membrane’. Genes with a role specifically in neuron migration and adhesion, and the related processes of dendrite and axon outgrowth and branching, dendritic spine formation, and synapse formation were also positively correlated with CO_2_ treatment and activity (*rac1*, *ptpr, adgrb3, apbb1*). One gene involved in neurite growth and branching (*futsch*) was negatively correlated with CO_2_ treatment and activity traits.

#### Transcription, RNA processing, and protein processing

There was generally an upregulation of genes and functional categories involved in transcription, RNA processing and protein processing in the CNS, and to a smaller extent in the eyes (Table 4). In the CNS, there was small, coordinated upregulation of genes belonging to functional categories involved in transcription, including ‘DNA duplex unwinding’, and its’ daughter term ‘3’-5’ DNA helicase activity’ was enriched in those genes positively correlated with CO_2_ treatment and all three activity traits (Fig. 5). There was small, coordinated upregulation in the CNS of genes belonging to the functional category ‘nuclear pore’, and ‘nuclear pore’ was also enriched for those genes positively correlated with CO_2_ treatment and all three activity traits in the CNS (Fig. 5). Genes involved in transcription were also DE; *nme6, chrac1* and *znf271* were upregulated in the CNS, and *gtf2e2* downregulated in the eyes. There was small, coordinated upregulation of genes involved in ‘RNA processing’, ‘mRNA processing’ and ‘rRNA processing’, as well as functional categories involved in the processes and components of the spliceosome, which excises introns to produce mature mRNA (Fig. 3). Components of the spliceosome (*snrpa*, *snrnp200*) were also identified as correlated with CO_2_ treatment and behaviours (Table 4).

**Table 4:** Results across all methods related to transcription, RNA processing and protein processing

	DE			GSEA			WGCNA				
	Gene	CNS	Eyes	GO Term / Functional Category	CNS	Eyes	Gene	CNS		Eyes	
								CO_2_	Behaviour	CO_2_	Behaviour
**Transcription**	*chrac1, znf271*	↑		‘DNA duplex unwinding’, ‘nuclear pore’, ‘regulation of transcription, DNA-templated’, ‘positive regulation of nucleic acid-templated transcription’, ‘transcription regulator complex’, ‘transcription coactivator activity’	↑		*polr1a, spt16, bptf, arpc5, nup160, nup155, nup205*	↑	↑		
	*nme6, gtf2e2*	↓									
**RNA**											
**RNA Processing**				‘RNA processing’, ‘mRNA processing’, ‘rRNA processing’, ‘mRNA transport’, ‘rRNA binding’	↑		*cstf1, exosc10, nat10, pa2g4*	↑	↑		
**RNA Splicing**				‘regulation of alternative mRNA splicing, via spliceosome’, ‘mRNA cis splicing, via spliceosome’, ‘U2snRNP’, ‘U4/U6 x U5 tri-snRNP complex’	↑		*melk, snrpa, ecd, prpf40a*	↑	↑		
							*snrnp200*	↑/↓	↑/↓		
**Protein**											
**Translation**				‘translation’	↑	↑	*eif3b, eif3d, eef1g*	↑	↑		
				‘translation initiation’, ‘translation initiation factor activity’, ‘eukaryotic 48S preinitiation complex’, ‘eukaryotic 43S preinitiation complex’, ‘formation of cytoplasmic translation initiation complex’, ‘cytoplasmic translation’	↑						
**Ribosome**				‘ribosome’, ‘structural constituent of ribosome’	↑	↑	*rpl23a, rpl4, rpl7l, rps27a, nop58*	↑	↑		
				‘cytosolic small ribosomal subunit’, ‘cytosolic large ribosomal subunit’, ‘small-subunit processome’, ‘ribosome biogenesis’, ‘ribosome binding’	↑						
**Endoplasmic reticulum**				‘endoplasmic reticulum’, ‘endoplasmic reticulum membrane’	↑						
**Protein trafficking**							*stt3a, tmed2, rpn2, rpn1*	↑	↑		
							*srp72*	↓		↓	↑
**Protein modification**				‘peptide cross-linking’, ‘protein-glutamine gamma-glutamyltransferase activity’	↑						
**Protein folding**							*hspa5, pdia3, pdia4, pdia5, ssr1, ganab, ppib*	↑	↑		
**Protein degradation: ubiquitination and proteasome**	*syvn1-b*	↑		‘proteasome complex’	↑	↑	*rpn1, ttc3, cblb*	↑	↑		
				‘peptide metabolic process’		↑	*vhl*	↓	↑	↓	
				‘protein ubiquitination’, ‘protein deubiquitination’, ‘ubiquitin binding’, ‘ubiquitin-dependent protein catabolic process’, ‘thiol-dependent ubiquitin-specific protease activity’	↑		*derl1*	↓	↑		
**Protein degradation: lysosomal**	*ykt6*	↑	↑				*tmcc1/2*	↓	↓		

Results are shown for all three analysis methods in the CNS and eyes. Genes identified as significantly differentially expressed (DE) between current-day and elevated CO_2_ conditions, GO Terms/functional categories significantly affected by elevated CO_2_ treatment as identified by gene set enrichment analysis (GSEA), and genes identified as correlated with both CO_2_ treatment and one or more OA-affected behaviours by weighted gene co-expression network analysis (WGCNA). See Table 2 for a description of the symbols

A range of genes and functional categories involved in protein synthesis, folding and degradation/turnover were significantly upregulated in the CNS, and some were also upregulated in the eyes. Small, coordinated upregulation of genes involved in the initiation and process of translation as well as in functional categories for ribosomal components occurred in both tissues (Fig. 3). Genes with similar functions were also positively correlated with CO_2_ treatment and activity traits in the CNS, including translation initiation factors (*eif3b*, *eif3d*), an elongation factor (*eef1g*), and ribosomal components (*rpl23a*, *rpl4*, *rpl7l*, *rps27a*). Genes involved in protein translocation (*stt3a, rpn1, rpn2, tmed2*), and protein folding and quality control (*pdia3, pdia4 and pdia5, hspa5*) were correlated with CO_2_ treatment and behaviours in the CNS. The endoplasmic reticulum associated degradation (ERAD) pathway involves protein ubiquitination followed by proteasomal degradation and we found a range of genes involved in this process to be affected by CO_2_ treatment. There was small, coordinated upregulation of genes in the ‘endoplasmic reticulum’ and five ubiquitin-related functional categories in the CNS, of ‘proteasome complex’ in both tissues, and of ‘peptide metabolic process’ in the eyes. Genes positively correlated with CO_2_ treatment and all three activity traits were enriched for ‘endoplasmic reticulum lumen’ and included genes coding for E3 ubiquitin ligases (*cblb, ttc3*) and a subunit for the 26S proteasome (*rpn1*). A gene coding for an E3 ubiquitin ligase (*syvn1-*b) was also upregulated in the CNS. Lysosomal degradation is another method for protein turnover, and a gene essential for lysosomal function (*ykt6*) was upregulated in both the CNS and eyes.

#### Immune function

Genes and functional categories involved in the three stages of the innate immune response (sensing, signalling and effectors), were affected by CO_2_ treatment in both tissues (Table 5). In the eyes, the immune sensor molecule *pglyrp2* was downregulated, while there was small, coordinated upregulation of genes belonging to the functional category ‘scavenger receptor activity’ which binds pathogens. Genes that mediate the immune response via cellular signalling pathways were DE, including upregulation of *map4k5/3* and *syvn1-b*, and downregulation of *psenen* in the CNS. In the eyes, there was upregulation of *maoa*, which plays a key role, via norepinephrine signalling, in the molluscan neuroendocrine-immune axis-like pathway [66–68]. Genes involved in the activation and production of immune effectors were also affected by CO_2_ treatment. In particular, *tf* coding for transferrin, which sequesters iron so it is unavailable for pathogens and is a key component of the molluscan innate immune response, including in squid [69–73], was upregulated and positively correlated with CO_2_ treatment and activity traits in the CNS. The key molecular marker of autophagy, *map1l3ca/b*, which plays an important role in the molluscan immune response [74–76] was downregulated in the CNS. There was also small, coordinated upregulation of genes belonging to functional categories involved in mediating phagocytosis, including ‘cell adhesion’ and ‘integrin complex’. Genes involved in cell adhesion as part of the immune response (*itga4, itga9, rac1, ptpr*) were also positively correlated with CO_2_ treatment and activity traits. The cytoskeleton is also important for phagocytosis and there was small, coordinated upregulation of genes in three cytoskeleton-related functional categories in the CNS (‘motor activity’, ‘actin binding’, and ‘microtubule cytoskeleton’), and two downregulated in the eyes (‘cytoskeleton’ and ‘intermediate filament cytoskeleton organisation’) (Fig. 4).

**Table 5:** Results across all analysis methods in the CNS and eyes related to the immune response

	DE			GSEA			WGCNA				
	Gene	CNS	Eyes	GO Term / Functional Category	CNS	Eyes	Gene	CNS		Eyes	
								CO_2_	Behaviour	CO_2_	Behaviour
**Immune**											
**Sensor**	*pglyrp2*		↓	‘scavenger receptor activity’		↑					
**Immune signalling pathways**	*map4k5/3, syvn1-b*	↑					*abhd12*	↑	↑		
	*psenen*	↓									
	*cbs, maoa*		↑								
**Effector: Controlling resources required by pathogens**	***tf***, *nme6*	↑					***tf***	↑	↑		
**Effector: Autophagy**	*map1l3ca/b*	↓									
**Effector: Phagocytosis**				‘cell adhesion’, ‘integrin complex’, ‘motor activity’, ‘actin binding’, ‘microtubule cytoskeleton’	↑		*itga4, itga9, rac1, ptpr*	↑	↑		
				‘cytoskeleton’, ‘intermediate filament cytoskeleton organisation’		↓					

Genes identified as significantly differentially expressed (DE) between current-day and elevated CO_2_ conditions, GO Terms/functional categories significantly affected by elevated CO_2_ treatment as identified by gene set enrichment analysis (GSEA), and genes identified as correlated with both CO_2_ treatment and one or more OA-affected behaviours by weighted gene co-expression network analysis (WGCNA). See Table 2 for a description of the symbols

#### Oxidative stress

Genes involved in regulating reactive oxygen species (ROS) and antioxidant production were differentially expressed in both issues; *tf* (CNS: upregulated and positively correlated with CO_2_ treatment and activity traits), *cyb561d2* (CNS: downregulated), *cbs* (eyes: upregulated). Furthermore, *scl4a11*, which regulates the oxidative stress response, was positively correlated with CO_2_ treatment and activity traits in the CNS, and there was small, coordinated downregulation in the CNS of genes involved in ‘glutathione metabolic process’ (Table 6). Genes and functional categories involved in response to oxidative damage were also affected by CO_2_ treatment (Table 6). Genes involved in DNA repair were generally upregulated with CO_2_ treatment in the CNS; *chrac1* was significantly upregulated, there was small, coordinated upregulation of ‘damaged DNA binding’ and ‘DNA repair’, *spt16, foxm1, bptf*, and *arpc5* were positively correlated with CO_2_ treatment and activity traits, and *nit1*, whose loss of expression promotes resistance to DNA damage stress, was negatively correlated with CO_2_ treatment and activity traits. Genes involved in protein damage control and endoplasmic reticulum stress were also affected by CO_2_ treatment; *ykt6*, which is essential for autophagy was upregulated in the CNS and eyes, and the heat shock protein *hspa5*, which is a key repressor of the unfolded protein response was positively correlated with CO_2_ treatment and activity traits. Furthermore, genes that induce apoptosis in response to DNA damage (*apbb5*) and ER stress (*tmem214-b*), were positively correlated with CO_2_ treatment and activity traits. There was also small, coordinated upregulation in the CNS of genes involved in the ‘regulation of apoptotic process’.

**Table 6:** Results across all three analysis methods in the CNS and eyes related to cellular stress

	DE			GSEA			WGCNA				
	Gene	CNS	Eyes	GO Term / Functional Category	CNS	Eyes	Gene	CNS		Eyes	
								CO_2_	Behaviour	CO_2_	Behaviour
**Oxidative Stress**											
**Antioxidant and ROS production**	***tf***	↑					***tf***, *slc4a11*	↑	↑		
	*cyb561d2*	↓		‘glutathione metabolic process’	↓						
	*cbs*		↑								
**DNA damage and repair**	*chrac1*	↑		‘damaged DNA binding’, ‘DNA repair’	↑		*spt16, foxm1, bptf, arpc5*	↑	↑		
							*nit1*	↓	↓		
**Protein damage and ER stress**	*ykt6*	↑	↑	‘endoplasmic reticulum’, ‘endoplasmic reticulum membrane’, ‘protein ubiquitination’, ‘protein deubiquitination’, ‘ubiquitin binding’, ubiquitin-dependent protein catabolic process’, ‘thiol-dependent ubiquitin-specific protease activity’	↑		*hspa5*	↑	↑		
				‘proteasome complex’	↑	↑					
				‘peptide metabolic process’		↑					
**Cellular stress-induced apoptosis**	*syvn1-b*	↑		‘regulation of apoptotic process’	↑		*apbb5, tmem214-b*	↑	↑		

Genes identified as significantly differentially expressed (DE) between current-day and elevated CO_2_ conditions, GO Terms/functional categories significantly affected by elevated CO_2_ treatment as identified by gene set enrichment analysis (GSEA), and genes identified as correlated with both CO_2_ treatment and one or more OA-affected behaviours by weighted gene co-expression network analysis (WGCNA). See Table 2 for a description of the symbols

## Discussion

As the nervous system forms the fundamental link between animals and the environments they inhabit [3, 4], understanding the neurobiological impacts of environmental change is key to predicting how and why animals will respond to anthropogenic climate change. In this study, we sought to understand how projected end-of-century CO_2_ levels alter the nervous system at a molecular level, and how such changes may affect behaviour of the whole animal. To do this, we investigated the transcriptomic response to ocean acidification (OA) of the central and peripheral nervous system of a marine invertebrate with a complex nervous system, the two-toned pygmy squid *Idiosepius pygmaeus*. We then correlated patterns of gene expression with CO_2_ treatment levels and OA-affected behaviours in the same individuals. The central nervous system (CNS) and eyes of *I. pygmaeus* responded to elevated CO_2_ with significant differential expression (DE) of a small number of genes, and widespread small but coordinated changes of genes belonging to important functional categories between CO_2_ conditions. Furthermore, we identified 169 genes in the CNS, six genes in the eyes and ten genes in both tissues that were correlated with CO_2_ treatment and one or more behaviours affected by OA, indicating these genes potentially contribute to OA-induced behavioural changes.

The GABA hypothesis is the predominant mechanistic explanation for OA-induced behavioural changes in fish [15, 77] and may also apply to marine invertebrates [10]. Pharmacological work has supported the GABA hypothesis in marine molluscs [17, 19], including in *I. pygmaeus* [18]. In the whole-body of a pteropod mollusc, a GABA_A_ receptor transcript was upregulated after OA exposure [32]. In fish nervous tissue, OA exposure has variable effects on GABA_A_ R subunit transcript expression, causing upregulation in some species [26, 30] but not another [31]. Furthermore, differences in OA exposure duration [25, 26, 28] and magnitude [29] are associated with variable effects on the expression of genes coding for GABA_A_ R subunits within species. In *I. pygmaeus*, we found small, coordinated downregulation in the CNS and upregulation in the eyes of genes for ion-channel receptors, which included GABA_A_ receptor subunit transcripts. In the CNS, there was also upregulation of *syvn1-b* (implicated in GABA_A_α1 receptor subunit degradation [78, 79]), regulators of GABAergic neurotransmission were positively correlated with CO_2_ treatment and behaviours (*phf24*, *rac1*), and *aldh5a1* (involved in the final degradation step of GABA [64]) was negatively correlated with CO_2_ treatment and behaviours. Together, this data suggests an effect of OA on GABAergic signalling may be widespread, occurring not only in fish but also marine molluscs, however, effects may be species and tissue-specific.

Recent research suggests that various other types of neurotransmission may also be affected by OA and potentially contribute to consequent behavioural responses. Pharmacological research has identified altered function of a range of different types of ligand-gated Cl^−^ channels in *I. pygmaeus* [18] and the dopamine D1 receptor in a damselfish [80] contributing to OA-induced behavioural changes. Elevated CO_2_ upregulated glycinergic, cholinergic and glutamatergic transcripts in the whole body of a pteropod mollusc [32], upregulated glutamatergic transcripts in the non-nervous tissue of oysters [81, 82], and altered acetylcholine receptor transcript expression in the whole body of another pteropod mollusc [33]. In fish nervous tissue, exposure to OA conditions caused upregulation of genes coding for glutamatergic and cholinergic neurotransmission of some species [26, 30, 31], but downregulation in others [21, 28]. In *I. pygmaeus*, we identified small, coordinated downregulation in the CNS and upregulation in the eyes of genes involved in neurotransmission mediated by ligand-gated ion channels, including transcripts for subunits of ionotropic glutamate, glycine and acetylcholine receptors. There was also small, coordinated downregulation in the CNS of G protein-coupled receptor (GPCR)-mediated neurotransmission, including genes for subunits of metabotropic glutamate, serotonin, dopamine and GABA (GABA_B_) receptors. Key genes for glutamatergic and monoaminergic signalling were DE, and a subunit of nicotinic acetylcholine receptors (*chrna10*) was correlated with CO_2_ treatment and behaviours in both tissues. Overall, this suggests that OA not only impacts GABA_A_ R function, but various different types of neurotransmission mediated by ligand-gated ion channels and GPCRs. However, as with GABAergic signalling, OA effects on other types of neurotransmission may vary by species and tissue type, and possibly other factors such as the magnitude and duration of OA exposure. Further experimentation is needed to understand how OA-induced transcriptomic responses translate to neurotransmission function and behaviour.

Genes involved in the general processes required for synaptic neurotransmission were attenuated in the CNS of *I. pygmaeus* after OA exposure, including Ca^2+^, K^+^ and Na^+^ ion channels required for maintenance of membrane potential, action potential generation and neurotransmitter release. There was also a negative correlation between the expression of genes regulating synaptic neurotransmission with CO_2_ treatment and activity traits. Genes for Ca^2+^ and K^+^ transporters and the regulation of neurotransmitter release were also downregulated in the brain of spiny damselfish collected from CO_2_ seeps [28], but upregulated after acute and developmental OA exposure [26], and in fish olfactory tissue after short-term [31] and transgenerational OA exposure [30]. These transcriptomic signatures suggest an even more widespread effect of OA on neurotransmission, potentially altering the general processes required for synaptic neurotransmission to occur. However, experimentation is required to determine functional effects.

Neuroplasticity is the ability of the nervous system to change. Both neurogenesis (the process by which new neurons are generated and integrated into existing neural circuits) and synaptic plasticity (changing of synaptic strength over time) contribute to neuroplasticity [83]. In the CNS of *I. pygmaeus* we found small, coordinated upregulation and positive correlation with CO_2_ treatment and activity traits of genes involved in all the stages required for neurogenesis (re-entering and exiting the cell cycle, cell proliferation and differentiation to form new neurons, and neural wiring involving cell migration and adhesion for new neurons to be incorporated into existing circuits). Widespread upregulation and positive correlations with CO_2_ treatment and behaviours of genes in the CNS of *I. pygmaeus* involved in transcription, RNA processing, and protein processing could potentially be a response to deal with the changed protein demand required due to increased neuroplasticity. Notably, *cdk10*, which plays an important role in neurogenesis [84] was identified as correlated with the OA-induced increase in exploratory interactions in both the CNS and eyes. Genes involved in synaptogenesis (the formation of new synapses) and synaptic plasticity were also positively correlated with CO_2_ treatment and OA-affected behaviours in the CNS of *I. pygmaeus*.

Despite not assessing the nervous tissue specifically, transcripts involved in neuronal cell adhesion, neuronal differentiation and survival and synaptic plasticity were also upregulated in the whole body of a pteropod mollusc after OA exposure [32]. In fish nervous tissue, genes involved in neurogenesis were upregulated in some species but not others [22], and genes involved in synaptic plasticity were upregulated in some species [26, 30], but downregulated in another species [21]. Thus, OA-induced transcriptomic responses related to neuroplasticity may be widespread, occurring in fish and marine molluscs. However, this response may be taxa-specific and/or could be affected by differences in CO_2_ exposure duration and magnitude that have differed between studies.

Elevated CO_2_ alters the molluscan immune response, with most research focusing on bivalves [85–89]), though the immune response of an octopus was also affected by elevated CO_2_ [90]. Here, we found DE of genes that regulate immune signal transduction pathways and which are also implicated in the molluscan immune response (*map4k5/3, syvn1-b, psenen*, *cbs*) [73, 91–93]. There were also changes in expression of a range of genes that code for immune effectors, including iron sequestration (*tf* and *cbs*), autophagy (*map1l3ca/b*), controlling the pool of available nucleoside triphosphates (*nme6*), and phagocytosis (‘cell adhesion’ and multiple cytoskeleton functional categories). Previous research has also indicated altered phagocytosis in molluscs at elevated CO_2_; adhesion capacity of haemocytes was decreased in a clam and expression of integrin (involved in cell adhesion for phagocytosis) was decreased in an oyster species [94] and increased in another oyster [81]. Furthermore, the phagocytic rate and cytoskeleton component abundance was decreased, and the expression of cytoskeleton genes was upregulated, in a clam at elevated CO_2_ [87]. Our results show an effect of OA on the transcriptional profile of genes implicated in the immune response suggesting OA-induced alterations in immune function may also occur in molluscan nervous tissue, though further research directly measuring immune function within the nervous system is required.

Cross-talk between the neuroendocrine and immune systems coordinates appropriate physiological and behavioural responses to environmental change [95]. In molluscs, neuronal release of norepinephrine regulates immune responses through a neuroendocrine-immune axis-like pathway [96]. Specifically, changes in the expression and activity of *maoa* (upregulated in *I. pygmaeus* eyes here) plays a key role in immune functioning via norepinephrine in molluscs [66–68]. Immune-derived factors can also feedback to alter the nervous system and behaviour [97, 98]. Indeed, *tf*, which is a key component of the molluscan innate immune response (Lambert et al., 2005; Ong et al., 2006; Herath et al., 2015; Salazar et al., 2015; Li et al., 2019) was upregulated and positively correlated with CO_2_ treatment and activity traits in the CNS of *I. pygmaeus*. We also found the expression of genes coding for integrins *itga4 and itga9*, cell adhesion molecules playing a key role in invertebrate immune responses (Johansson, 1999; Terahara et al., 2006), were positively correlated in the CNS with CO_2_ treatment and activity traits. Therefore, it is possible that OA-induced changes in neurotransmission could have consequences on immune function, and changes in immune function could also feedback on the nervous system to alter behaviours at elevated CO_2_. However, the potential links between OA, neurotransmission, immune function and behaviour remain to be experimentally tested.

Oxidative stress occurs when there is an imbalance between the production of reactive oxygen species (ROS) and protection by antioxidant mechanisms [99]. Elevated CO_2_ induces oxidative stress in molluscs, increasing ROS and altering antioxidant defences inducing DNA damage, lipid peroxidation and apoptosis [100–105]. In *I. pygmaeus*, we found DE of genes implicated in the production of antioxidants, including ascorbate and glutathione. We also identified upregulation in the CNS of genes involved in DNA damage and repair, protein damage and endoplasmic reticulum stress, and cellular stress-induced apoptosis. In molluscs, OA exposure has previously been shown to result in DNA damage [100, 106] and increased apoptosis [100, 104].

The nervous system is particularly vulnerable to oxidative stress [107, 108] and oxidative stress-induced damage within the nervous system can disrupt neurotransmission and neuronal function [99, 108–110]. In mammals, a link between oxidative stress in the nervous system and changes in behaviour has been demonstrated [111–113]. In the CNS of *I. pygmaeus*, we identified a positive correlation between the expression of genes implicated in oxidative stress and CO_2_ treatment and OA-affected behaviours. In the eyes, two genes (*crb* and *zranb1*) potentially correlated with OA-induced behavioural alterations of *I. pygmaeus* are implicated in oxidative-stress induced retinal degeneration [114, 115]. In particular, *crb* prevents photoreceptor degeneration by limiting the production of ROS and the resultant oxidative damage [114]. A recent study in a cuttlefish found the behavioural effects of OA were associated with an altered retinal structure and an increase in apoptotic cells within the eyes [116]. Thus, it’s possible that OA-induced oxidative stress could contribute to behavioural alterations at elevated CO_2_, potentially through central and peripheral mechanisms, but further electrophysiological and whole-animal behavioural experimentation is required.

When interpreting our results, there are a few important things to consider. Firstly, despite the reasonable assumption that changes in gene expression driving behavioural responses occur prior to behavioural production [117], we measured gene expression immediately after the OA-induced behavioural responses in *I. pygmaeus* due to the necessity of terminal sampling to obtain nervous tissue. Furthermore, the process of transcribing genes is far too slow to mediate rapid behavioural responses, which are instead mediated by fast electrical signals passed along and between neurons [117]. Thus, our transcriptomic results do not describe the neuronal mechanisms driving the immediate behavioural responses to a stimulus, but rather those that likely contribute to longer-term changes in behaviour [117, 118] in OA conditions. Secondly, organism responses to OA can be sex-specific [119], including marine invertebrate behavioural responses [120, 121]. We used males only in this study. Future research could consider using both sexes to determine whether behavioural responses to OA are sex-specific, and if so whether differing transcriptional profiles underly these sex-specific responses.

## Conclusion

Here, we demonstrate differential expression of specific genes and widespread small but coordinated changes in expression of genes belonging to relevant functional categories in the CNS and eyes following short-term exposure of male *I. pygmaeus* to OA. We also report genes correlated with both CO_2_ treatment and OA-affected behaviours, indicating these genes as potentially correlated with CO_2_-induced behavioural change in *I. pygmaeus*. The results identify alterations in the transcriptional profile of genes implicated in neurotransmission, neuroplasticity, immune function and oxidative stress. These molecular changes may contribute to OA-induced behavioural change, as suggested by correlations between gene expression profiles, CO_2_ treatment and OA-affected behaviours. Our results build on existing knowledge and provide novel hypotheses for future experiments, including electrophysiological and behavioural tests, to determine the range of processes responsible for behavioural changes in marine animals exposed to projected future OA conditions.

### Electronic supplementary material

Below is the link to the electronic supplementary material.

Supplementary Material 1

Supplementary Material 2

Supplementary Material 3

Supplementary Material 4

## Data Availability

The datasets supporting the conclusions of this article are available in the National Centre for Biotechnology Information (NCBI) and Research Data JCU repositories. Raw RNA-sequencing and ISO-sequencing data, and the transcriptome assembly (fasta file) can be found at NCBI BioProject PRJNA798187. Raw gene count data, raw water sampling data, all scripts used for bioinformatic analyses, the annotated transcriptome assembly (OmicsBox and csv files), R code used for the statistical analyses (differential expression and gene set analyses), and data files to accompany the statistical analyses are available from the Research Data JCU repository at DOI 10.25903/ha66-mm11. All R code for the statistical analyses (correlating gene expression profiles with CO2 treatment and OA-affected behaviours), as well as accompanying data files for the statistical analyses, can be found at the Research Data JCU repository at DOI 10.25903/7dcz-th66.
