# Supplementary material for: Transcriptomic responses in the nervous system and correlated behavioural changes of a cephalopod exposed to ocean acidification
Source: BMC Genomics. 2024 Jun 25;25:635. doi: 10.1186/s12864-024-10542-5 (PMC11202396; doi:10.1186/s12864-024-10542-5)
Supplement: Supplementary file 1 — Supplementary Material 1 [file 12864_2024_10542_MOESM1_ESM.pdf]

Supplemental Information for:

Transcriptomic responses in the nervous system and correlated behavioural changes of a cephalopod exposed to ocean acidification

Jodi T. Thomas, Roger Huerlimann, Celia Schunter, Sue-Ann Watson, Philip L. Munday, Timothy Ravasi

Table of Contents:

**Supplementary Text S1. Water sampling methods.**	Page 3
**Supplementary Text S2. *de novo* transcriptome assembly.**	Page 4
**Supplementary Text S3. Correlating gene expression profiles with CO_2 treatment and OA-affected behaviours.**	Page 6
**Figure S1. Diel CO2 variation at the site *I. pygmaeus* were collected.**	Page 8
**Figure S2. Detailed workflow from tissue sampling to bioinformatics analyses and statistical analyses.**	Page 9
**Figure S3. Sample dendrograms to detect sample outliers.**	Page 10
**Figure S4. Choosing and checking the soft threshold power for network construction using the CNS samples.**	Page 11
**Figure S5. Choosing and checking the soft threshold power for network construction using the eyes samples.**	Page 12
**Figure S6. Cluster dendrogram with assigned co-expression network modules before and after merging modules.**	Page 13
**Figure S7. Canonical loadings and cross loadings for each canonical function (CF) of each variable in the CNS.**	Page 14
**Figure S8. Canonical loadings and cross loadings for each canonical function (CF) of each variable in the eyes.**	Page 16
**Figure S9. Canonical correlation analysis biplot for the CNS canonical functions 1 and 2.**	Page 18
**Figure S10. Canonical correlation analysis biplot for the CNS canonical functions 1 and 3.**	Page 19
**Figure S11. Canonical correlation analysis biplot for the CNS canonical functions 2 and 3.**	Page 20
**Figure S12. Canonical correlation analysis biplot for the CNS canonical functions 1 and 4.**	Page 21
**Figure S13. Canonical correlation analysis biplot for the CNS canonical functions 2 and 4.**	Page 22
**Figure S14. Canonical correlation analysis biplot for the CNS canonical functions 3 and 4.**	Page 23
**Figure S15. Canonical correlation analysis biplot for the eyes canonical functions 1 and 2.**	Page 24

**Figure S16. Canonical correlation analysis biplot for the eyes canonical functions 1 and 3.**	Page 25
**Figure S17. Canonical correlation analysis biplot for the eyes canonical functions 2 and 3.**	Page 26
**Figure S18. Canonical correlation analysis biplot for the eyes canonical functions 1 and 4.**	Page 27
**Figure S19. Canonical correlation analysis biplot for the eyes canonical functions 2 and 4.**	Page 28
**Figure S20. Canonical correlation analysis biplot for the eyes canonical functions 3 and 4.**	Page 29
**Figure S21. Final modules of interest in the A) CNS and B) eyes.**	Page 30
**Figure S22. Species distribution for the top blast hits of the annotated transcriptome assembly.**	Page 31
**Figure S23. Heatmap of all samples showing the expression pattern of all genes in the CNS and eyes.**	Page 32
**Figure S24. Volcano plots showing all genes in the A) CNS and B) eyes.**	Page 33
**Figure S25. Dotplot showing the results from gene set enrichment analysis (GSEA) using GO terms/functional categories in the CNS and eyes.**	Page 34
**Supplementary Tables**	Page 35
**File S1. TapeStation electropherograms for each of the 40 RNA samples used for RNA-sequencing.**	Page 37
**References**	Page 38

Supplementary Text S1. Water sampling methods.

To evaluate the magnitude of natural diel CO_2 fluctuations and the ecological relevance of our experimental CO_2 treatment levels, water samples were taken from the same location where two-toned pygmy squid (*Idiosepius pygmaeus*) were collected. *I. pygmaeus* were collected from August - October 2019, and water samples from August - September 2021, from coastal waters around the Townsville breakwater complex. All water samples were collected with 250 mL borosilicate glass bottles. Bottles were dipped into the water upside down and at approximately 25 cm deep the bottle was inverted several times to allow water to enter and remove all air bubbles, and the lid was screwed on underwater. Each sample was taken in pairs; one was placed directly in the dark for storage until lab measurements, and the other was used immediately for measurements of water temperature (Comark C26, Norfolk, UK) and pH_{NBS} (Seven2Go™ pro Conductivity Meter with an InLab Expert Go-ISM pH electrode, Metler Toledo). Three pairs of water samples were taken in immediate succession at each location and sampling time. All lab measurements were taken within 2.13 ± 1 hour (mean ± SD) of water sample collection. Total alkalinity was measured by Gran titration (888 Titrando, Metrohm AG, Switzerland) and salinity was measured with a conductivity sensor (HQ40d, Hach, Loveland, CO, USA). CO_2 values were calculated in CO2SYS v.2.1 (https://cdiac.ess-dive.lbl.gov/ftp/co2sys/CO2SYS_calc_XLS_ v2.1/) using the constants K1, K2 from Mehrbach et al. (1973) and refit by Dickson and Millero (1987) Dickson and $KHSO_4$ from Dickson et al. (2007).

To determine any spatial variation within the breakwater marina complex, three pairs of water samples were taken from each of three different locations (19°15'06.3"S 146°49'22.4"E; 19°15'08.1"S 146°49'27.6"E; 19°15'11.8"S 146°49'21.6"E), both before first light (approximately 5:30) and mid-afternoon (approximately 13:30). CO_2 levels were consistent across these three locations, therefore all subsequent sampling was done from one location (19°15'06.3"S 146°49'22.4"E). To determine the best time for afternoon sampling to capture maximum change in CO_2 levels, three pairs of water samples were collected at each of three time points; 12:30, 13:30 and 14:30. These time points were chosen based on previous research that found minimum CO_2 was reached between 12:30 and 14:20 at Lizard Island, Great Barrier Reef (Hannan et al., 2020). CO_2 levels were consistent across these three time points, therefore all subsequent sampling was done at 13:30. After these initial checks were completed, water sampling was carried out to determine any diel CO_2 variation. Three pairs of water samples were taken before first light (approximately 5:00) and at 13:30 across five days of differing tidal heights, all from the same location. Figure S1 shows the diel CO_2 variation. All raw data from water sampling can be found at DOI 10.25903/ha66-mm11 (this DOI is embargoed until peer-reviewed publication).

Supplementary Text S2. *de novo* transcriptome assembly

ISO-sequencing

Library preparation and sequencing was carried out by the Sequencing Section, Okinawa Institute of Science and Technology Graduate University, Japan on four samples that were also used for RNA-seq, one of each tissue type and CO_2 level. RNA from the eyes was purified with oligo d(T) beads due to carry over of pigmentation (NEBNext® Poly(A) mRNA Magnetic Isolation Module, New England BioLabs Inc.). RNA was quantified by Qubit 4 Fluorometer (Qubit RNA HS Assay Kit, Life Technologies). One library was prepared for each sample following the ISO-Seq™ Express Template Preparation for Sequel® and Sequel II Systems protocol with standard size selection (86 µL ProNex® Beads). Libraries were sequenced on one SMRTcell of a PacBio Sequel II.

De novo transcriptome assembly

The ISO-seq data was processed using the PacBio isoseq3 pipeline. The raw subreads were compiled into circular consensus sequence (ccs) reads by ccs (v4.2.0) with the minimum number of full passes set at three and the minimum predicted accuracy of a read at 0.9. Lima (v1.11.0) was used to classify the ccs reads as full-length (FL) (by the presence of both 5' and 3' primers) and remove index sequences with '--peek-guess'. The resulting FL reads from each tissue/barcode were combined and isoseq3 refine (v3.3.0) was used to remove concatemers and polyA tails, producing full-length non-concatemer (FLNC) reads. The FLNC reads were then clustered by isoform using isoseq3 cluster (v3.3.0), using the ccs quality values ('--use-qvs') to obtain a consensus sequence for each isoform. Redundancy removal was performed using CD-HIT-EST (v4.6) (Fu et al., 2012; Li and Godzik, 2006) to collapse contigs with at least 99% identity. TransDecoder (v5.5.0) (Brian and Papanicolaou, n.d.) was used to identify candidate coding regions/open reading frames (ORFs). The single best ORF per contig was chosen based on blast homology to known proteins in the NCBI nr database subset for mollusca (nr_mollusca, downloaded 01/2021) using BLASTp from BLAST+ (v2.10.0+) with max_target_seqs 1 and an e-value cut-off of 1^{-5}, and then based on ORF length (minimum 100 amino acids). The entire transcript was retained for each identified ORF.

The quality and completeness of the transcriptome was assessed before and after redundancy removal, and for the final transcriptome assembly (after ORF identification by TransDecoder). Quality was assessed using Transrate (v1.0.3) (Smith-Unna et al., 2016) and completeness using Benchmarking Universal Single-Copy Orthologs (BUSCO v4.1.2) (Manni et al., 2021), using the lineage mollusca_odb10.2019-11-20. Quality and completeness were also assessed by blasting the transcriptome against nr_mollusca (e-value cut-off = 1^{-5}, '-max_target_seqs 1', BLASTx from BLAST+ (v2.10.0+) (Camacho et al., 2009)), and mapping the trimmed, decontaminated RNA-seq reads to the transcriptome assembly (local alignment, Bowtie2 (v2.4.1) (Langmead and Salzberg, 2012)).

Transcriptome annotation

The transcriptome was blasted against the entire NCBI nr database (downloaded 01/2021) using BLASTx from BLAST+ (v2.10.0+) (Camacho et al., 2009) with an e-value cut-off of 1^{-5}, outfmt 14, and '-num-alignments' and '-max_hsps' both set at 20. Functional annotation was carried out in OmicsBox (v1.4.12) (BioBam Bioinformatics, 2019) using BLAST2GO mapping (Goa version 2020.10, all default settings) (Götz et al., 2008), followed by BLAST2GO annotation (all default settings) (Götz et al., 2008) and InterProScan (v5.50-84.0, all default settings) (Jones et al., 2014). The InterProScan GOs were then merged with the annotations.

Supplementary Text S3. Correlating gene expression profiles with CO_2 treatment and OA-affected behaviours.

Gene co-expression network construction and module detection

To analyse the correlation between gene expression and behavioural traits of squid across CO_2 treatments, we employed weighted gene co-expression network analysis (WGCNA) followed by canonical correlation analysis (CCA) on the CNS and eyes, separately (Figure 1). The gene-level counts for all 20 samples from each tissue were normalised, transcripts with low read counts were removed (≤10 counts in ≥ 90% samples) and the remaining count data was variance stabilised in DESeq2 (v1.30.1) (Love et al., 2014). This count data was then used in the WGCNA package (v1.70-3) (Langfelder and Horvath, 2008) for co-expression network construction and module detection. No genes were identified as outliers, using 'goodSamplesGenes'. To detect any outliers among the samples themselves, a sample dendrogram was created using hierarchical clustering with the 'average' method. Three and two obvious sample outliers were identified and removed from the analysis in the CNS and eyes, respectively (Figure S3). Soft thresholding power was evaluated and powers of 14 and 13 were chosen for the CNS and eyes, respectively, to approximate a scale free topology (Figures S4 and S5). The following co-expression network construction and module detection steps were carried out on a high-performance computing cluster at Okinawa Institute of Science and Technology, Japan to allow multiple threads for a full network analysis occurring in one block. A signed correlation network adjacency was calculated using Pearson correlation and the chosen soft thresholding power. The adjacency was transformed into a signed topological overlap matrix (TOM) and the corresponding dissimilarity was calculated (1-TOM). A cluster dendrogram of genes was created using hierarchical clustering with the 'average' method and the dissimilarity TOM. Modules were detected using dynamic tree cut with the hybrid method, a minimum cluster size of 30, an intermediate sensitivity to cluster splitting (deepSplit = 2) and the Partitioning Around Medoids (PAM)-like step set to not respect the dendrogram (pamRespectsDendro = FALSE). Modules with a correlation of ≥ 0.70 were then merged (Figure S6, Tables S1 and S2). Eigengenes were calculated for each final module, which is the first principal component in the corresponding module used to represent the gene expression profiles of that module. These module eigengenes (MEs) allow gene modules to be correlated with external traits (Langfelder and Horvath, 2008).

Module eigengene correlation with behavioural traits

Canonical correlation analysis (CCA) using package CCA (v1.2.1) (González et al., 2008) was used to explore the correlations between the two sets of variables from the same individual squid: ME set = MEs from each module; traits set = CO_2 level (current-day or elevated) and behavioural traits (active time (s), distance (cm), speed (cm/s), time in Zone A (s), whether the squid displayed an exploratory/aggressive interaction (yes/no), number of exploratory/aggressive interactions). The canonical loadings and cross-loadings were calculated for the first four canonical functions (CF) in the CNS and eyes, separately. The first four CFs were chosen for interpretation as they explained a substantial amount of variance between the traits set and MEs set (canonical correlation > 0.6) (Sherry and Henson, 2005). Heatmaps of all canonical loadings and cross-loadings for all variables of each set were created for each of the four CFs (Figures S7 and S8). If

the canonical loading and cross-loading of a given variable from both the ME set and traits set were ≥ 0.3 for the same CF, this ME and trait were considered correlated. A cut-off value of 0.3 is commonly used (Kabir et al., 2014; Lambert and Durand, 1975) and was a clear cut-off for this dataset (Figures S7 and S8). Biplots were created for each two-way combination of the four CFs (Figures S9 – S20). MEs and traits within the same or opposite quarters of the biplot, and sitting on or outside the biplot inner ring with a radius of 0.5, were considered positively or negatively correlated, respectively. If MEs and traits were considered correlated by both the canonical loadings/cross-loadings heatmap and biplots they were identified as modules of interest for the given trait(s).

Module membership vs gene significance

The Pearson correlation of module membership (MM, higher value indicates the gene is more highly connected to the given module) and gene significance (GS, higher value indicates a more biologically relevant gene) was used to check the modules of interest identified by CCA (Langfelder and Horvath, 2008). A correlation of GS and MM imply that genes more highly connected with a given module also tend to be more highly correlated with the given trait, providing another measure for the importance of this module with the given trait (Langfelder and Horvath, 2008). All modules of interest initially identified by CCA that had a MM vs GS correlation (R-value) > 0.2, a commonly used threshold for evidence of a weak correlation (Evans, 1996), were chosen as the final modules of interest (Figure S21).

Identification of hub genes

Within the final modules of interest, the MM and GS values of each gene were used as a screening method to identify biologically relevant, highly interconnected hub genes (Fuller et al., 2007; Horvath and Dong, 2008; Langfelder and Horvath, 2008), i.e. to find genes correlated with CO_2 treatment and each behavioural trait. Hub genes were defined as those genes within the final modules of interest with a very strong correlation with the module (MM > 0.8) and a moderate correlation with the given trait (GS > 0.4). As a final check of these identified hub genes, the Pearson correlation (R-value) between the normalised expression of each hub gene and the given trait was calculated and genes with a very weak correlation (R < 0.2) were excluded. This resulted in the final list of hub genes. All hub genes for CO_2 treatment were compared across tissues to identify hub genes for CO_2 treatment that are CNS-specific, eyes-specific or found in both tissues. Hub genes for CO_2 treatment that were also a hub gene for one or more behavioural traits were identified as genes correlated with the associated OA-induced behavioural change.

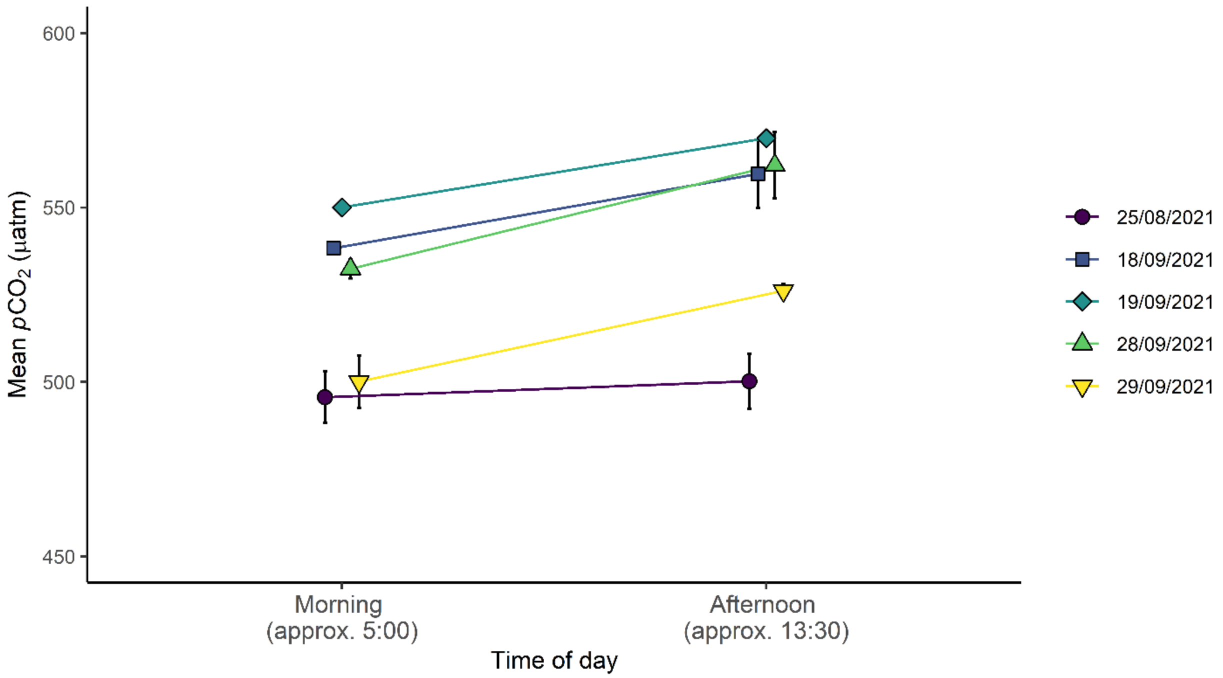

Figure S1. Diel CO_2 variation at the site *I. pygmaeus* were collected. Water samples were taken to measure CO_2 levels before first light at approximately 5:00 (morning) and at approximately 13:30 (afternoon) across five days. Points represent the mean ± standard deviation.

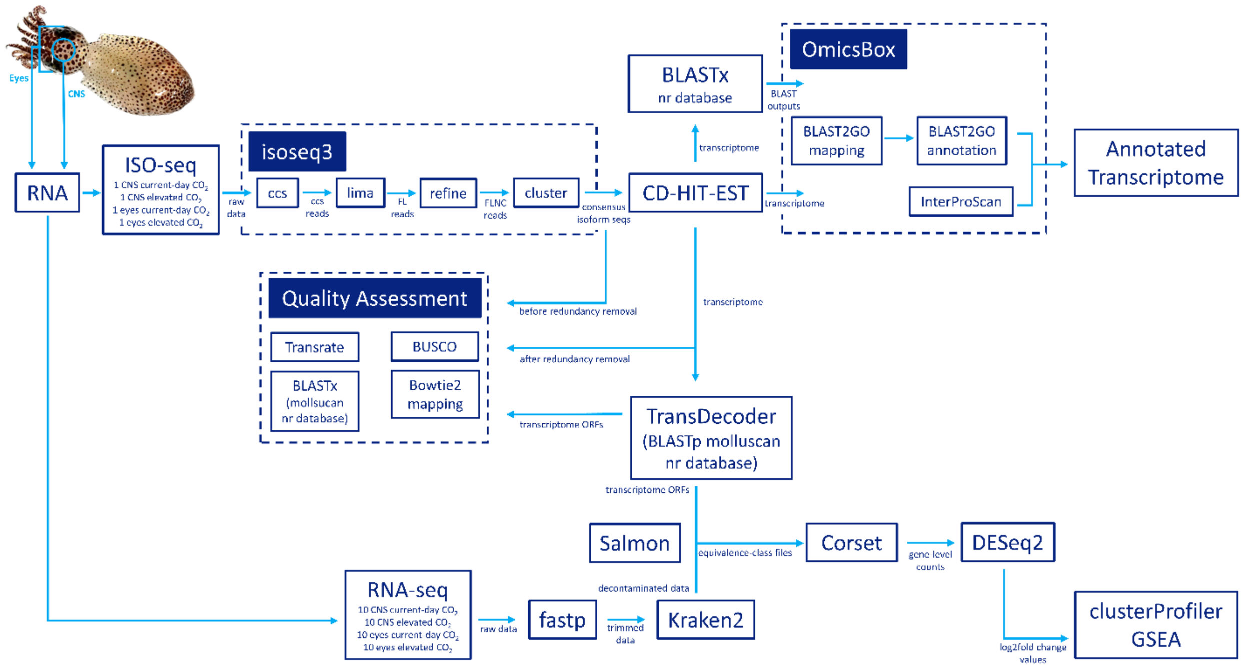

Figure S2. Detailed workflow from tissue sampling to bioinformatics analyses and statistical analyses. ccs = circular consensus sequence, DE = differentially expressed, FL = full length, FLNC = full-length non-concatemer, GSEA = gene set enrichment analysis, ISO-seq = PacBio long read ISO-sequencing, ORF = open reading frame, RNA-seq = RNA sequencing, seqs = sequences.

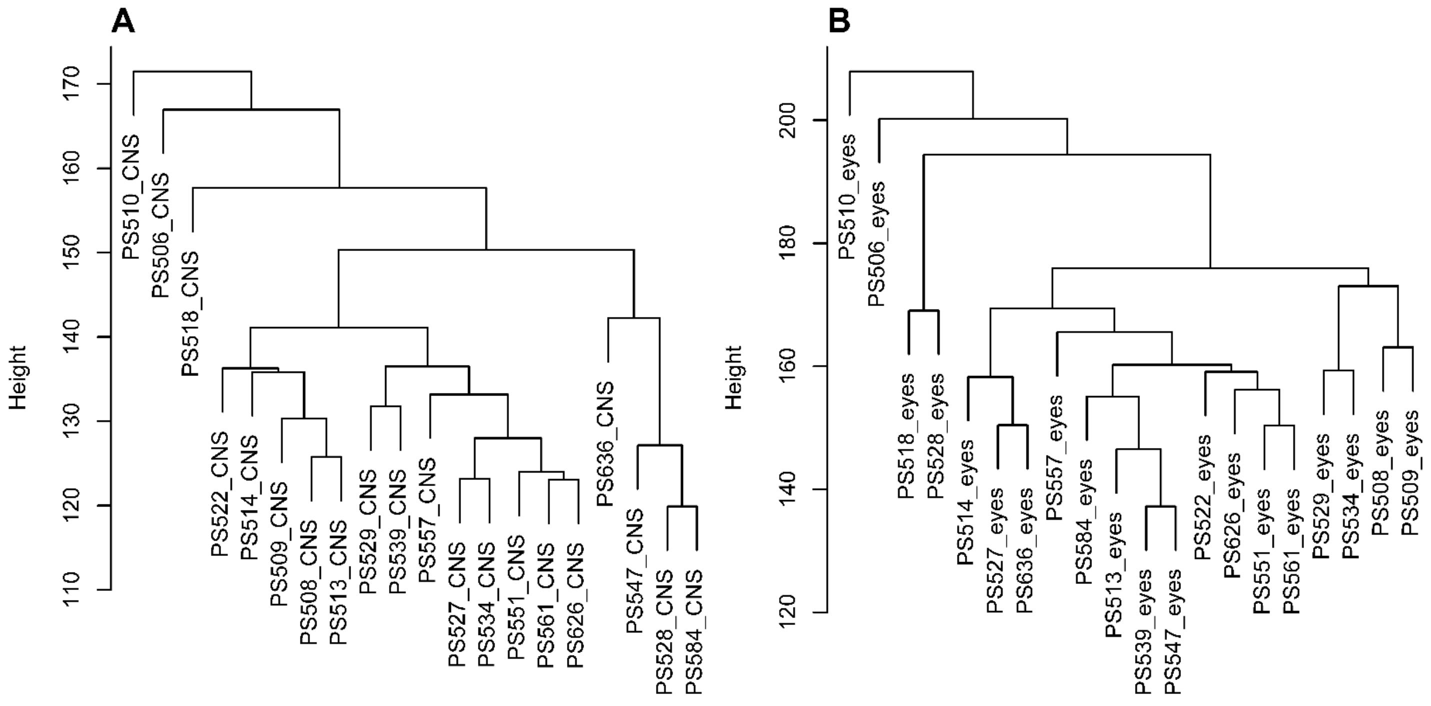

Figure S3. Sample dendrograms to detect sample outliers. Created using hierarchical clustering with the ‘average’ method. Three and two outliers were detected in the A) CNS and B) eyes, respectively. PS510_CNS, PS506_CNS, PS518_CNS, PS510_eyes and PS506_eyes were removed as they were sample outliers.

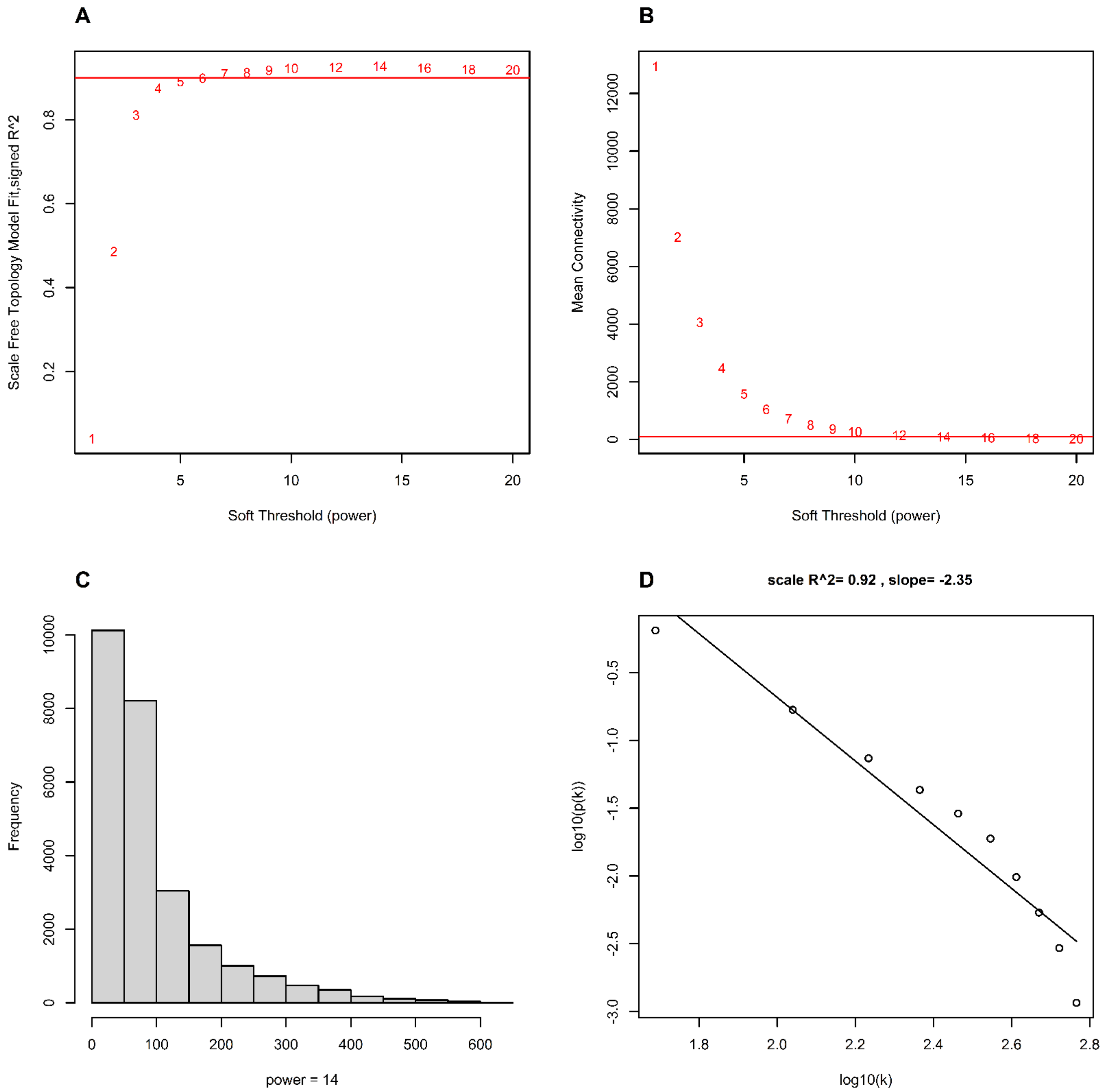

Figure S4. Choosing and checking the soft threshold power for network construction using the CNS samples. A) Scale-free topology fit index as a function of the soft-threshold power shows the network reaches approximately a scale free topology ($R^2 > 0.9$) when the soft threshold power is 7, however B) mean connectivity as a function of the soft threshold power shows mean connectivity remains high and mean connectivity only drops below 100 at a soft threshold power of 14. C) and D) were used to check the chosen soft threshold power of 14 approximates a scale free topology.

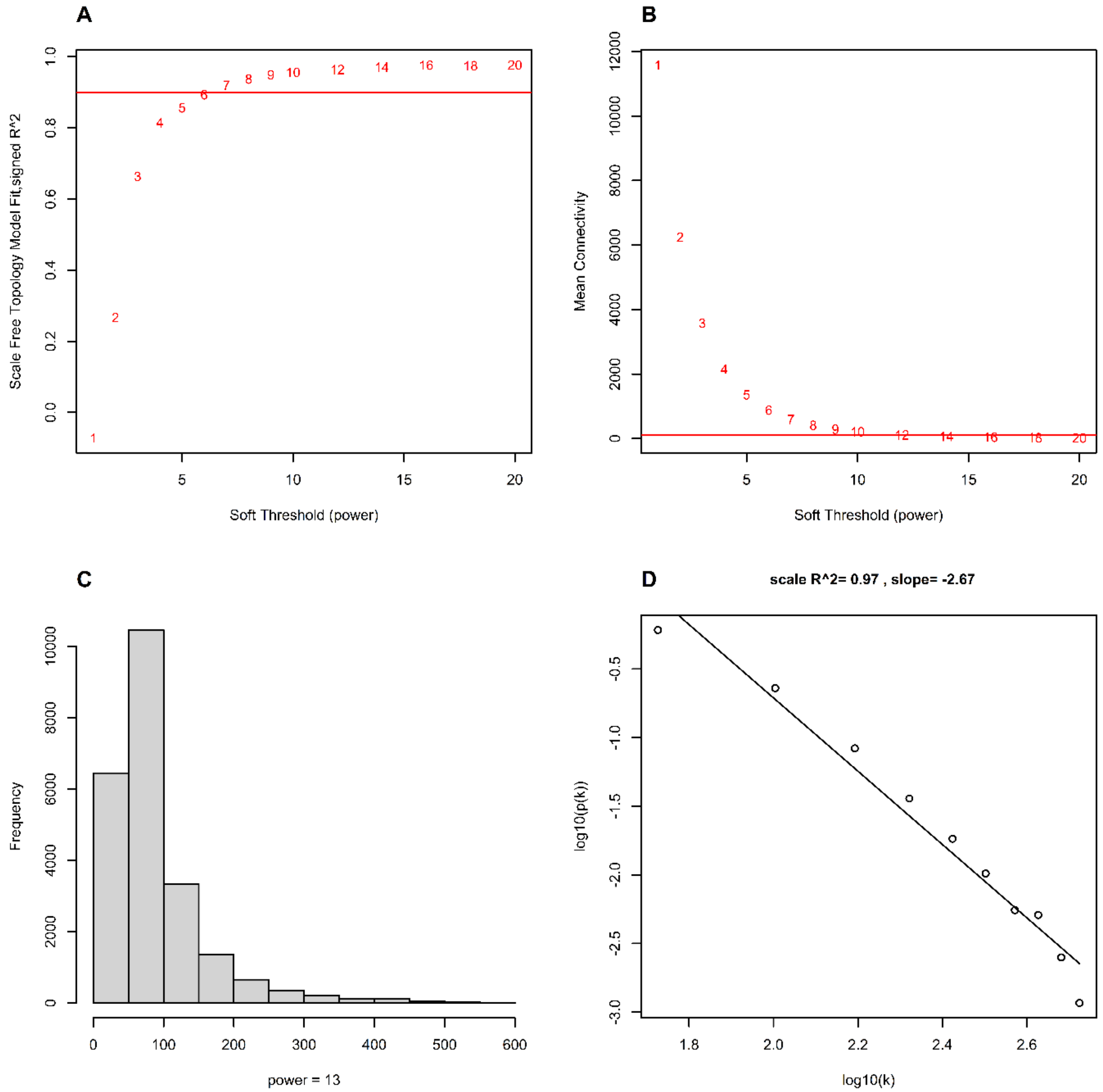

Figure S5. Choosing and checking the soft threshold power for network construction using the eyes samples. A) Scale-free topology fit index as a function of the soft-threshold power shows the network reaches approximately a scale free topology ($R^2 > 0.9$) when the soft threshold power is 7, however B) mean connectivity as a function of the soft threshold power shows mean connectivity remains high and mean connectivity only drops below 100 at a soft threshold power of 13. C) and D) were used to check the chosen soft threshold power of 13 approximates a scale free topology.

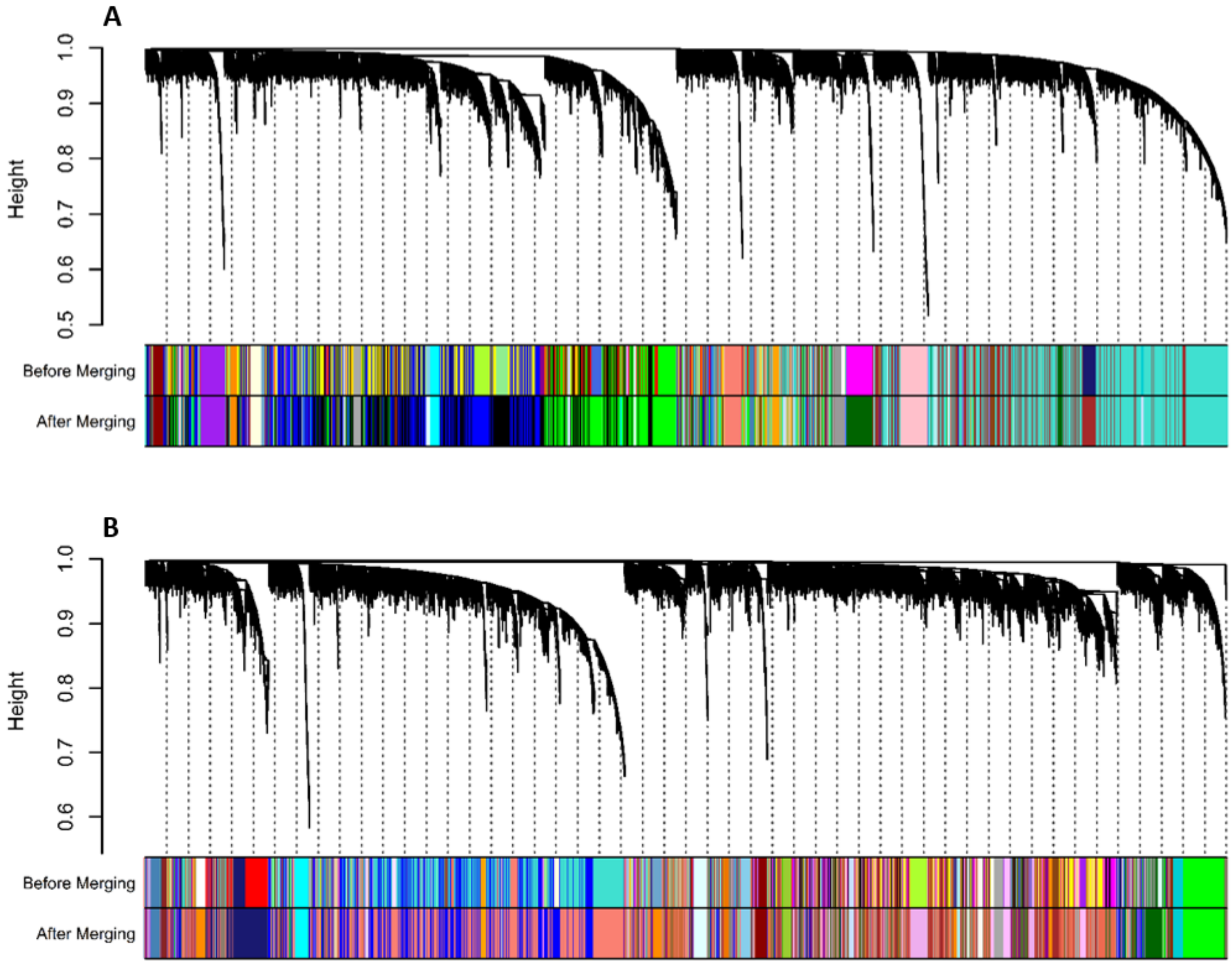

Figure S6. Cluster dendrogram with assigned co-expression network modules before and after merging modules. In the A) CNS and B) eyes. Each line in the cluster dendrogram is a gene and each module is represented by a different colour.

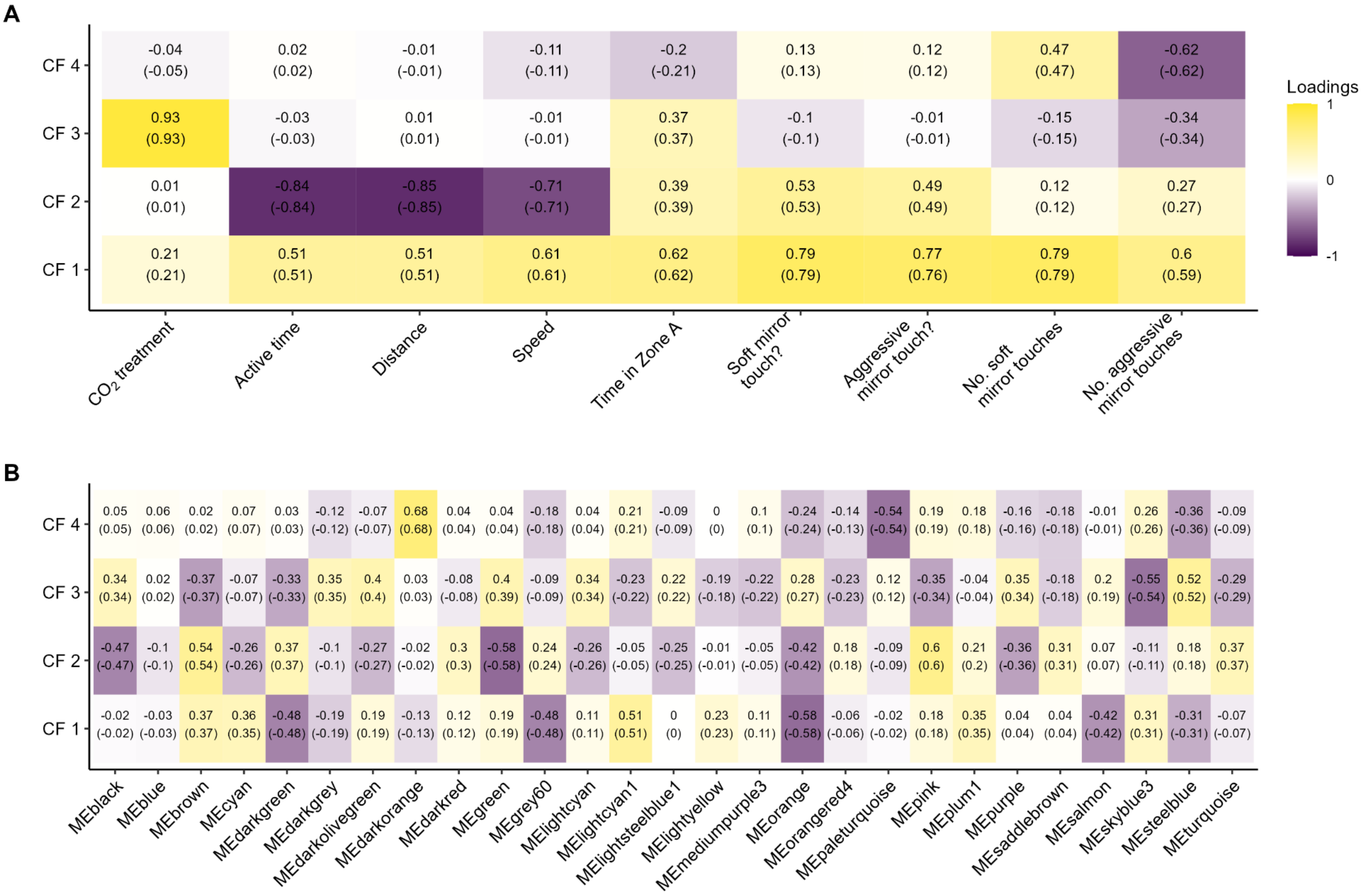
A
CF 4
CF 3
CF 2
CF 1
-0.04 (-0.05) 0.02 (0.02) -0.01 (-0.01) -0.11 (-0.11) -0.2 (-0.21) 0.13 (0.13) 0.12 (0.12) 0.47 (0.47) -0.62 (-0.62)
0.93 (0.93) -0.03 (-0.03) 0.01 (0.01) -0.01 (-0.01) 0.37 (0.37) -0.1 (-0.1) -0.01 (-0.01) -0.15 (-0.15) -0.34 (-0.34)
0.01 (0.01) -0.84 (-0.84) -0.85 (-0.85) -0.71 (-0.71) 0.39 (0.39) 0.53 (0.53) 0.49 (0.49) 0.12 (0.12) 0.27 (0.27)
0.21 (0.21) 0.51 (0.51) 0.51 (0.51) 0.61 (0.61) 0.62 (0.62) 0.79 (0.79) 0.77 (0.76) 0.79 (0.79) 0.6 (0.59)
CO_2 treatment
Active time
Distance
Speed
Time in Zone A
Soft mirror touch?
Aggressive mirror touch?
No. soft mirror touches
No. aggressive mirror touches
Loadings
1
0
-1
B
CF 4
CF 3
CF 2
CF 1
0.05 (0.05) 0.06 (0.06) 0.02 (0.02) 0.07 (0.07) 0.03 (0.03) -0.12 (-0.12) -0.07 (-0.07) 0.68 (0.68) 0.04 (0.04) 0.04 (0.04) -0.18 (-0.18) 0.04 (0.04) 0.21 (0.21) -0.09 (-0.09) 0 (0) 0.1 (0.1) -0.24 (-0.24) -0.14 (-0.13) -0.54 (-0.54) 0.19 (0.19) 0.18 (0.18) -0.16 (-0.16) -0.18 (-0.18) -0.01 (-0.01) 0.26 (0.26) -0.36 (-0.36) -0.09 (-0.09)
0.34 (0.34) 0.02 (0.02) -0.37 (-0.37) -0.07 (-0.07) -0.33 (-0.33) 0.35 (0.35) 0.4 (0.4) 0.03 (0.03) -0.08 (-0.08) 0.4 (0.39) -0.09 (-0.09) 0.34 (0.34) -0.23 (-0.22) 0.22 (0.22) -0.19 (-0.18) -0.22 (-0.22) 0.28 (0.27) -0.23 (-0.23) 0.12 (0.12) -0.35 (-0.34) -0.04 (-0.04) 0.35 (0.34) -0.18 (-0.18) 0.2 (0.19) -0.55 (-0.54) 0.52 (0.52) -0.29 (-0.29)
-0.47 (-0.47) -0.1 (-0.1) 0.54 (0.54) -0.26 (-0.26) 0.37 (0.37) -0.1 (-0.1) -0.27 (-0.27) -0.02 (-0.02) 0.3 (0.3) -0.58 (-0.58) 0.24 (0.24) -0.26 (-0.26) -0.05 (-0.05) -0.25 (-0.25) -0.01 (-0.01) -0.05 (-0.05) -0.42 (-0.42) 0.18 (0.18) -0.09 (-0.09) 0.6 (0.6) 0.21 (0.2) -0.36 (-0.36) 0.31 (0.31) 0.07 (0.07) -0.11 (-0.11) 0.18 (0.18) 0.37 (0.37)
-0.02 (-0.02) -0.03 (-0.03) 0.37 (0.37) 0.36 (0.35) -0.48 (-0.48) -0.19 (-0.19) 0.19 (0.19) -0.13 (-0.13) 0.12 (0.12) 0.19 (0.19) -0.48 (-0.48) 0.11 (0.11) 0.51 (0.51) 0 (0) 0.23 (0.23) 0.11 (0.11) -0.58 (-0.58) -0.06 (-0.06) -0.02 (-0.02) 0.18 (0.18) 0.35 (0.35) 0.04 (0.04) 0.04 (0.04) -0.42 (-0.42) 0.31 (0.31) -0.31 (-0.31) -0.07 (-0.07)
MEblack
MEblue
MEbrown
MEcyan
MEdarkgreen
MEdarkgrey
MEdarkolivegreen
MEdarkorange
MEdarkred
MEgreen
MEgrey60
MElightcyan
MElightcyan1
MElightsteelblue1
MElightyellow
MEmediumpurple3
MEorange
MEorangered4
MEpaleturquoise
MEpink
MEplum1
MEpurple
MEsaddlebrown
MEsalmon
MEskyblue3
MEsteelblue
MEturquoise

Figure S7. Canonical loadings and cross loadings for each canonical function (CF) of each variable in the CNS. In the A) traits set and B) module eigengenes (MEs) set. Canonical loadings are shown above canonical cross-loadings, which are in brackets. Colouration depicts canonical loadings.

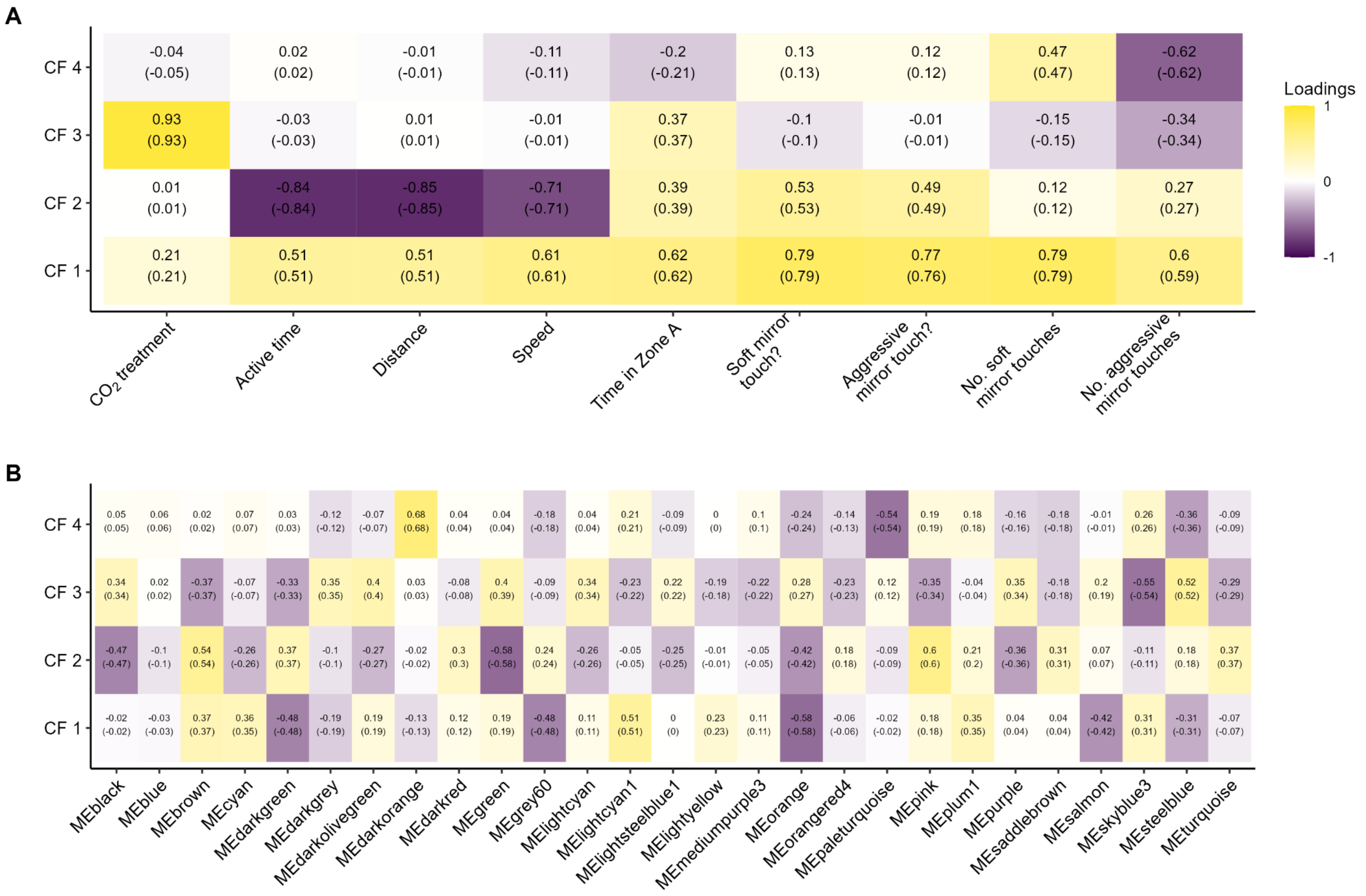
A
CF 4
CF 3
CF 2
CF 1
-0.04 (-0.05)
0.02 (0.02)
-0.01 (-0.01)
-0.11 (-0.11)
-0.2 (-0.21)
0.13 (0.13)
0.12 (0.12)
0.47 (0.47)
-0.62 (-0.62)
0.93 (0.93)
-0.03 (-0.03)
0.01 (0.01)
-0.01 (-0.01)
0.37 (0.37)
-0.1 (-0.1)
-0.01 (-0.01)
-0.15 (-0.15)
-0.34 (-0.34)
0.01 (0.01)
-0.84 (-0.84)
-0.85 (-0.85)
-0.71 (-0.71)
0.39 (0.39)
0.53 (0.53)
0.49 (0.49)
0.12 (0.12)
0.27 (0.27)
0.21 (0.21)
0.51 (0.51)
0.51 (0.51)
0.61 (0.61)
0.62 (0.62)
0.79 (0.79)
0.77 (0.76)
0.79 (0.79)
0.6 (0.59)
CO_2 treatment
Active time
Distance
Speed
Time in Zone A
Soft mirror touch?
Aggressive mirror touch?
No. soft mirror touches
No. aggressive mirror touches
Loadings
1
0
-1
B
CF 4
CF 3
CF 2
CF 1
MEblack
MEblue
MEbrown
MEcyan
MEdarkgreen
MEdarkgrey
MEdarkolivegreen
MEdarkorange
MEdarkred
MEgreen
MEgrey60
MElightcyan
MElightcyan1
MElightsteelblue1
MElightyellow
MEmediumpurple3
MEorange
MEorangered4
MEpaleturquoise
MEpink
MEplum1
MEpurple
MEsaddlebrown
MEsalmon
MEskyblue3
MEsteelblue
MEturquoise

Figure S8. Canonical loadings and cross loadings for each canonical function (CF) of each variable in the eyes. In the A) traits set and B) module eigengenes (MEs) set. Canonical loadings are shown above canonical cross-loadings, which are in brackets. Colouration depicts canonical loadings.

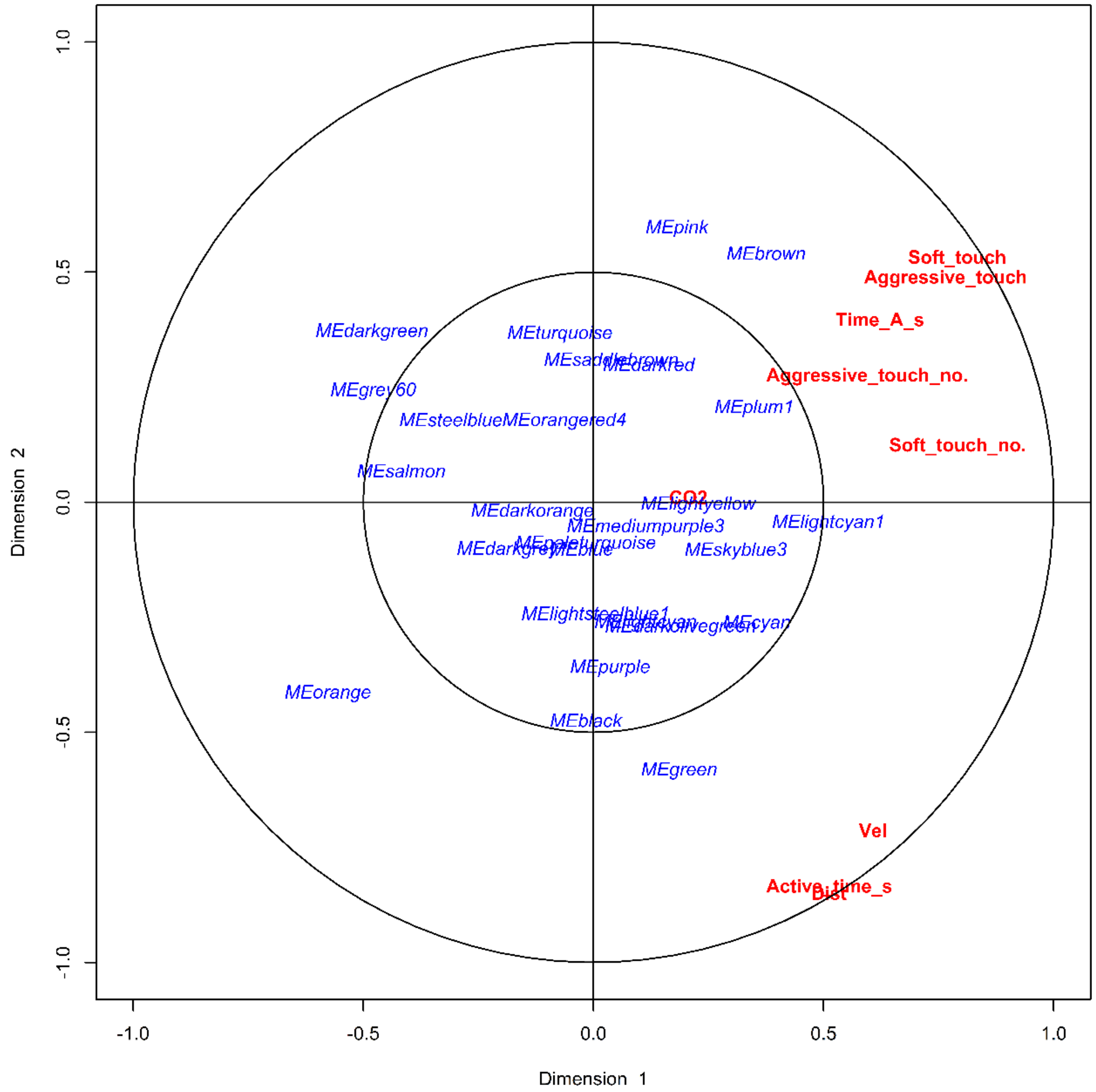

Figure S9. Canonical correlation analysis biplot for the CNS canonical functions 1 and 2. Depicting where the module eigengenes (MEs) lie in space in relation to the traits for the CNS, for canonical functions (dimensions) 1 and 2. Variables from the MEs set and the traits set are in blue and red, respectively. The inner ring is set at a radius of 0.5.

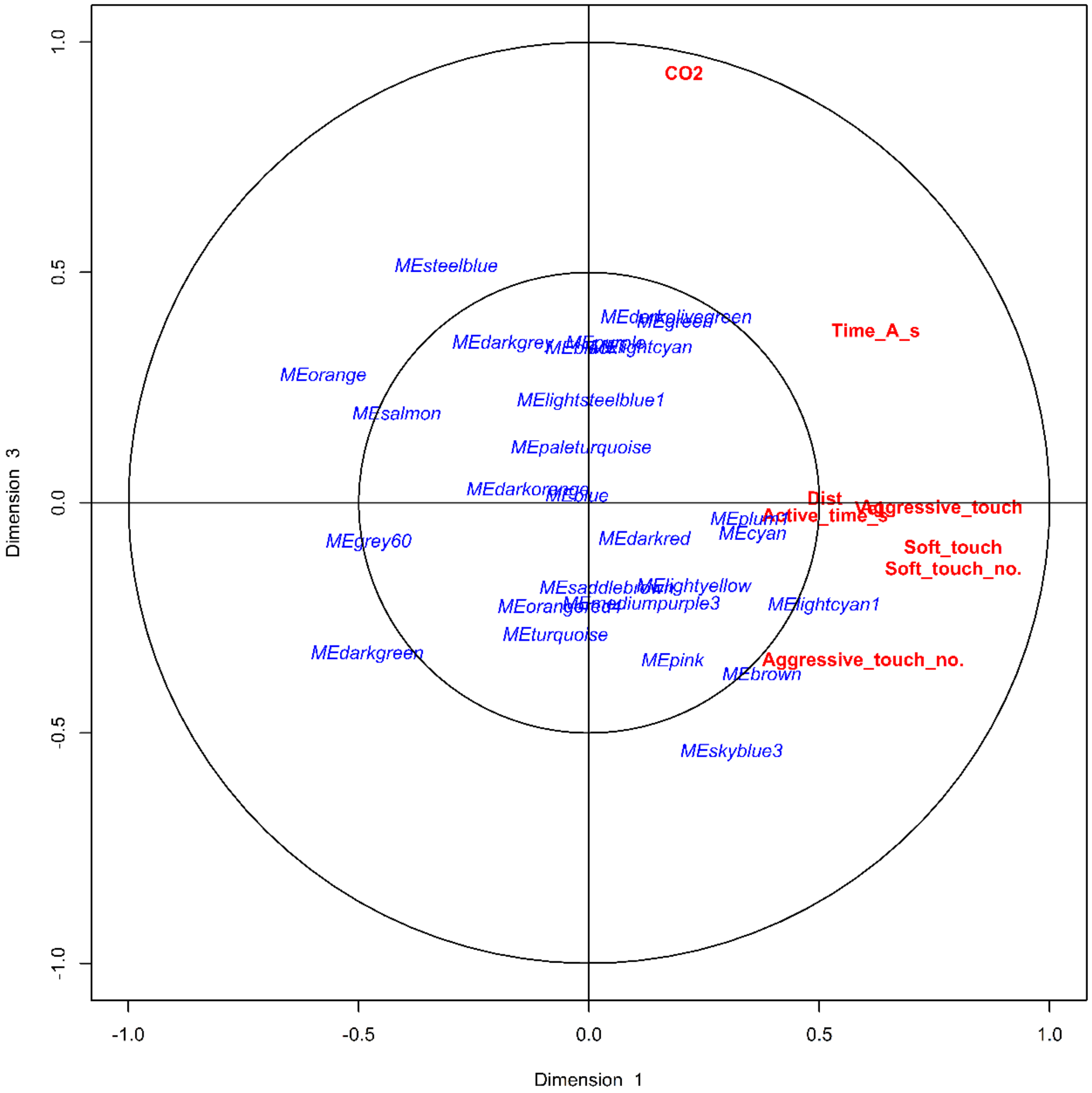

Figure S10. Canonical correlation analysis biplot for the CNS canonical functions 1 and 3. Depicting where the module eigengenes (MEs) lie in space in relation to the traits for the CNS, for canonical functions (dimensions) 1 and 3. Variables from the MEs set and the traits set are in blue and red, respectively. The inner ring is set at a radius of 0.5.

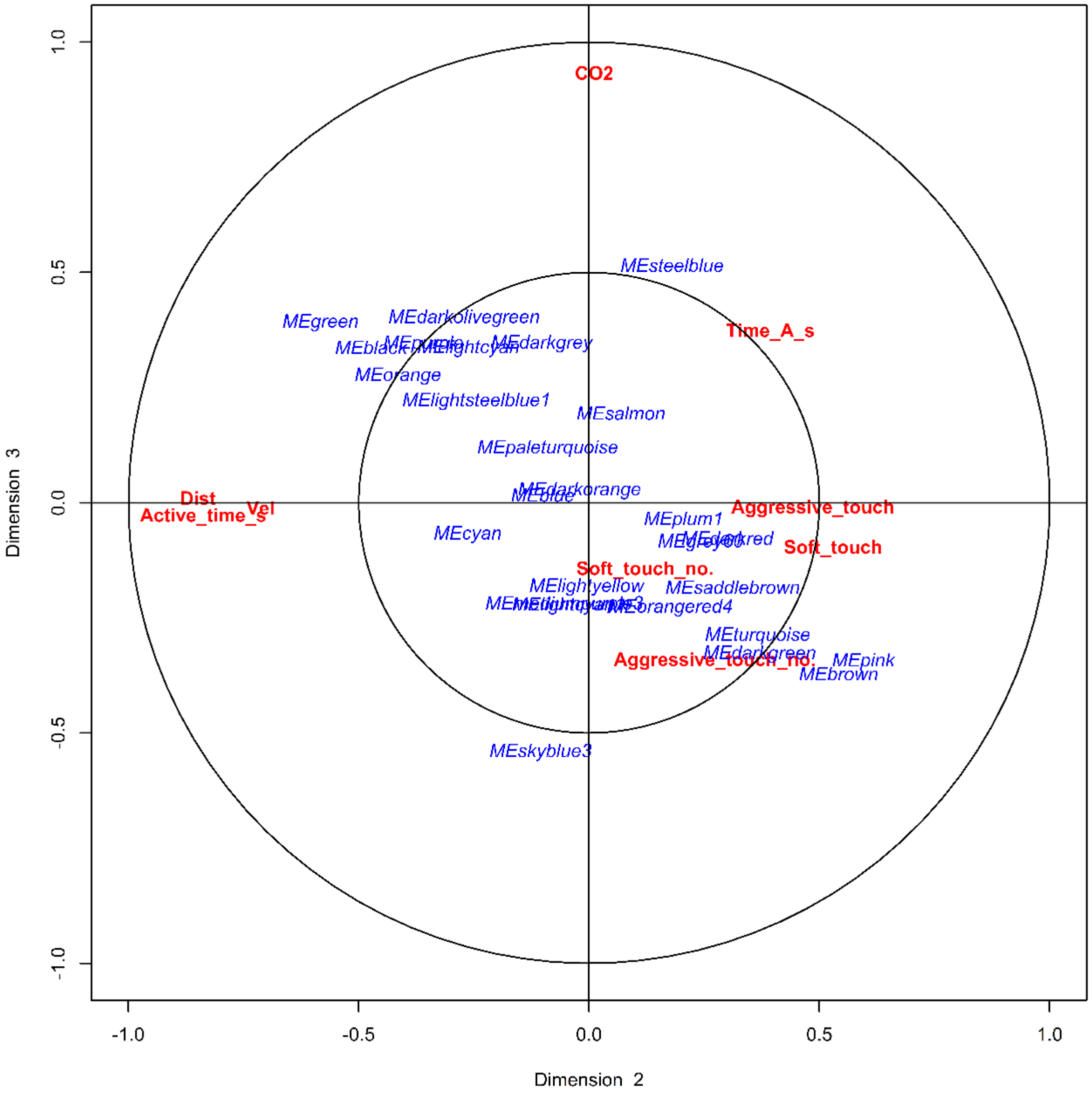

Figure S11. Canonical correlation analysis biplot for the CNS canonical functions 2 and 3. Depicting where the module eigengenes (MEs) lie in space in relation to the traits for the CNS, for canonical functions (dimensions) 2 and 3. Variables from the MEs set and the traits set are in blue and red, respectively. The inner ring is set at a radius of 0.5.

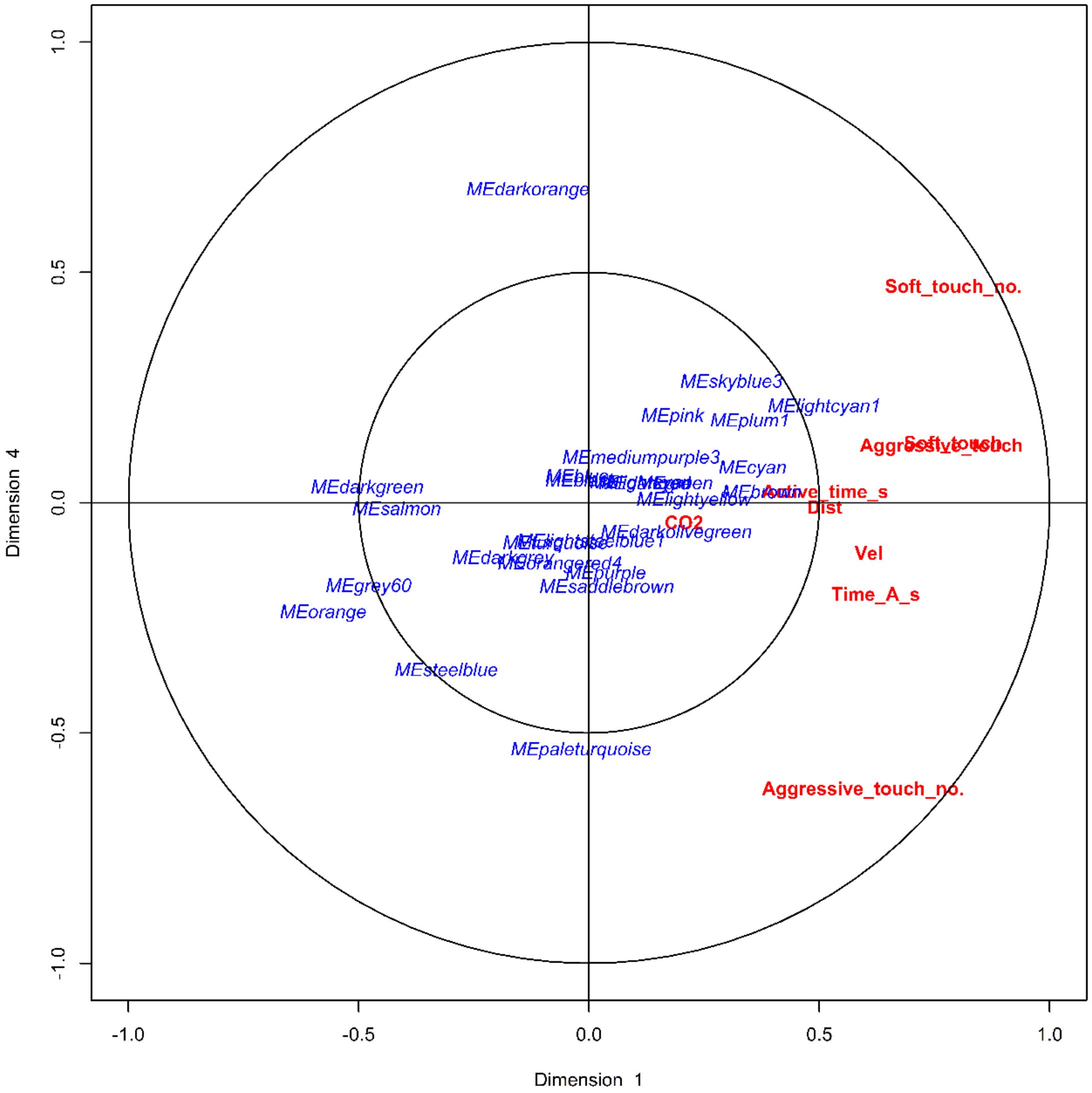

Figure S12. Canonical correlation analysis biplot for the CNS canonical functions 1 and 4. Depicting where the module eigengenes (MEs) lie in space in relation to the traits for the CNS, for canonical functions (dimensions) 1 and 4. Variables from the MEs set and the traits set are in blue and red, respectively. The inner ring is set at a radius of 0.5.

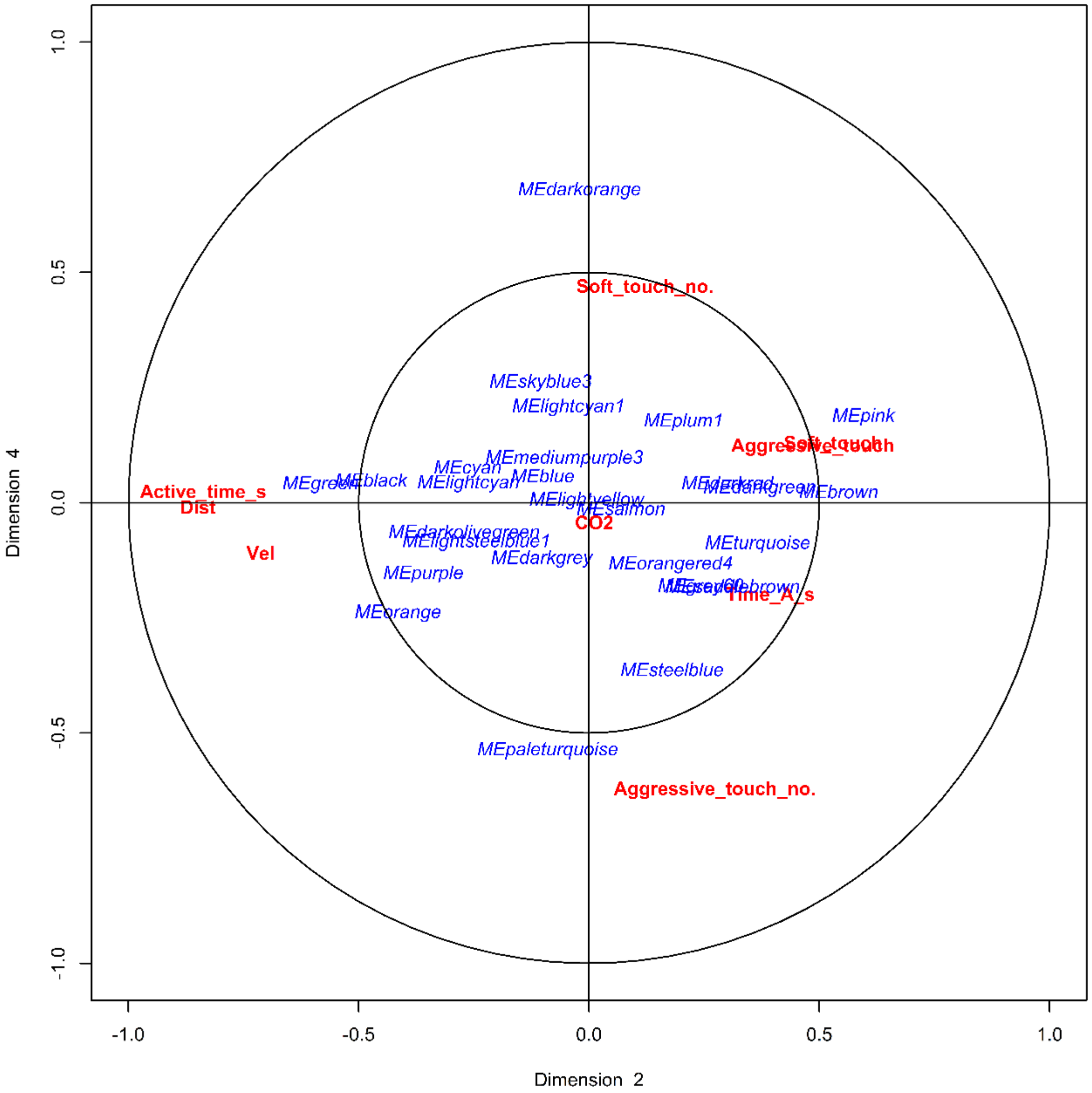

Figure S13. Canonical correlation analysis biplot for the CNS canonical functions 2 and 4. Depicting where the module eigengenes (MEs) lie in space in relation to the traits for the CNS, for canonical functions (dimensions) 2 and 4. Variables from the MEs set and the traits set are in blue and red, respectively. The inner ring is set at a radius of 0.5.

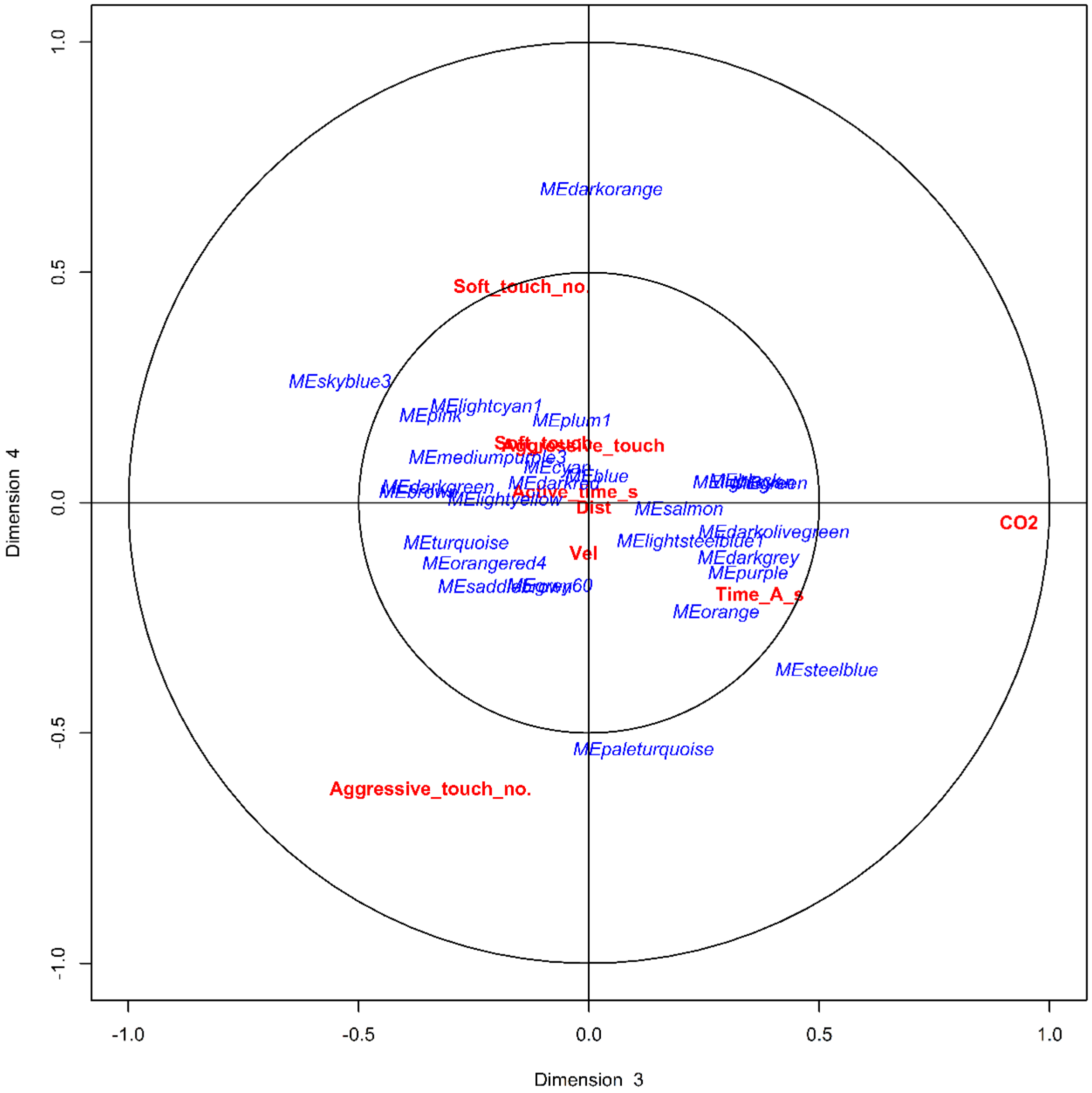

Figure S14. Canonical correlation analysis biplot for the CNS canonical functions 3 and 4. Depicting where the module eigengenes (MEs) lie in space in relation to the traits for the CNS, for canonical functions (dimensions) 3 and 4. Variables from the MEs set and the traits set are in blue and red, respectively. The inner ring is set at a radius of 0.5.

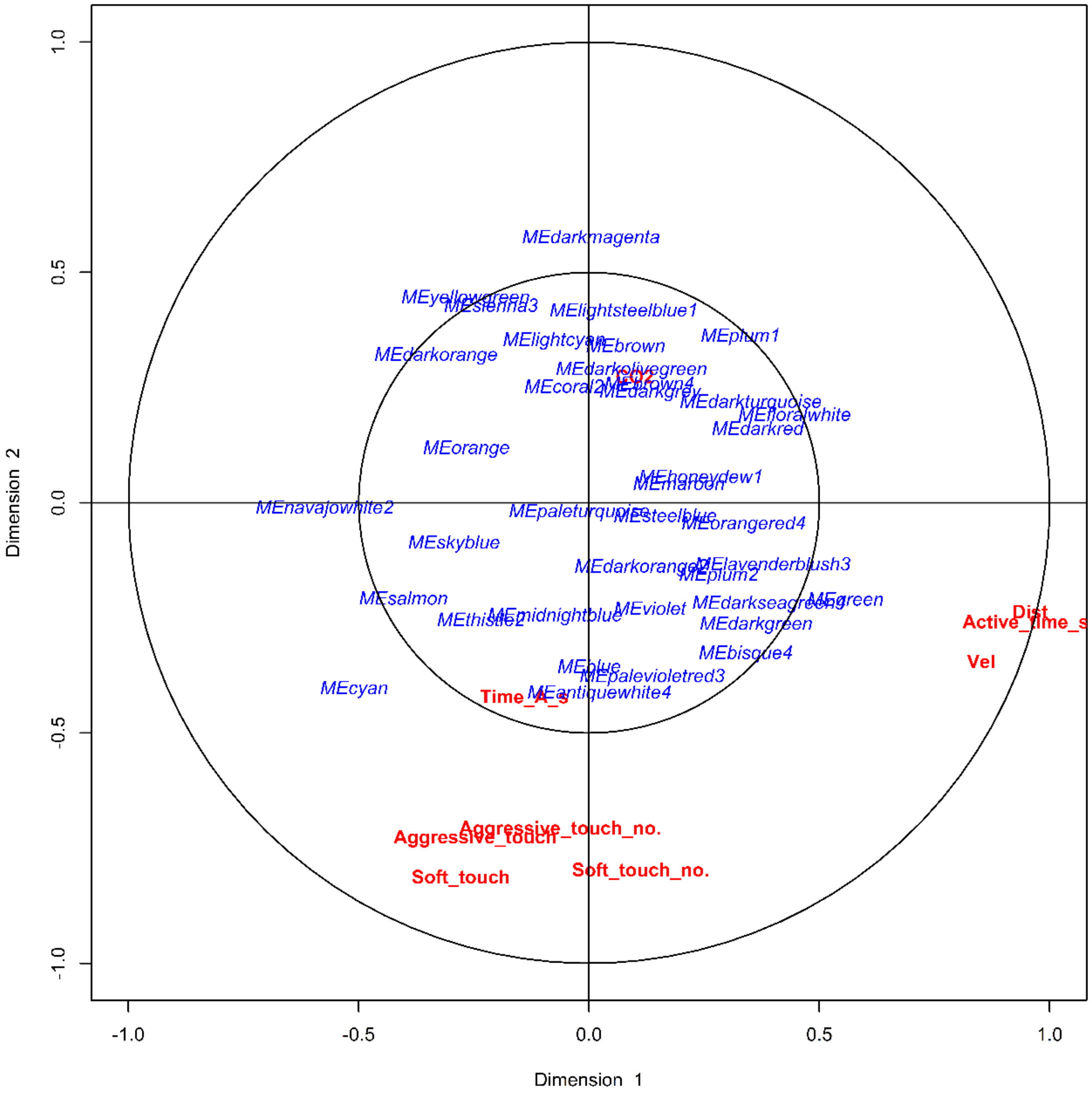

Figure S15. Canonical correlation analysis biplot for the eyes canonical functions 1 and 2. Depicting where the module eigengenes (MEs) lie in space in relation to the traits for the eyes, for canonical functions (dimensions) 1 and 2. Variables from the MEs set and the traits set are in blue and red, respectively. The inner ring is set at a radius of 0.5.

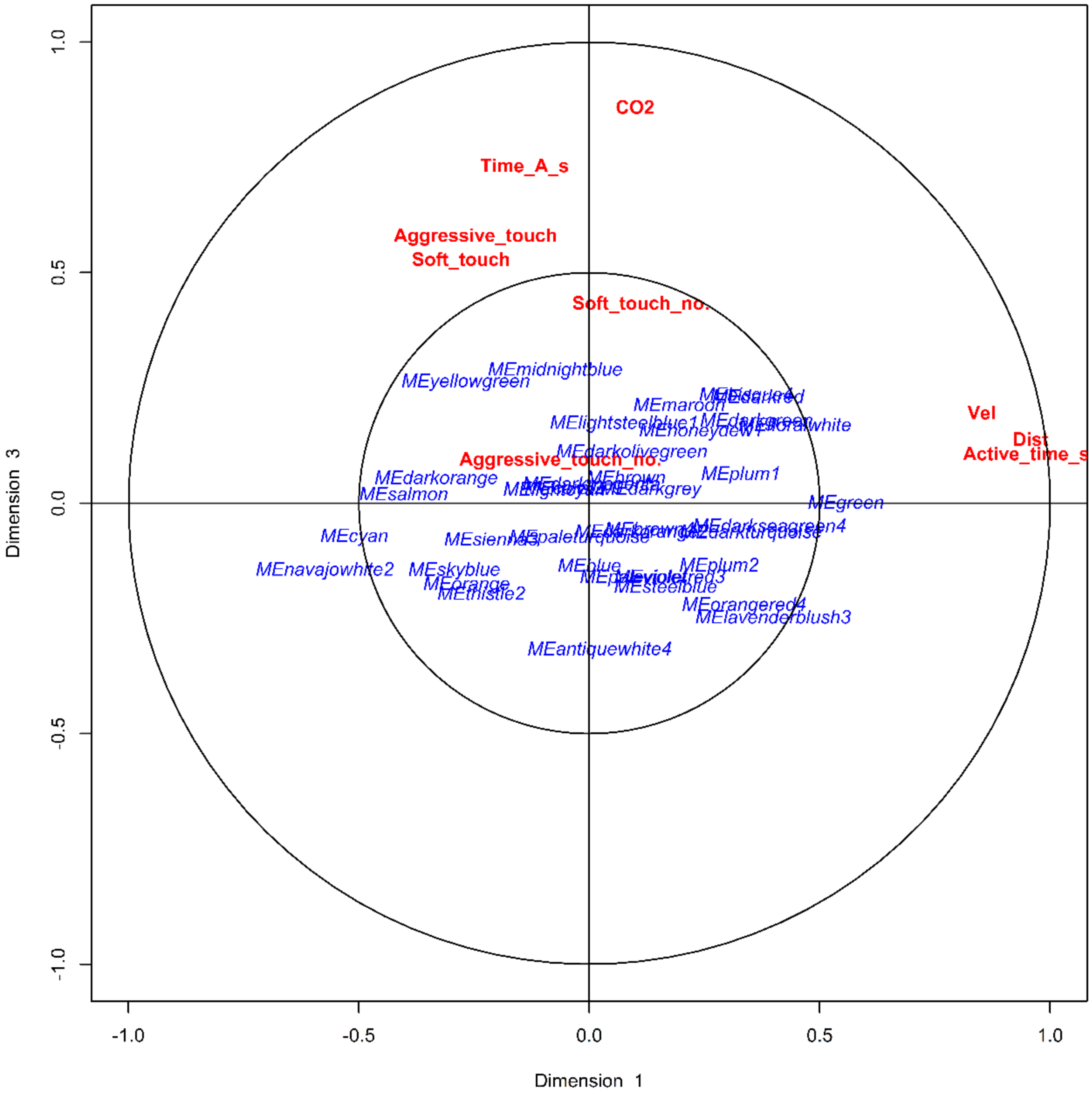

Figure S16. Canonical correlation analysis biplot for the eyes canonical functions 1 and 3. Depicting where the module eigengenes (MEs) lie in space in relation to the traits for the eyes, for canonical functions (dimensions) 1 and 3. Variables from the MEs set and the traits set are in blue and red, respectively. The inner ring is set at a radius of 0.5.

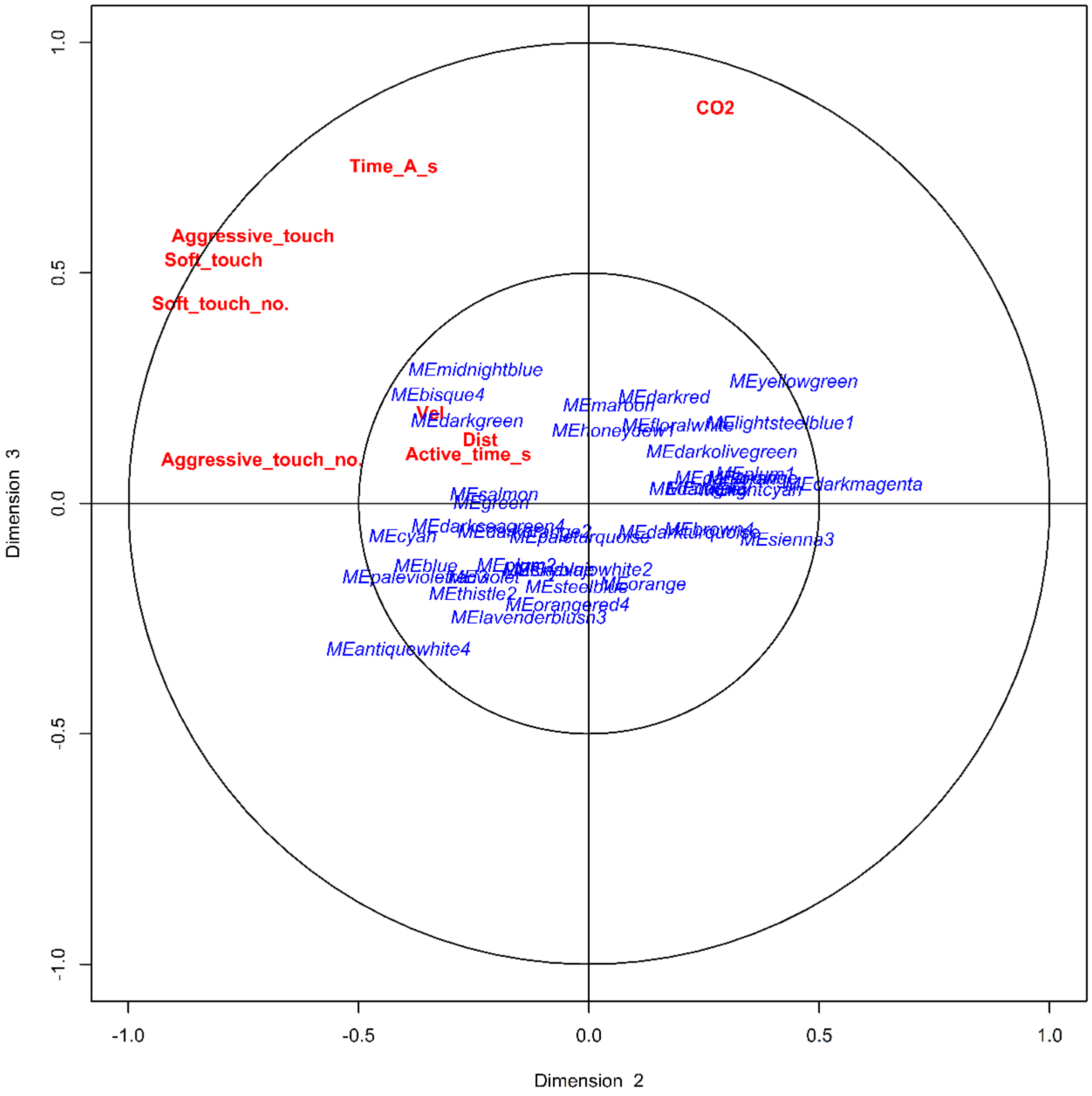

Figure S17. Canonical correlation analysis biplot for the eyes canonical functions 2 and 3. Depicting where the module eigengenes (MEs) lie in space in relation to the traits for the eyes, for canonical functions (dimensions) 2 and 3. Variables from the MEs set and the traits set are in blue and red, respectively. The inner ring is set at a radius of 0.5.

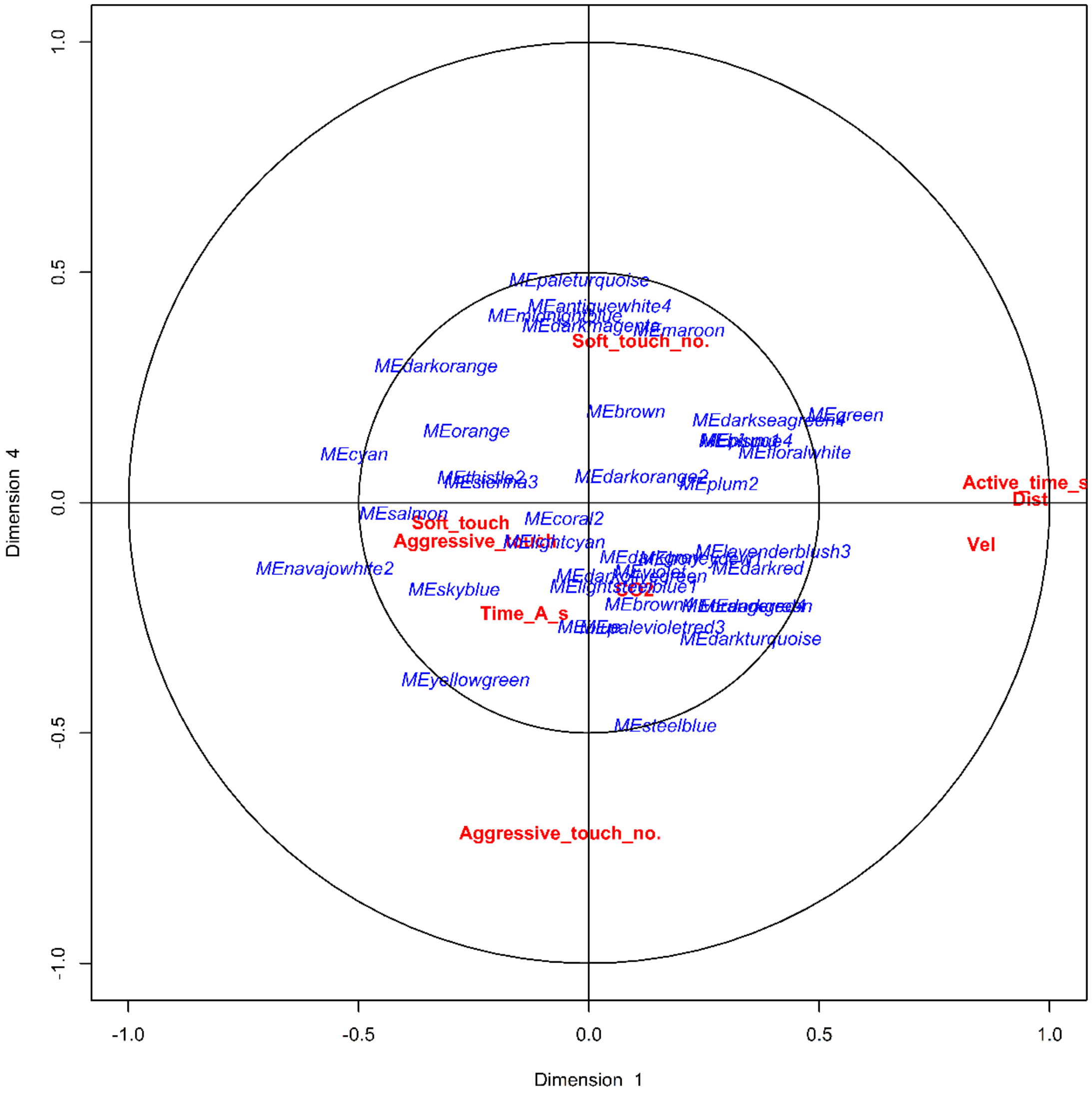

Figure S18. Canonical correlation analysis biplot for the eyes canonical functions 1 and 4. Depicting where the module eigengenes (MEs) lie in space in relation to the traits for the eyes, for canonical functions (dimensions) 1 and 4. Variables from the MEs set and the traits set are in blue and red, respectively. The inner ring is set at a radius of 0.5.

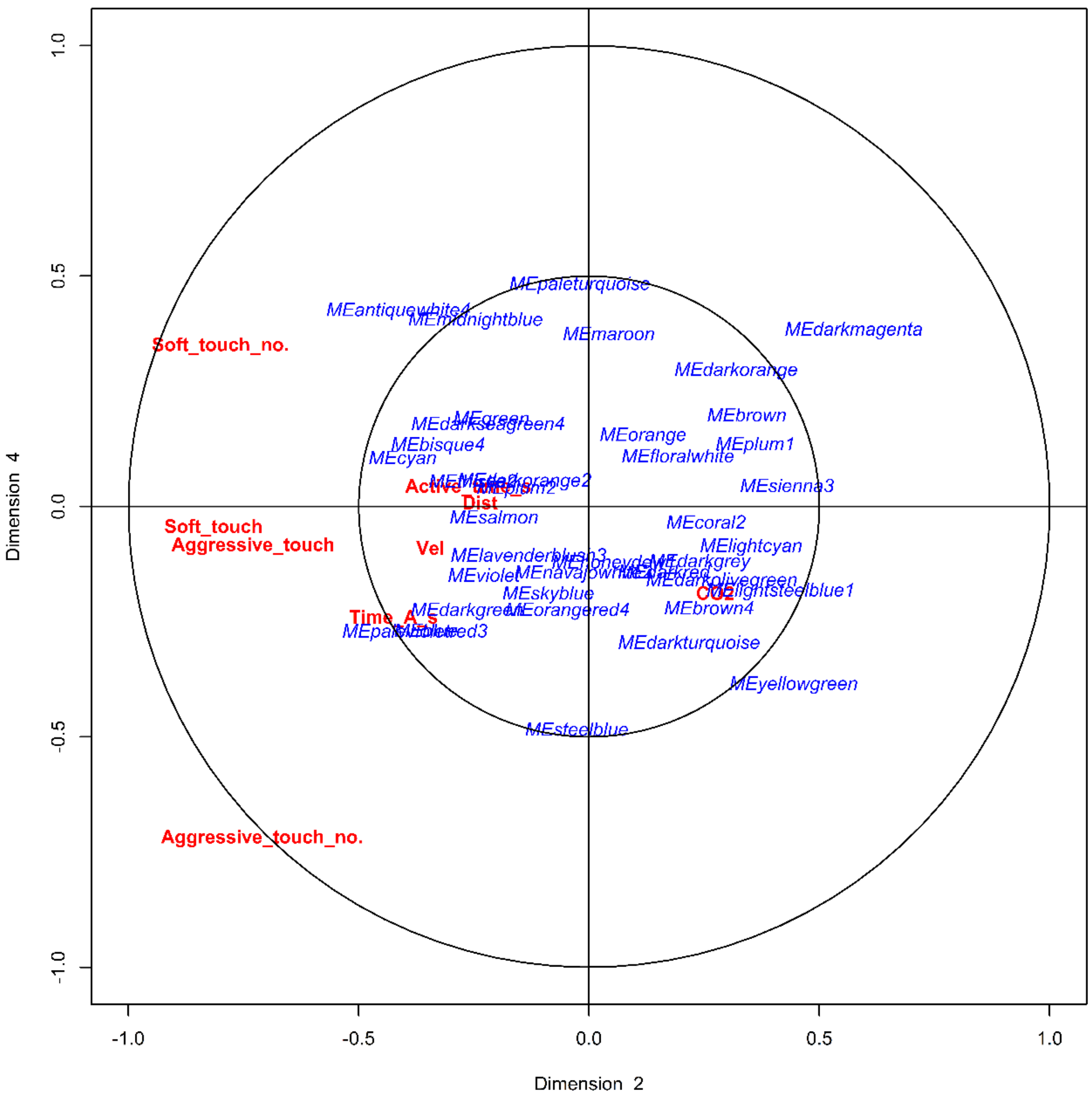

Figure S19. Canonical correlation analysis biplot for the eyes canonical functions 2 and 4. Depicting where the module eigengenes (MEs) lie in space in relation to the traits for the eyes, for canonical functions (dimensions) 2 and 4. Variables from the MEs set and the traits set are in blue and red, respectively. The inner ring is set at a radius of 0.5.

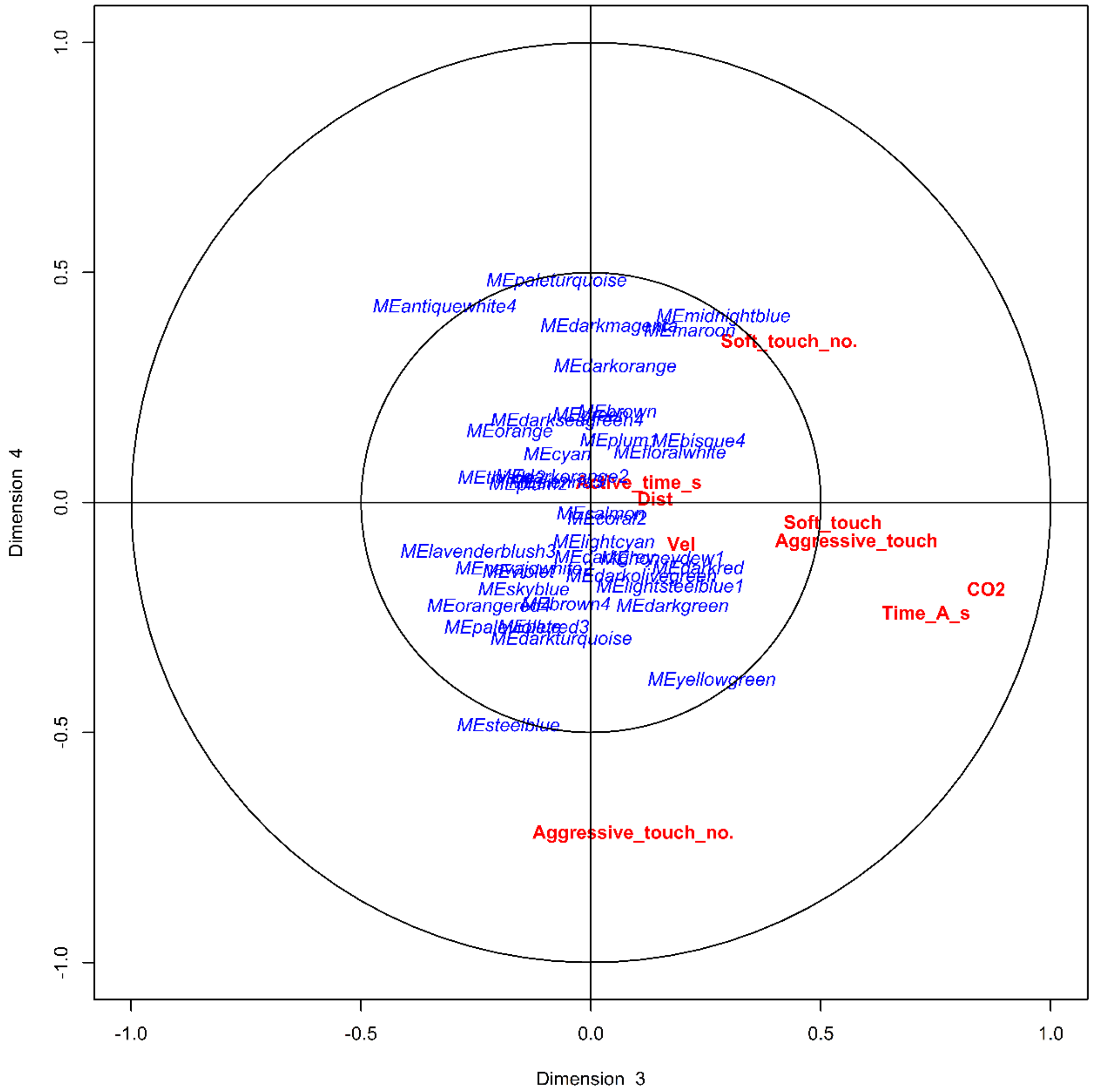

Figure S20. Canonical correlation analysis biplot for the eyes canonical functions 3 and 4. Depicting where the module eigengenes (MEs) lie in space in relation to the traits for the eyes, for canonical functions (dimensions) 3 and 4. Variables from the MEs set and the traits set are in blue and red, respectively. The inner ring is set at a radius of 0.5.

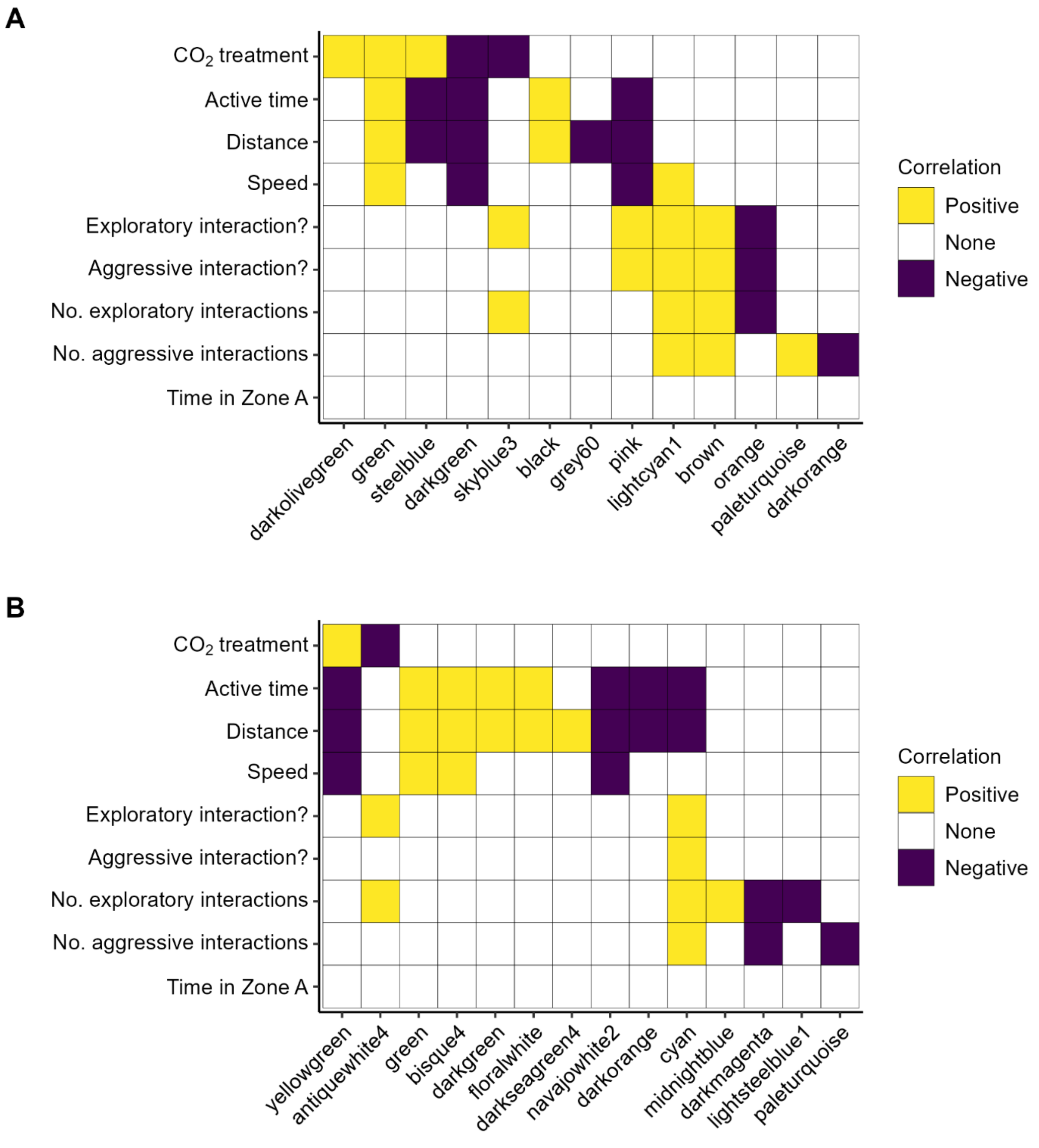

Figure S21. Final modules of interest in the A) CNS and B) eyes. Red = positive correlation between the trait and module eigengene, blue = negative correlation between the trait and module eigengene.

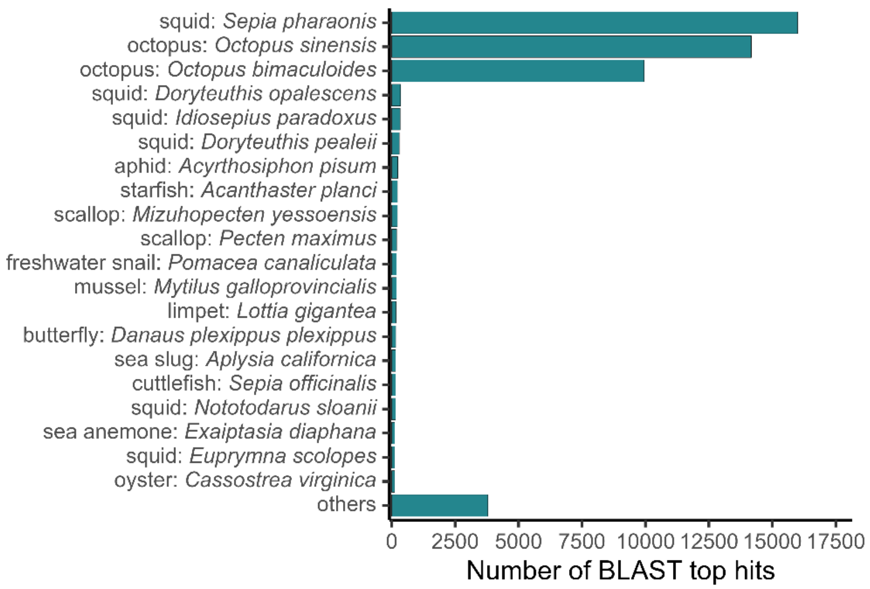

Figure S22. Species distribution for the top blast hits of the annotated transcriptome assembly. The top 29 species are shown. Others = the remaining 936 species grouped together.

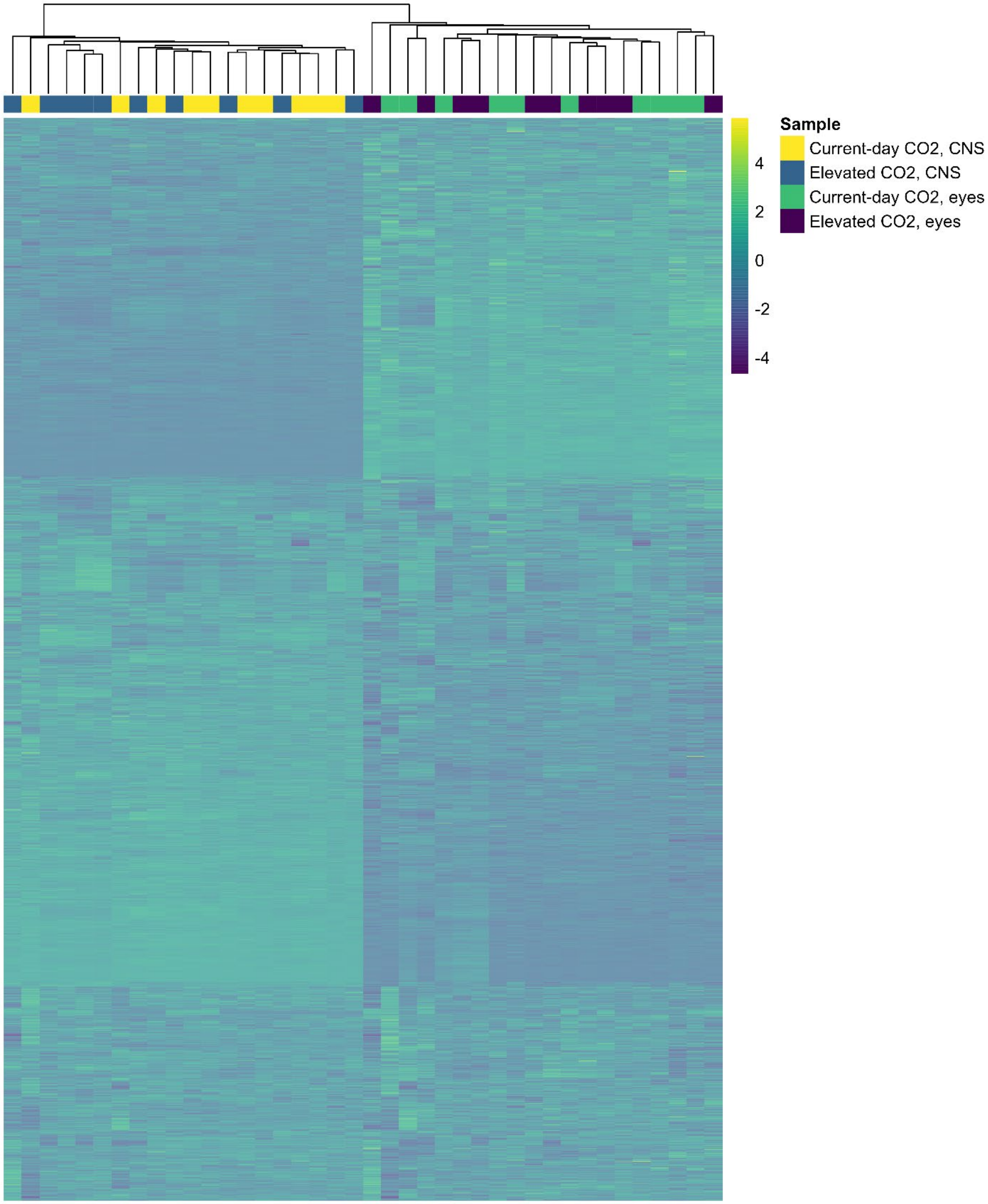

Figure S23. Heatmap of all samples showing the expression pattern of all genes in the CNS and eyes. Distance was calculated using the 'manhattan' method followed by hierarchical clustering using the 'average' method. Expression level is represented by Z-scores.

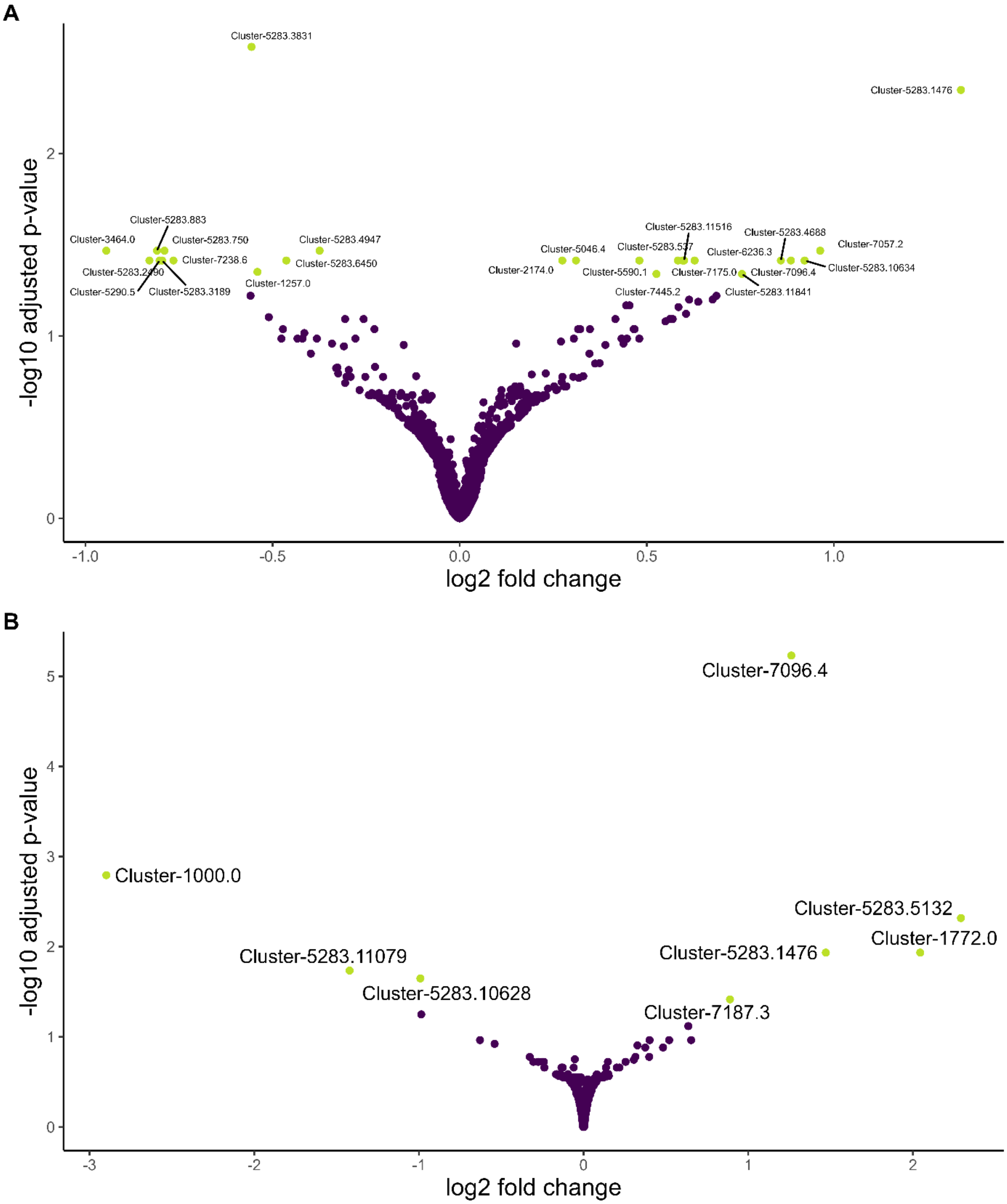

Figure S24. Volcano plots showing all genes in the A) CNS and B) eyes. Green = significantly differentially expressed genes (adjusted p-value (Benjamini-Hochberg method) < 0.05). Purple = all other genes.

See separate file for figure.

Figure S25. Dotplot showing the results from gene set enrichment analysis (GSEA) using GO terms/functional categories in the CNS and eyes. CNS = central nervous system, padj = adjusted p-value, count = number of core enrichment genes in the GO term/functional category.

Supplementary Tables

See separate excel file for tables

Table S1. Table of 27 modules detected in the CNS, with the number of genes within each module.

Table S2. Table of 38 modules detected in the eyes, with the number of genes within each module.

Table S3. Quality and completeness measures of the transcriptome assembly. The PacBio Sequel II produced a total of 138.6 million subreads which were used to assemble the de novo transcriptome. Before redundancy removal = ISO-seq data processed with the isoseq3 pipeline. After redundancy removal = ISO-seq data processed with the isoseq3 pipeline followed by redundancy removal with CD-HIT-EST. Final transcriptome assembly = ISO-seq data processed with the isoseq3 pipeline, followed by redundancy removal with CD-HIT-EST and only the transcripts containing an ORF, as identified by TransDecoder, were retained. bp = base pairs.

Table S4. Annotation measures of the transcriptome assembly.

Table S5. RNA-sequencing information for each sample. CNS = central nervous system, eyes = both eyes combined from the same individual, % mapping rate is mapping of the trimmed and decontaminated RNA-seq reads against the final transcriptome assembly.

Table S6. Table of DEGs and their putative function in the CNS and eyes, ordered by LFC. Genes in yellow and purple are upregulated and downregulated at elevated CO2, respectively. DEGs in both tissues are in bold. padj = adjusted p-value, LFC = log2 fold change.

Table S7. Eyes-specific CO_2 treatment hub genes. For each of these CO_2 treatment hub genes, those genes also identified as a hub gene for one or more behavioural traits in the eyes are indicated in the columns on the right (active time, distance, speed, no. exploratory interactions). The numbers in each column are the gene significance (GS) for the corresponding trait (-1 to 1). Positive GS = positive correlation between gene expression and trait (in yellow), negative GS = negative correlation between gene expression and trait (in purple). The larger the GS absolute value the more biologically relevant the gene is. Dist. = distance, No. EI = number of exploratory interactions.

Table S8. Hub genes identified for CO_2 treatment in both the CNS and eyes. For each of these CO_2 treatment hub genes, those genes also identified as a hub gene for a behavioural trait in the CNS or eyes are indicated in the columns on the right (active time, distance, speed, no. exploratory interactions). The numbers in each column are the gene significance (GS) for the corresponding trait (-1 to 1). Positive GS = positive correlation between gene expression and trait (in yellow), negative GS = negative correlation between gene expression and trait (in purple). The

larger the GS absolute value the more biologically relevant the gene is. Dist. = distance, No. EI = number of exploratory interactions.

Table S9. CNS-specific CO_2 treatment hub genes. For each of these CO_2 treatment hub genes, those genes also identified as a hub gene for one or more behavioural traits in the CNS are indicated in the columns on the right (active time, distance, speed, no. exploratory interactions, exploratory interaction?). The numbers in each column are the gene significance (GS) for the corresponding trait (-1 to 1). Positive GS = positive correlation between gene expression and trait (in yellow), negative GS = negative correlation between gene expression and trait (in purple). The larger the GS absolute value the more biologically relevant the gene is. Dist. = distance, No. EI = number of exploratory interactions, Exploratory interaction? = whether any exploratory interactions occurred (yes/no).

Table S10. List of function groups and related CNS-specific hub genes shared by CO_2 treatment and one or more activity traits.

Table S11. All core enrichment genes from the cluster of GO terms/functional categories related to ligand-gated ion channels that were found significant with gene set enrichment analysis in both the CNS and eyes. The ion channel cluster includes the GO terms 'ionotropic glutamate receptor signaling pathway', 'ionotropic glutamate receptor activity', 'postsynaptic membrane', 'excitatory postsynaptic potential', 'ion transmembrane transport', 'acetylcholine-gated cation-selective channel activity', 'transmembrane signaling receptor activity', 'extracellular ligand-gated ion channel activity', 'synapse', and 'ion channel activity'. Gene = gene ID in the transcriptome, annotation = annotation of the corresponding gene, ordered alphabetically.

Table S12. All core enrichment genes from the cluster of GO terms/functional categories related to GPCR (G-protein coupled receptors) that were found significant with gene set enrichment analysis in the CNS (no GPCR GO terms found significant in the eyes). The GPCR cluster includes the GO terms 'G protein-coupled receptor activity' and 'G protein-coupled receptor signaling pathway'. Gene = gene ID in the transcriptome, annotation = annotation of the corresponding gene, ordered alphabetically.

Table S13. All core enrichment genes from the cluster of GO terms/functional categories related to ion transport that were found significant with gene set enrichment analysis in the CNS (no ion transport GO terms found significant in the eyes). The ion transport cluster includes the GO terms 'potassium channel activity', 'potassium ion transmembrane transport', 'voltage-gated potassium channel activity', 'regulation of ion transmembrane transport', 'voltage-gated calcium channel complex', and 'calcium ion transmembrane transport'. Gene = gene ID in the transcriptome, annotation = annotation of the corresponding gene, ordered alphabetically.

File S1. TapeStation electropherograms for each of the 40 RNA samples used for RNA-sequencing. Each sample is labelled by its individual ID and the tissue type. RNA integrity of all 40 samples was measured on an Agilent 2200 TapeStation (High Sensitivity RNA ScreenTape, Agilent), without the sample denaturation step due to denaturation removing the 28S peak, likely due to a 'hidden break' as reported in some other animals (Winnebeck *et al.*, 2010). All central nervous system (CNS) RNA had an equivalent RNA integrity (RINe) ≥ 8.5 (mean 9.3, SD 0.3). For the eye samples, a RINe value could not be obtained due to the TapeStation being unable to detect the lower marker, likely due to carry over of pigment into the eye RNA samples. A Femto Pulse system (Ultra Sensitivity RNA Kit, Agilent) did obtain RNA Quality Scores (RQN): eye RQN ≥ 4.8 (mean 6.5, SD 1.3). The four samples also used for ISO-sequencing are outlined in thick black. CNS samples used for ISO-seq had RINe values of 9.4 and 9.5. Before ISO-seq, RNA from the eyes was purified with oligo d(T) beads due to carry over of pigmentation (NEBNext® Poly(A) mRNA Magnetic Isolation Module, New England BioLabs Inc.), followed by integrity assessment on a Femto Pulse system (Ultra Sensitivity RNA Kit, Agilent). After purification, eyes samples had an RQN of 6.1 and 8.6.

See separate file.

References

BioBam Bioinformatics. (2019). *OmicsBox - Bioinformatics made easy (Version 1.4.12).* https://www.biobam.com/omicsbox.

Brian, H. and Papanicolaou, A. (n.d.). *Transdecoder (Find Coding Regions Within Transcripts).* http://transdecoder.github.io.

Camacho, C., Coulouris, G., Avagyan, V., Ma, N., Papadopoulos, J., Bealer, K. and Madden, T. L. (2009). BLAST+: Architecture and applications. *BMC Bioinformatics* **10**, 1-9.

Dickson, A. and Millero, F. J. (1987). A comparison of the equilibrium constants for the dissociation of carbonic acid in seawater media. *Deep Sea Research Part A. Oceanographic Research Papers* **34**, 1733-1743.

Dickson, A. G., Sabine, C. L. and Christian, J. R. (2007). *Guide to Best Practices for Ocean CO_2 Measurements. PICES Special Publication 3.* Sidney, Canada: North Pacific Marine Science Organization.

Evans, J. D. (1996). *Straightforward Statistics for the Behavioral Sciences*: Thomson Brooks/Cole Publishing Co.

Fu, L., Niu, B., Zhu, Z., Wu, S. and Li, W. (2012). CD-HIT: Accelerated for clustering the next-generation sequencing data. *Bioinformatics* **28**, 3150-3152.

Fuller, T. F., Ghazalpour, A., Aten, J. E., Drake, T. A., Lusis, A. J. and Horvath, S. (2007). Weighted gene coexpression network analysis strategies applied to mouse weight. *Mammalian Genome* **18**, 463-472.

González, I., Déjean, S., Martin, P. and Baccini, A. (2008). CCA: An R package to extend canonical correlation analysis. *Journal of Statistical Software* **23**, 1-14.

Götz, S., García-Gómez, J. M., Terol, J., Williams, T. D., Nagaraj, S. H., Nueda, M. J., Robles, M., Talón, M., Dopazo, J. and Conesa, A. (2008). High-throughput functional annotation and data mining with the Blast2GO suite. *Nucleic Acids Research* **36**, 3420-3435.

Hannan, K. D., Miller, G. M., Watson, S.-A., Rummer, J. L., Fabricius, K. and Munday, P. L. (2020). Diel pCO_2 variation among coral reefs and microhabitats at Lizard Island, Great Barrier Reef. *Coral Reefs* **39**, 1391-1406.

Horvath, S. and Dong, J. (2008). Geometric interpretation of gene coexpression network analysis. *PLoS Computational Biology* **4**, e1000117-e1000117.

Jones, P., Binns, D., Chang, H.-Y., Fraser, M., Li, W., McAnulla, C., McWilliam, H., Maslen, J., Mitchell, A., Nuka, G. et al. (2014). InterProScan 5: Genome-scale protein function classification. *Bioinformatics* **30**, 1236-1240.

Kabir, A., Merrill, R. D., Shamim, A. A., Klemn, R. D., Labrique, A. B., Christian, P., West Jr, K. P. and Nasser, M. (2014). Canonical correlation analysis of infant's size at birth and maternal factors: a study in rural Northwest Bangladesh. *PloS One* **9**, e94243.

Lambert, Z. V. and Durand, R. M. (1975). Some precautions in using canonical analysis. *Journal of Marketing Research* **12**, 468-475.

Langfelder, P. and Horvath, S. (2008). WGCNA: An R package for weighted correlation network analysis. *BMC Bioinformatics* **9**, 1-13.

Langmead, B. and Salzberg, S. L. (2012). Fast gapped-read alignment with Bowtie 2. *Nature Methods* **9**, 357-359.

Li, W. and Godzik, A. (2006). Cd-hit: A fast program for clustering and comparing large sets of protein or nucleotide sequences. *Bioinformatics* **22**, 1658-1659.

Love, M. I., Huber, W. and Anders, S. (2014). Moderated estimation of fold change and dispersion for RNA-seq data with DESeq2. *Genome Biology* **15**, 550.

Manni, M., Berkeley, M. R., Seppey, M., Simão, F. A. and Zdobnov, E. M. (2021). BUSCO update: Novel and streamlined workflows along with broader and deeper phylogenetic coverage for scoring of eukaryotic, prokaryotic, and viral genomes. *Molecular Biology and Evolution* **38**, 4647-4654.

Mehrbach, C., Culberson, C., Hawley, J. and Pytkowicx, R. (1973). Measurement of the apparent dissociation constants of carbonic acid in seawater at atmospheric pressure 1. *Limnology and Oceanography* **18**, 897-907.

Sherry, A. and Henson, R. K. (2005). Conducting and interpreting canonical correlation analysis in personality research: A user-friendly primer. *Journal of personality assessment* **84**, 37-48.

Smith-Unna, R., Boursnell, C., Patro, R., Hibberd, J. M. and Kelly, S. (2016). TransRate: Reference-free quality assessment of *de novo* transcriptome assemblies. *Genome Research* **26**, 1134-1144.

Winnebeck, E. C., Millar, C. D. and Warman, G. R. (2010). Why does insect RNA look degraded? *Journal of Insect Science* **10**, 159.
