# Supplementary figures and images for: Transcriptomic responses in the nervous system and correlated behavioural changes of a cephalopod exposed to ocean acidification
Source: BMC Genomics. 2024 Jun 25;25:635. doi: 10.1186/s12864-024-10542-5 (PMC11202396; doi:10.1186/s12864-024-10542-5)

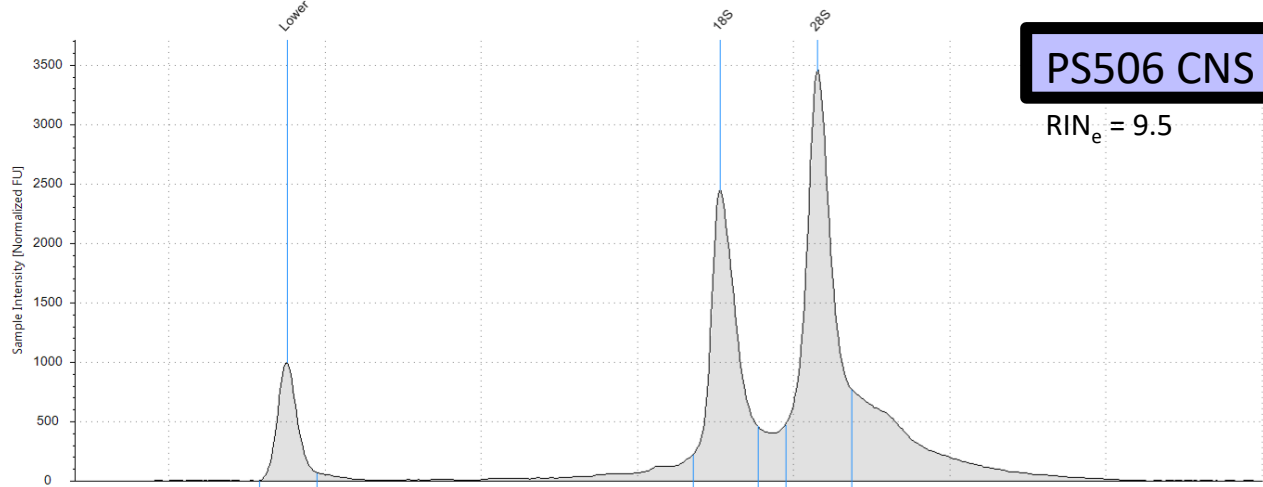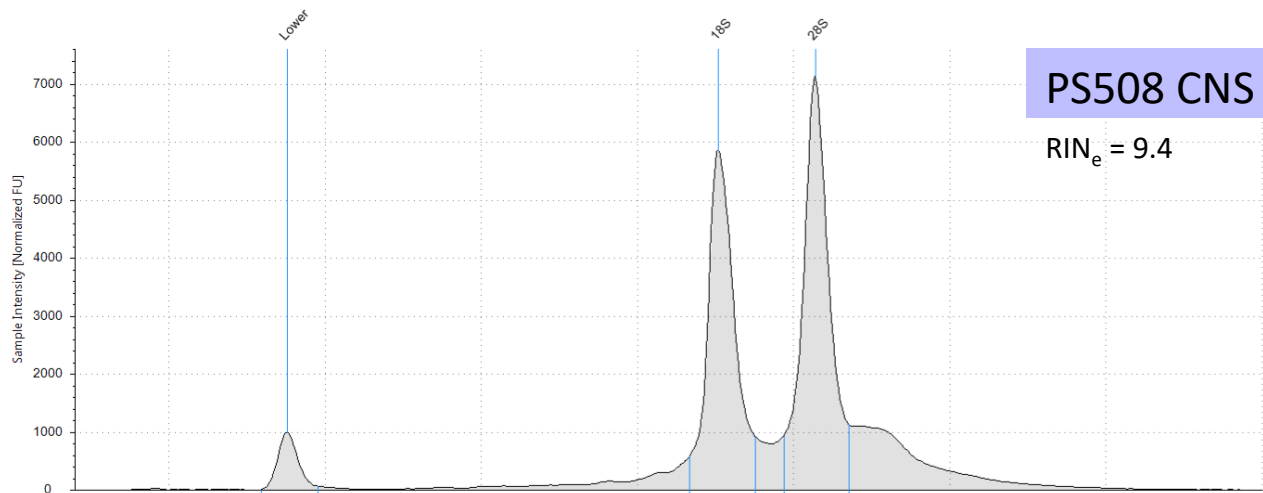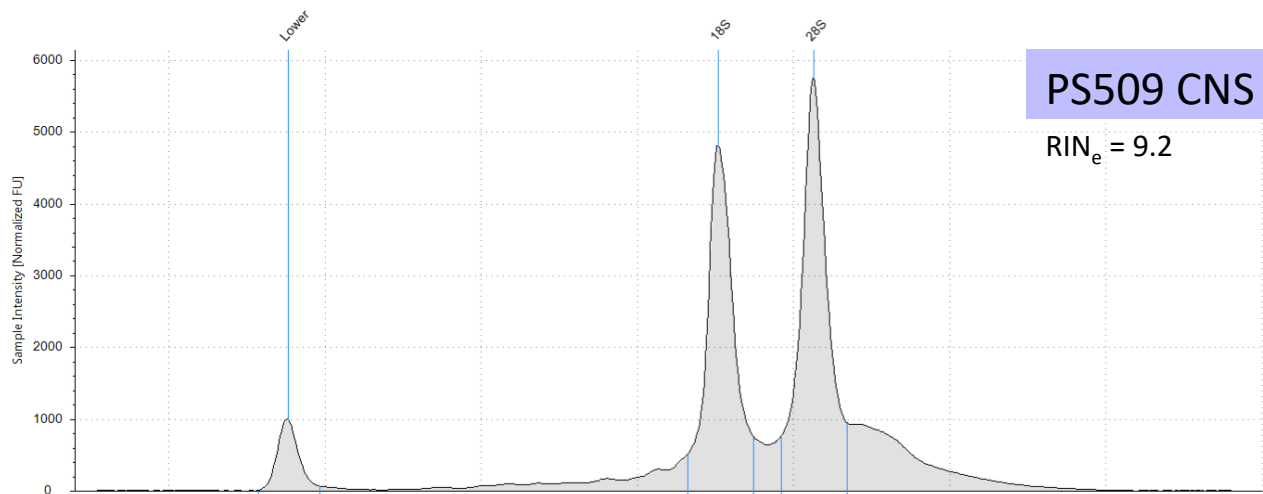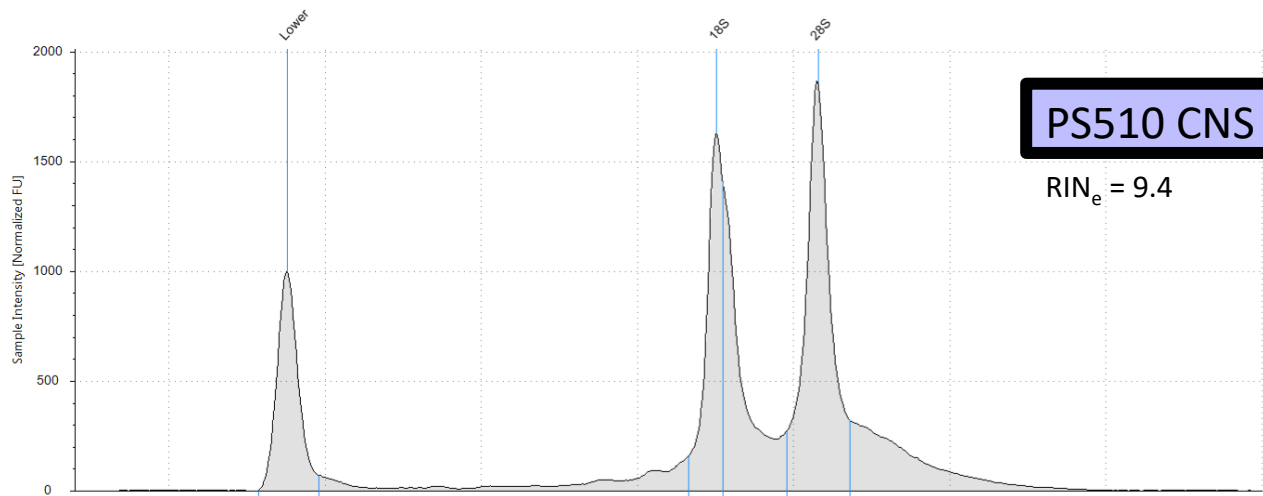

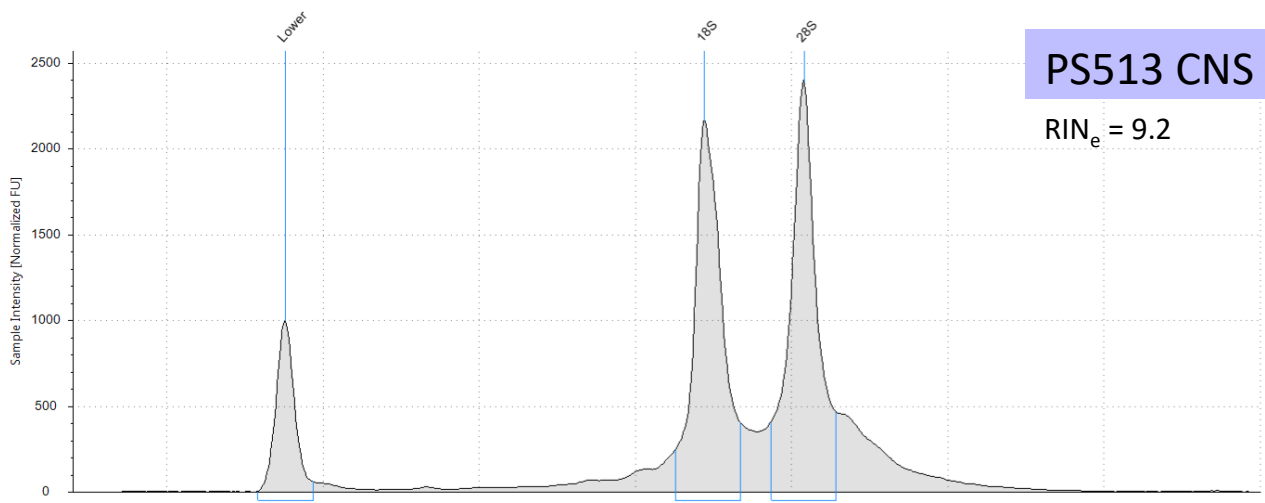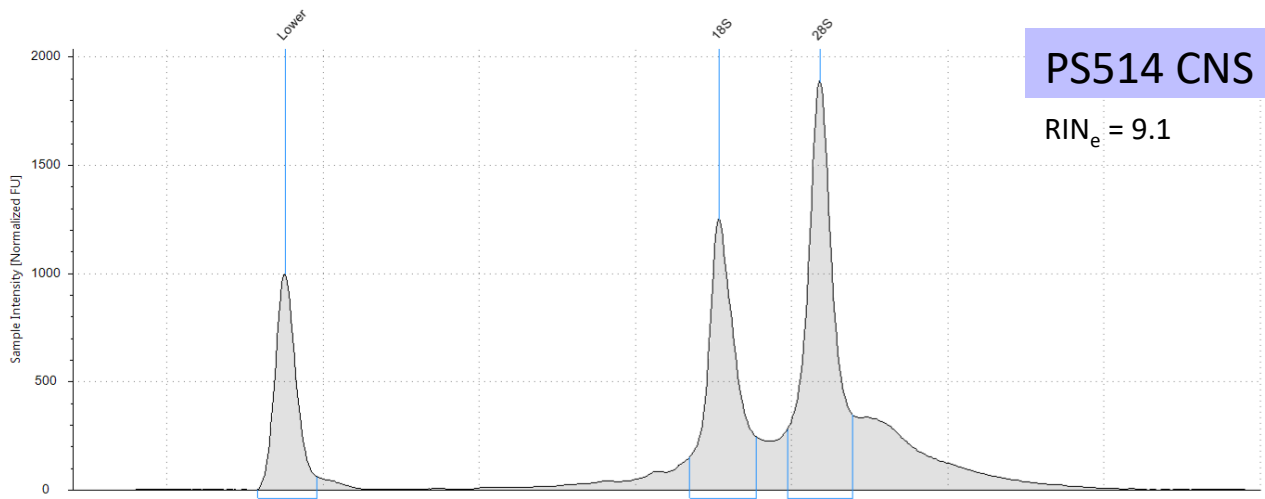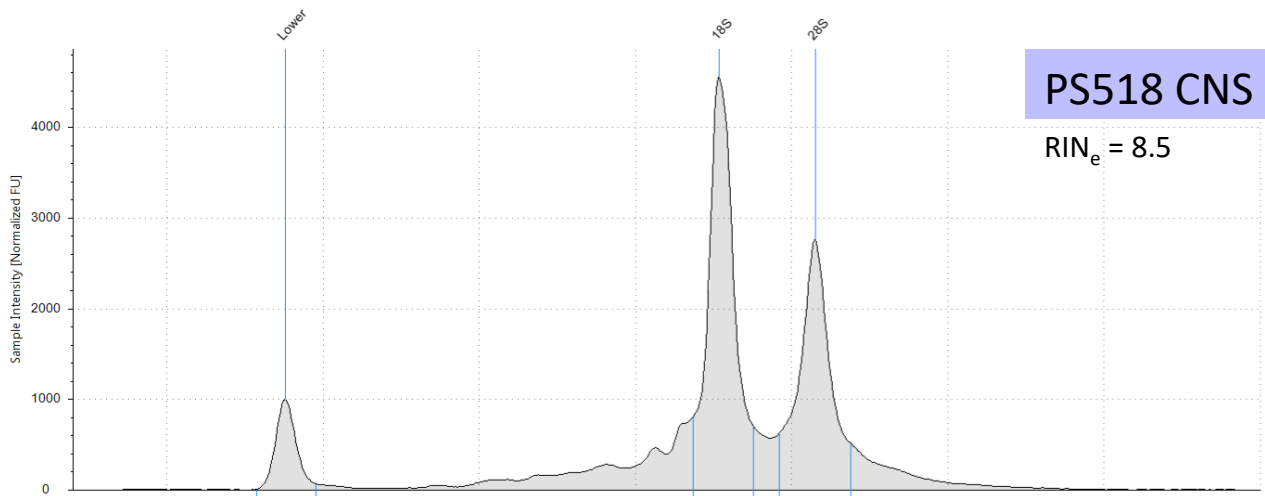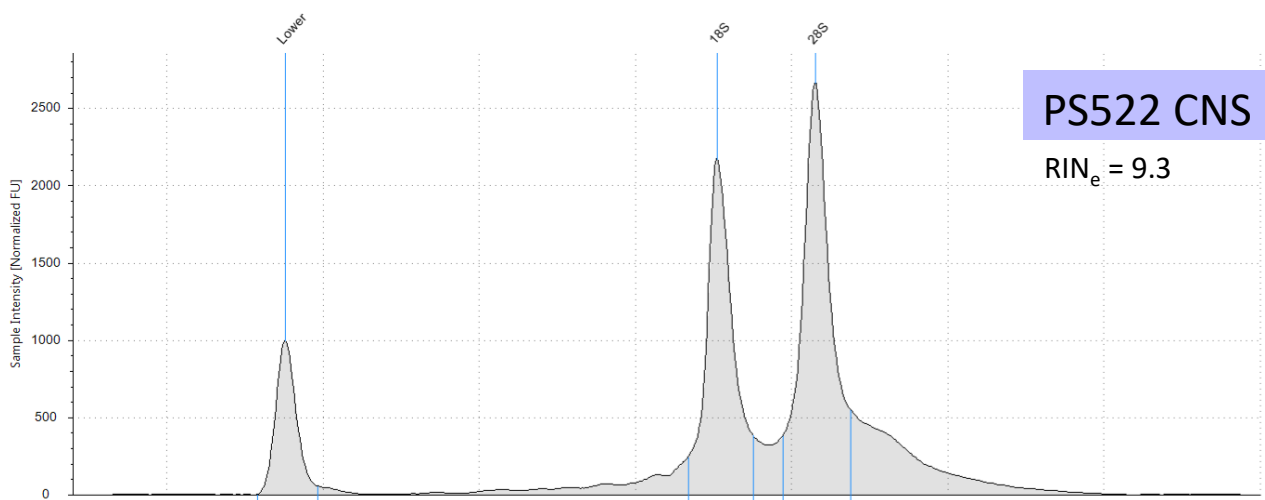

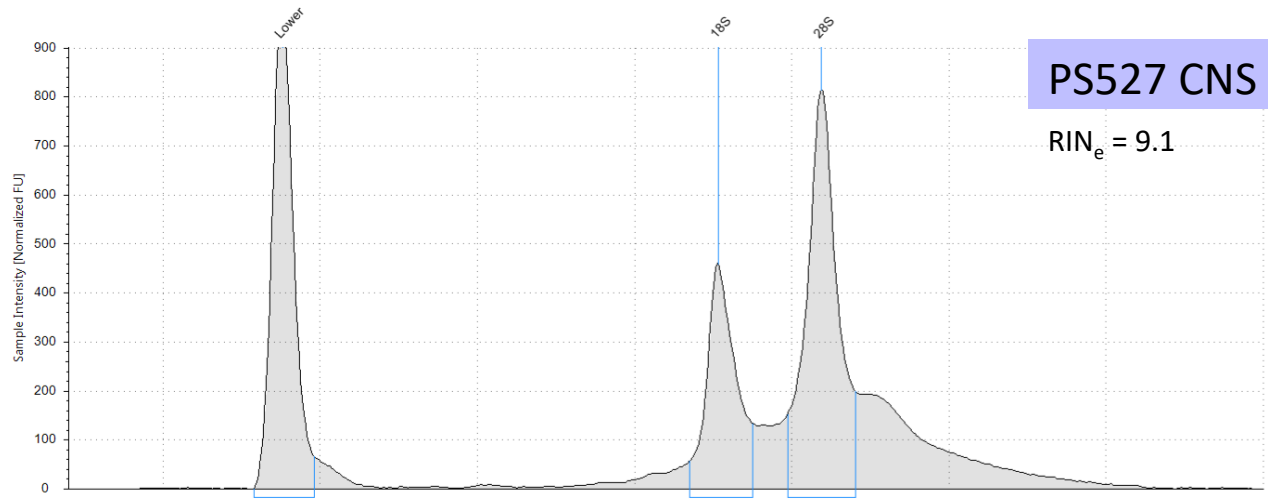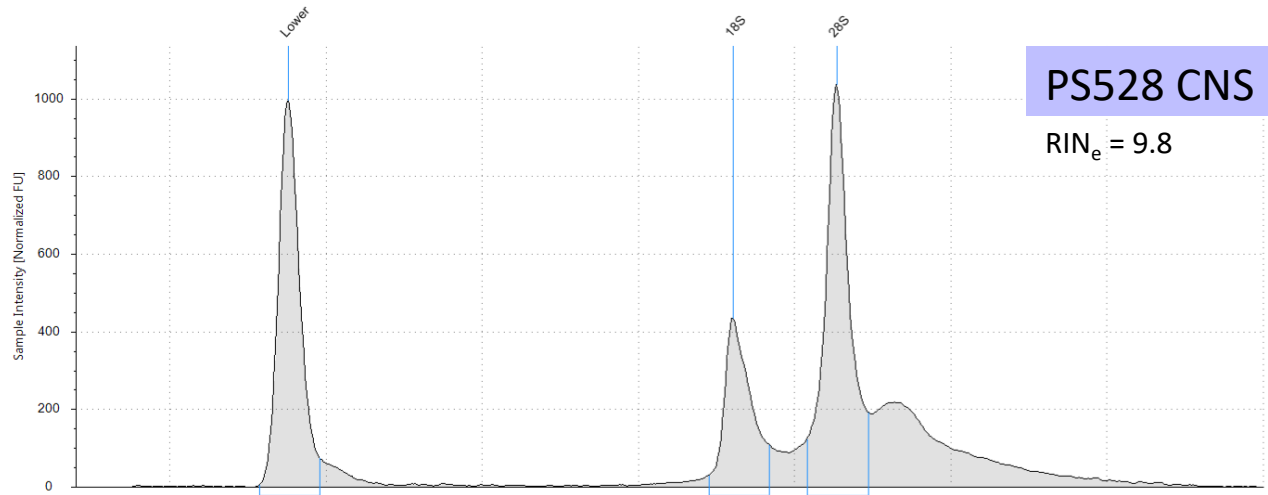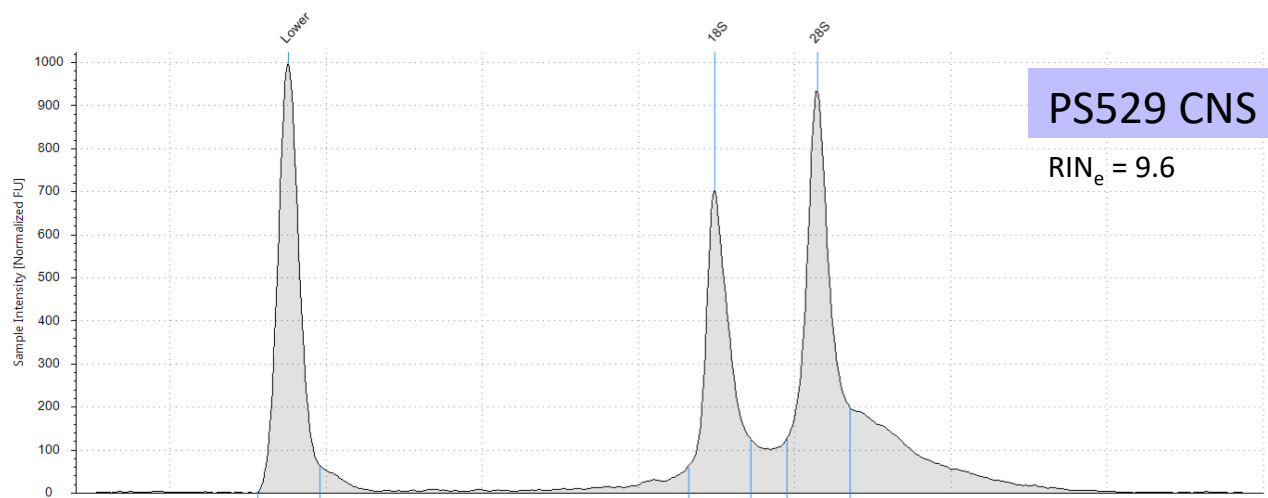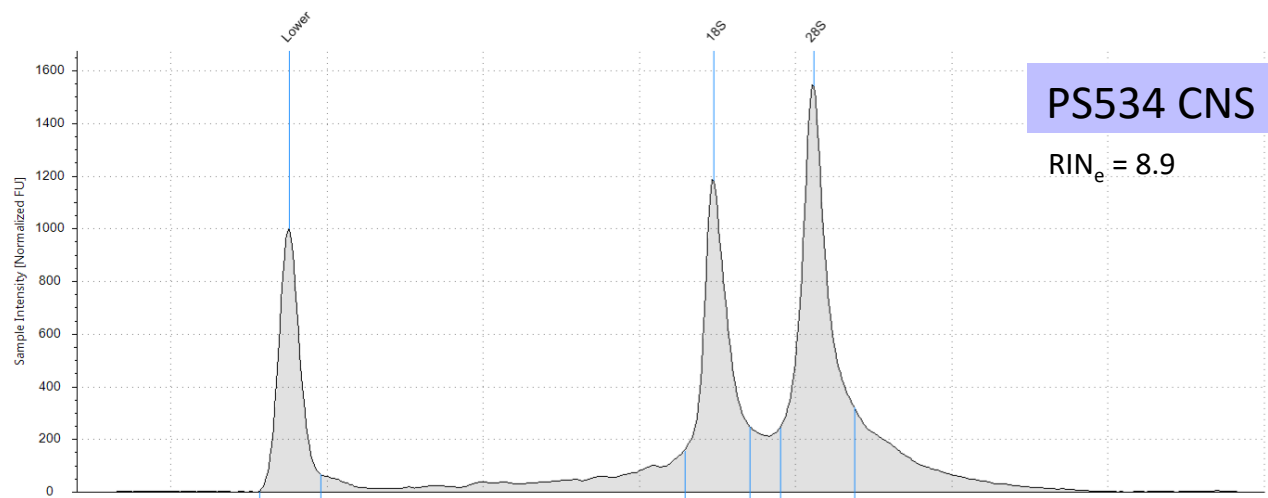

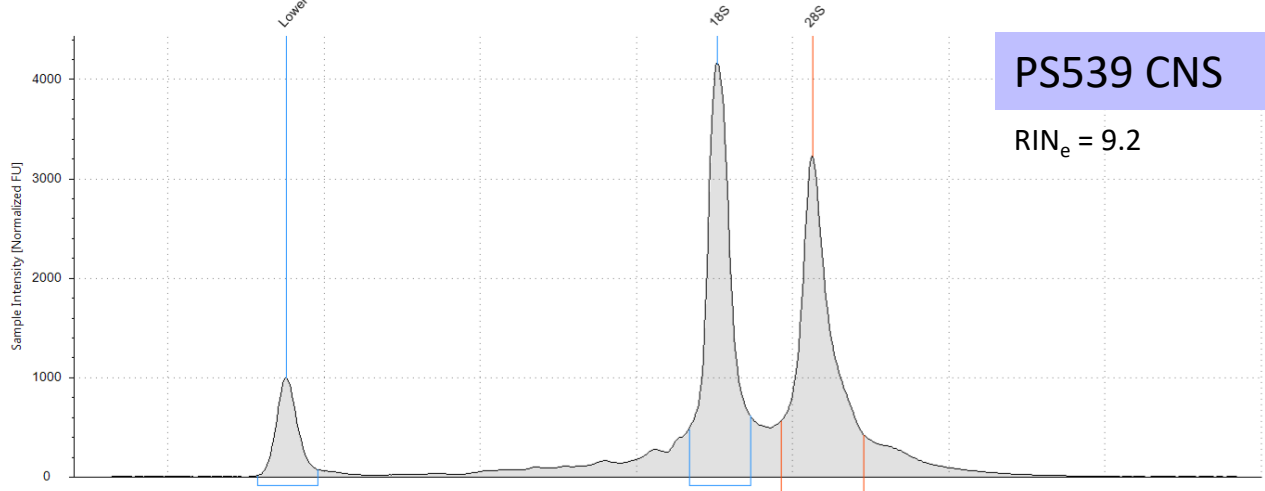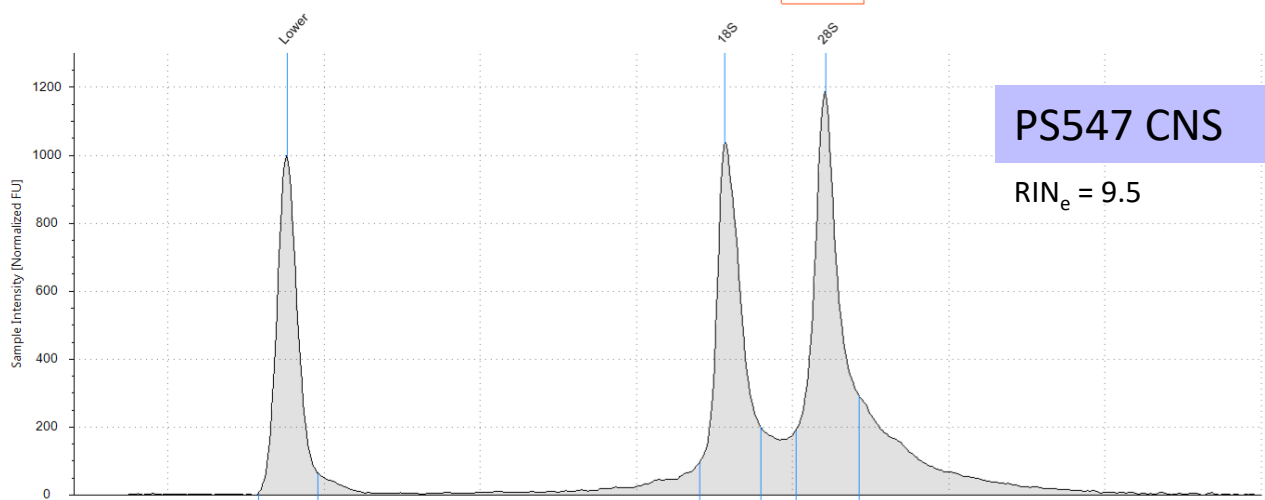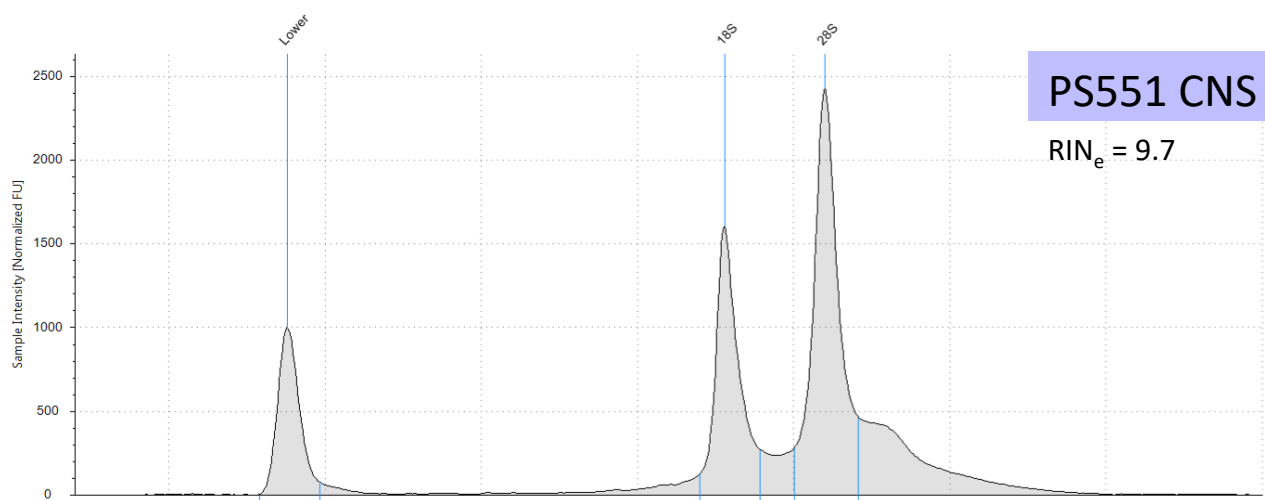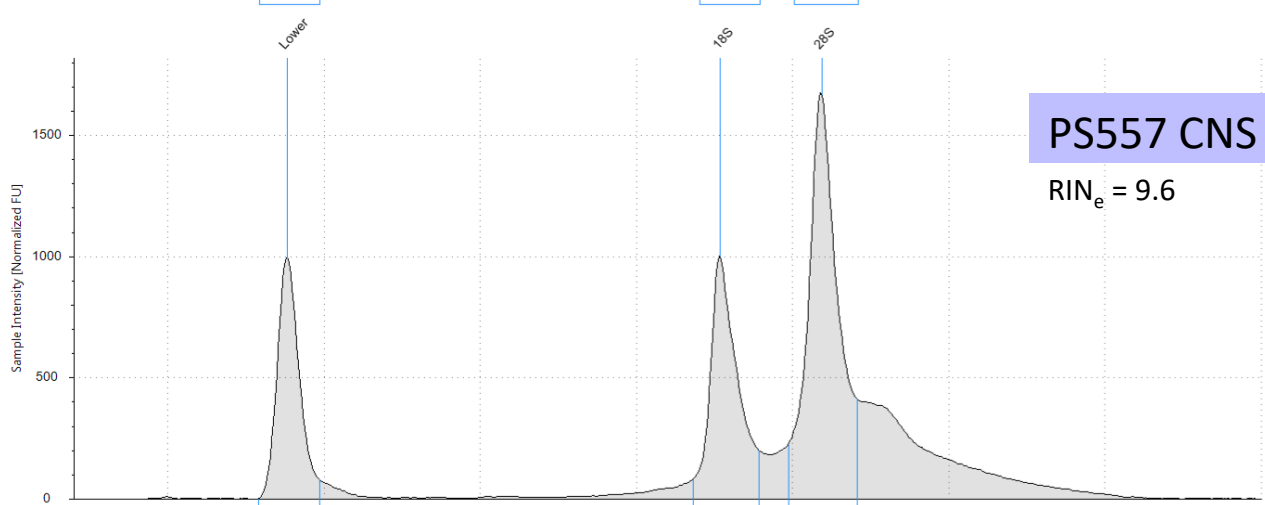

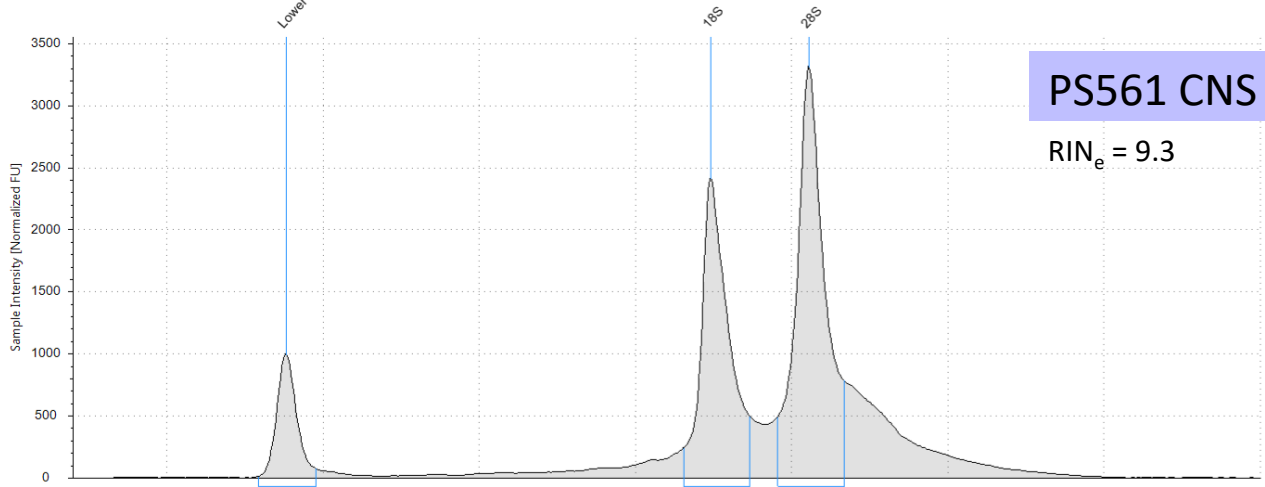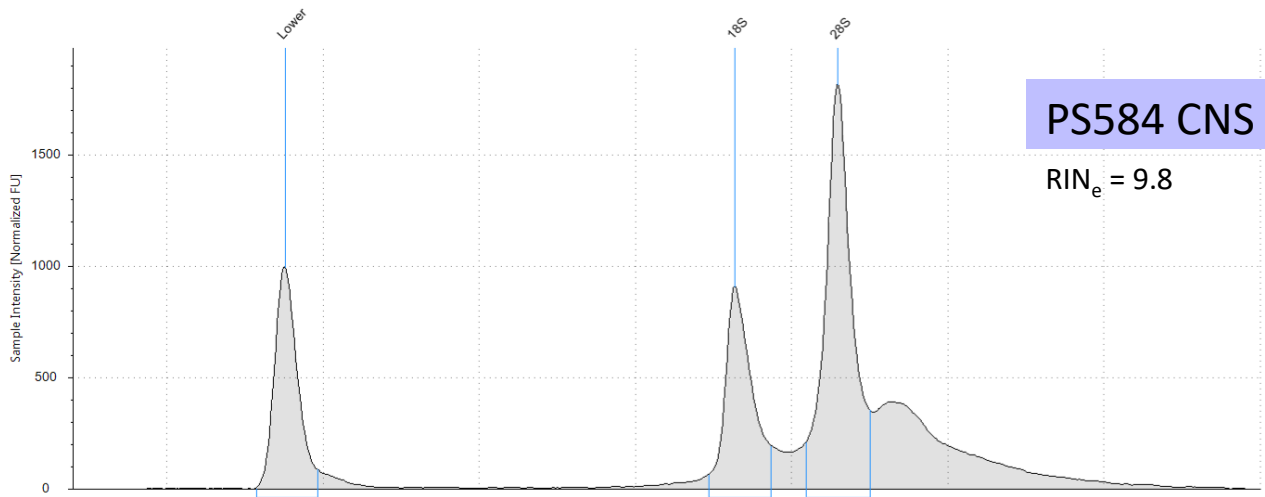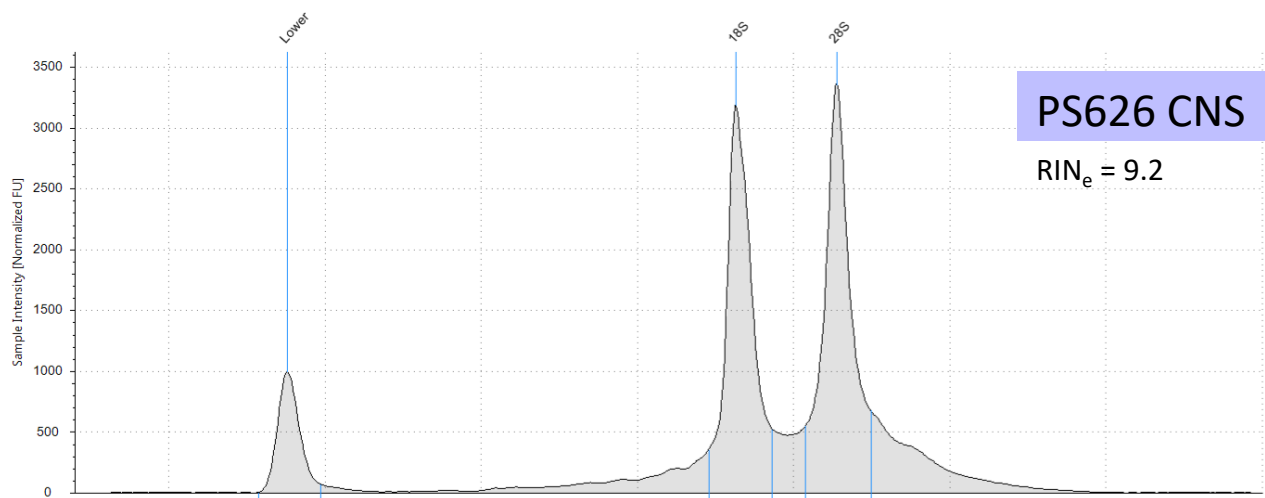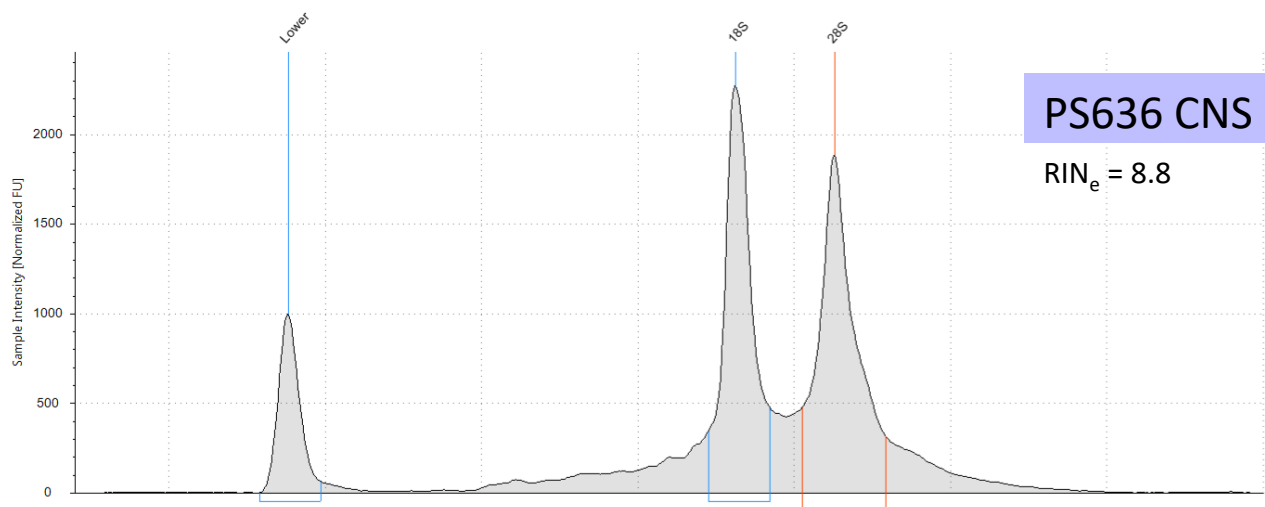

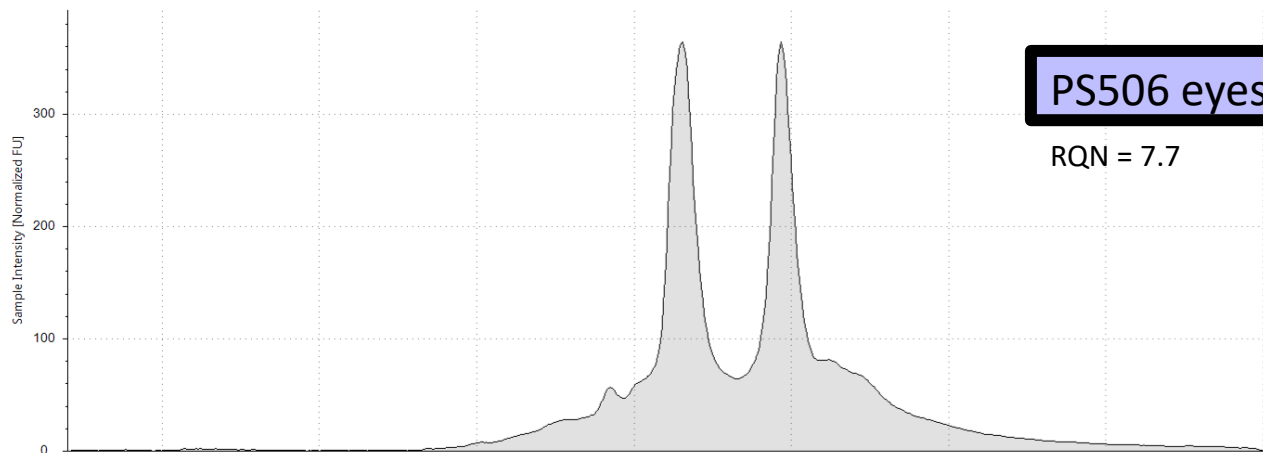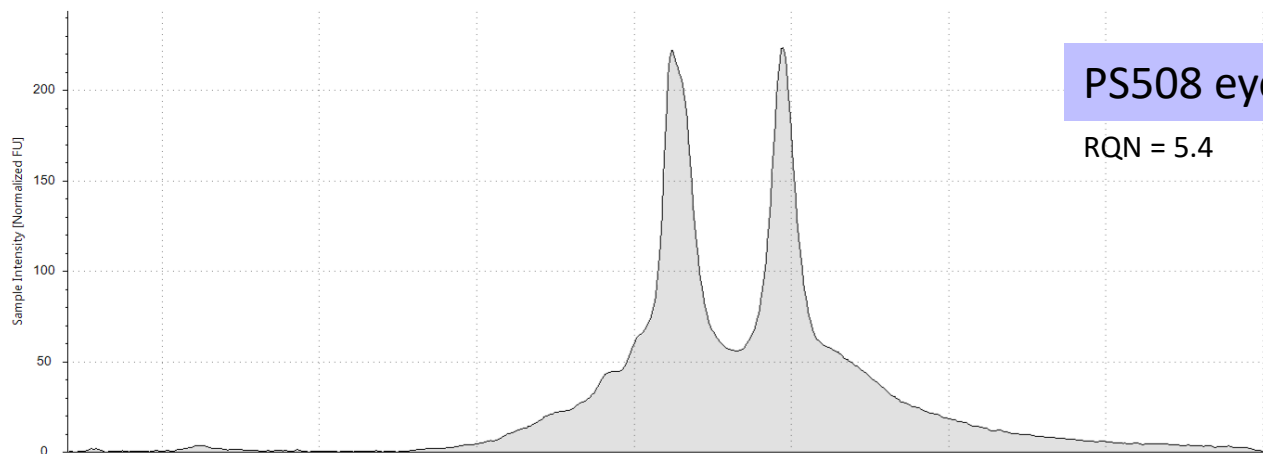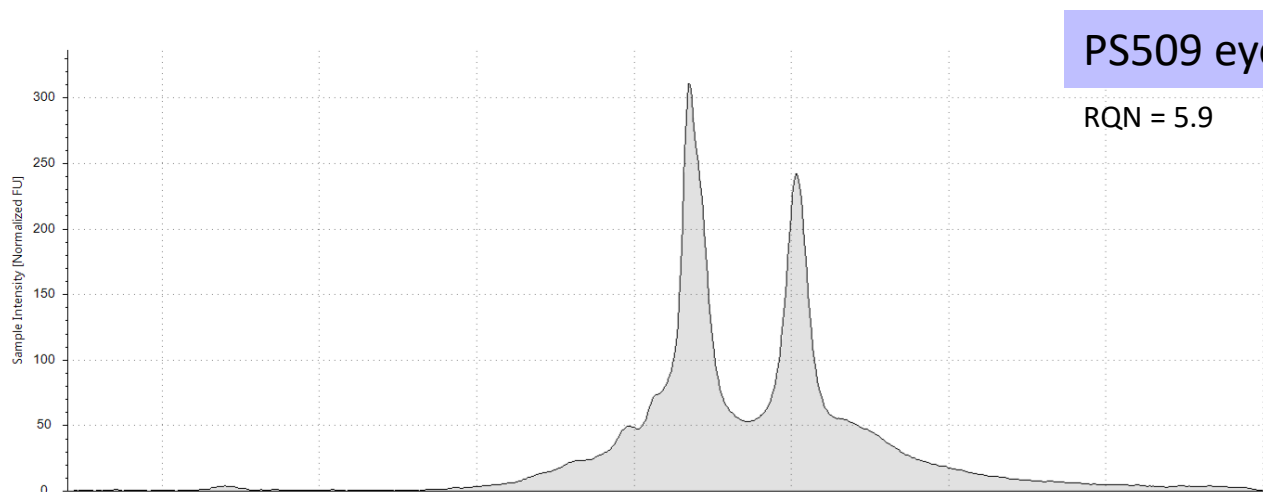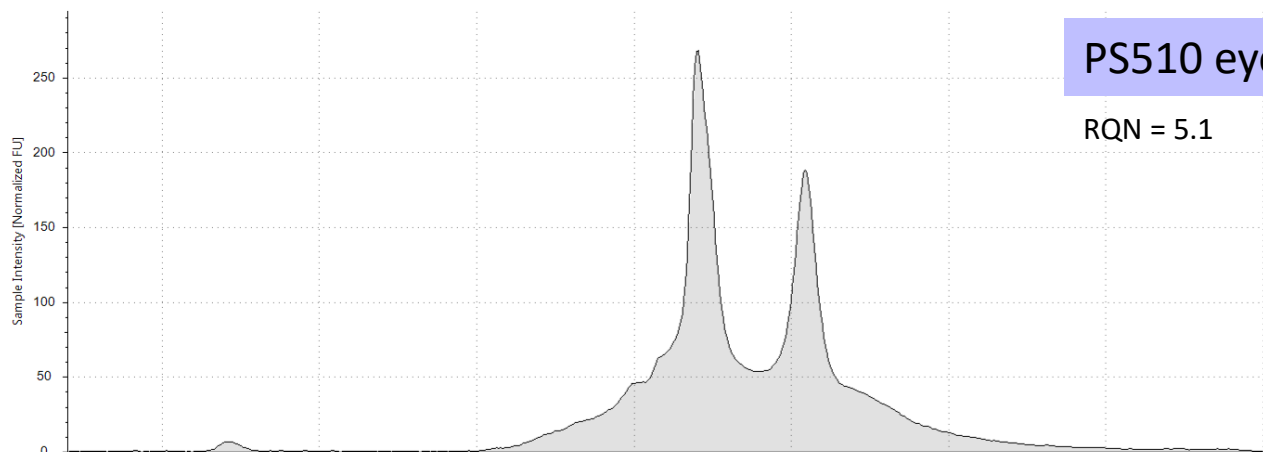

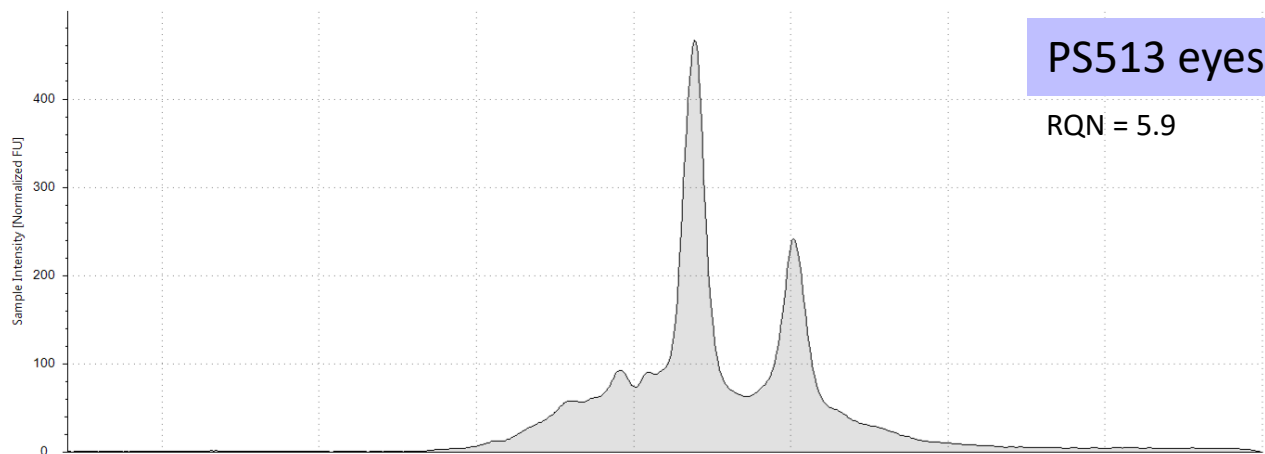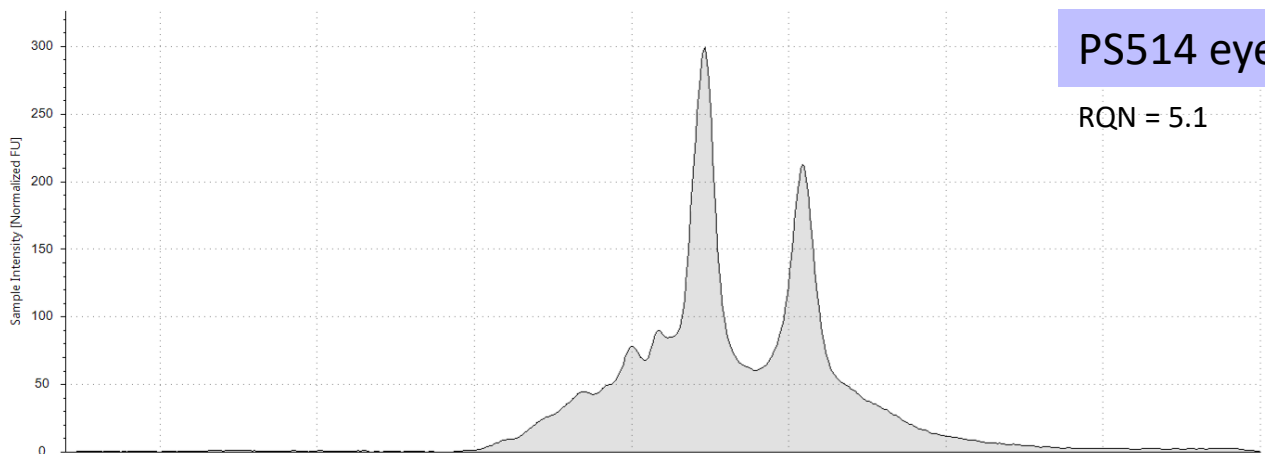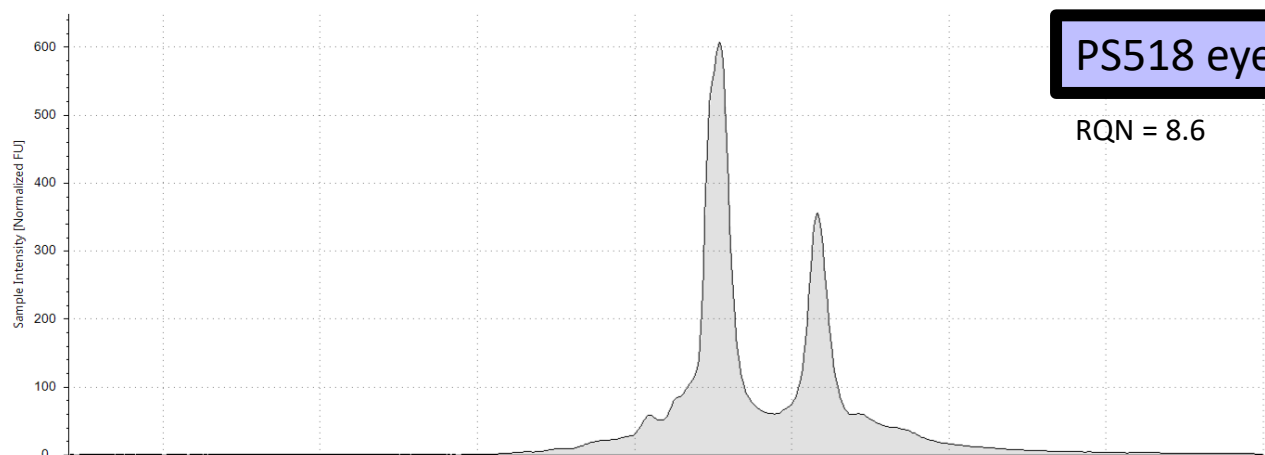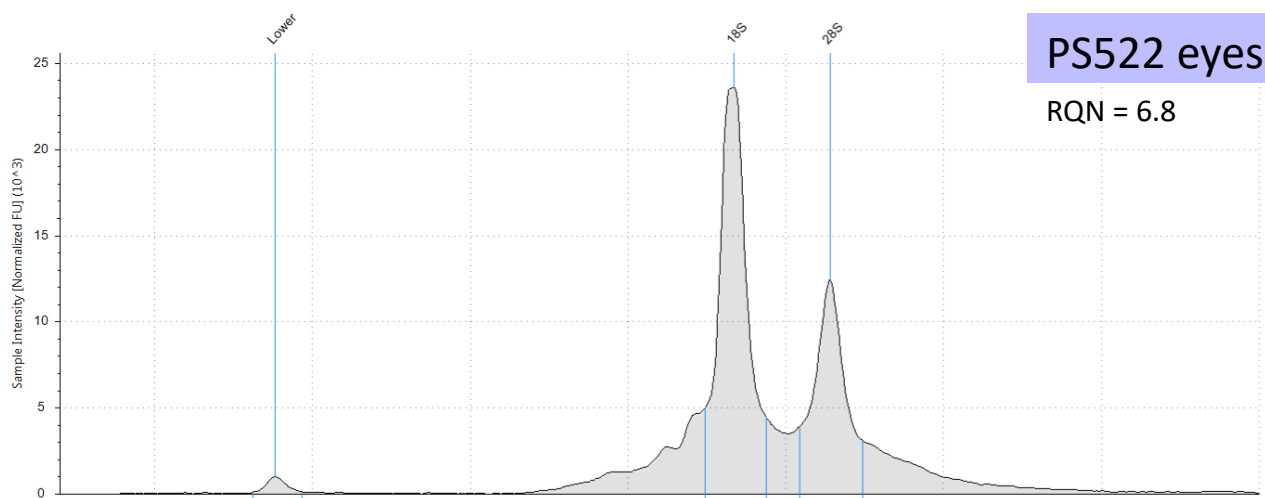

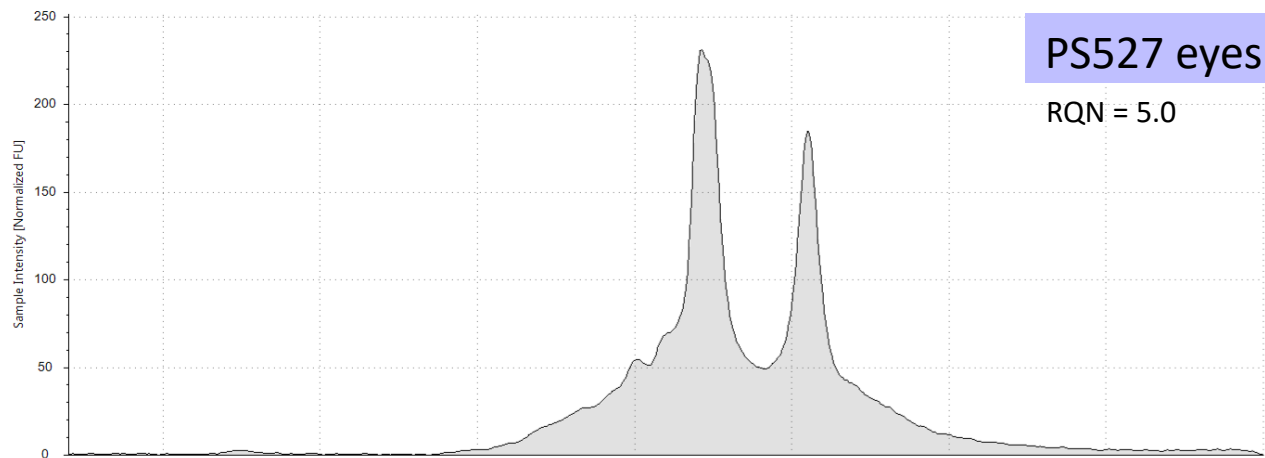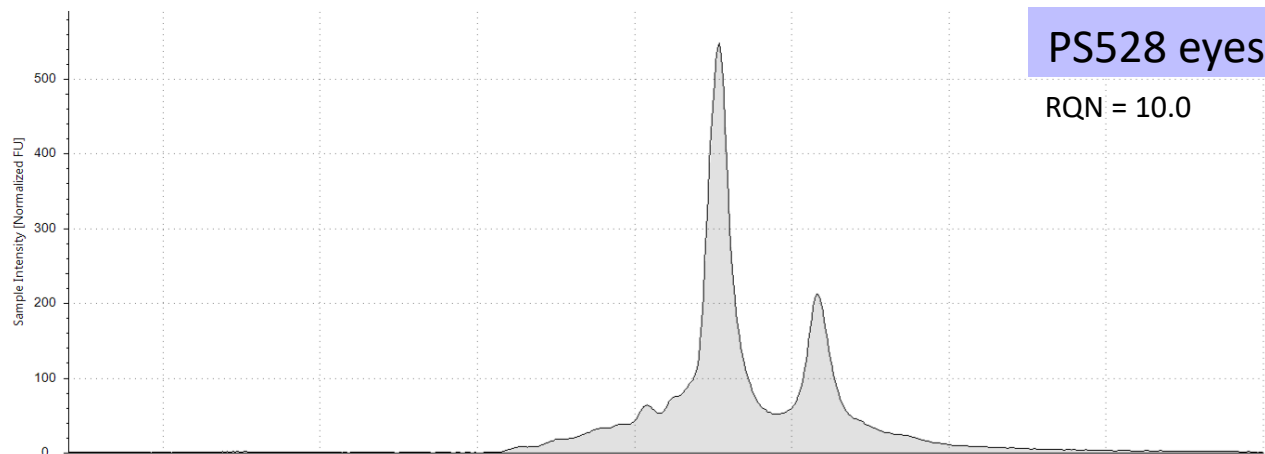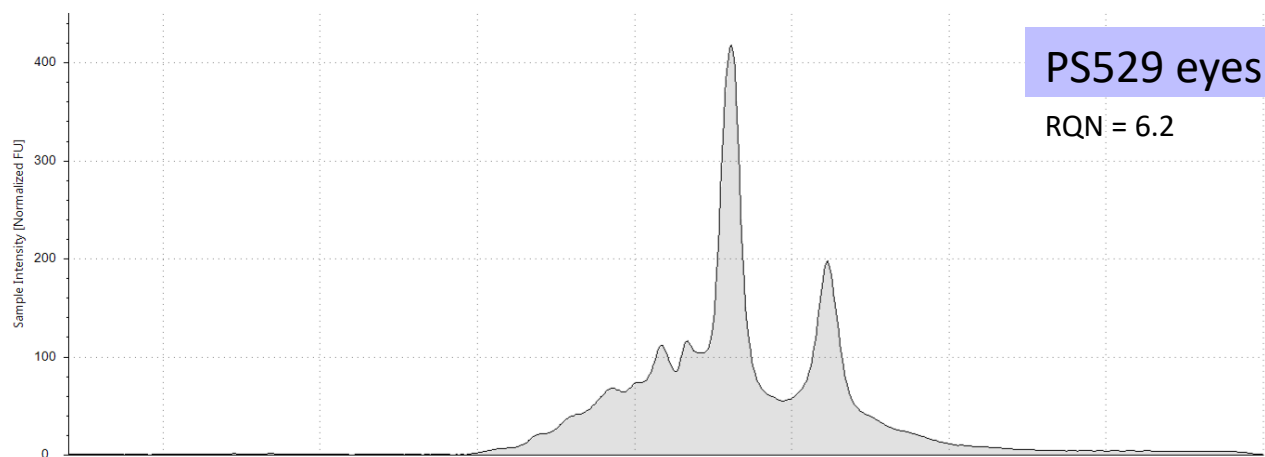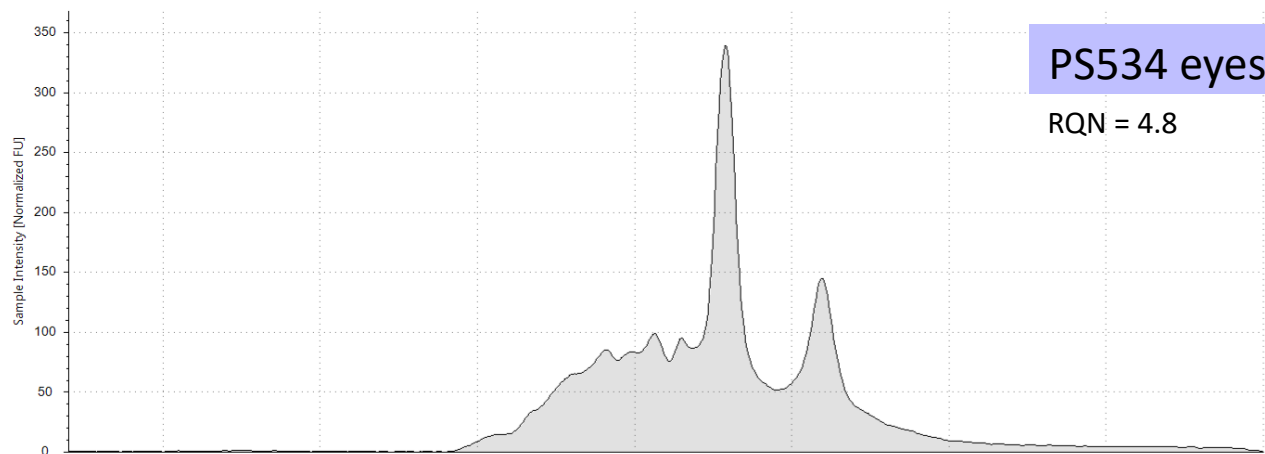

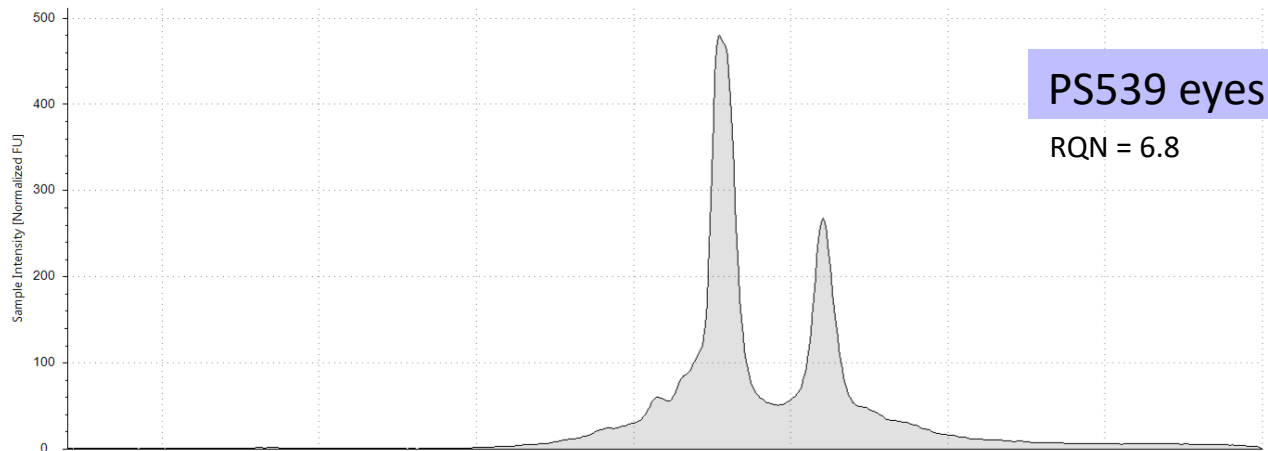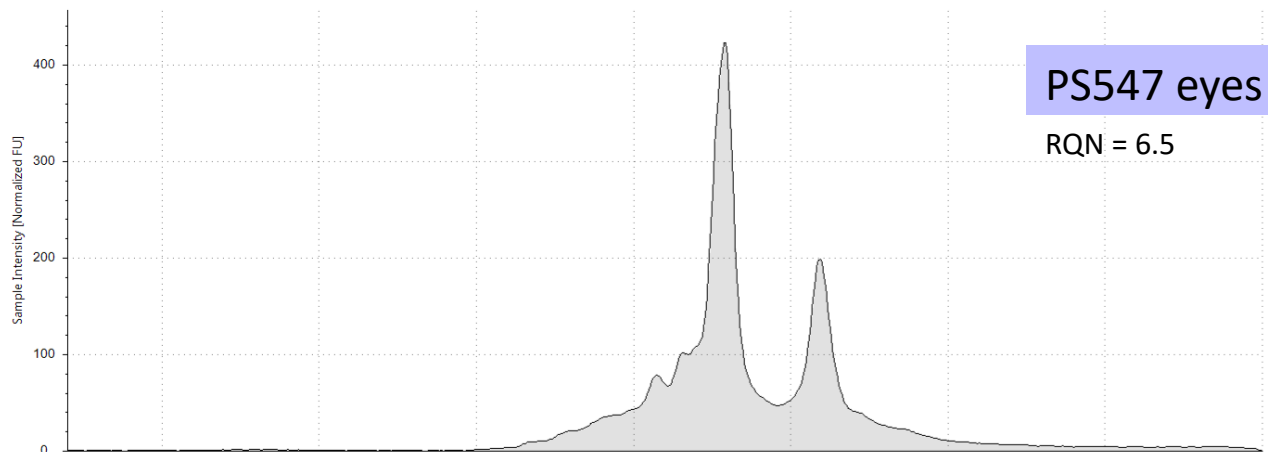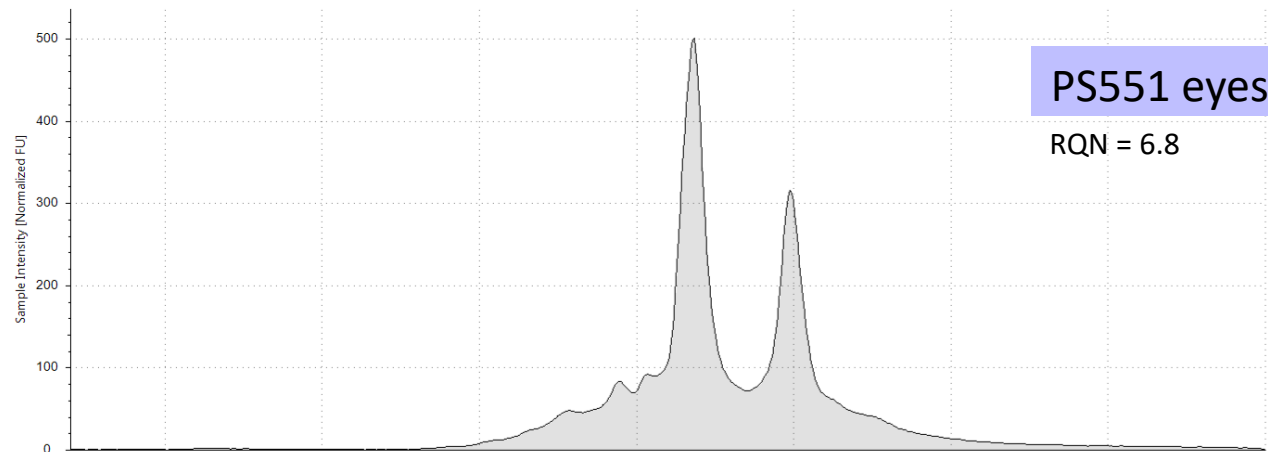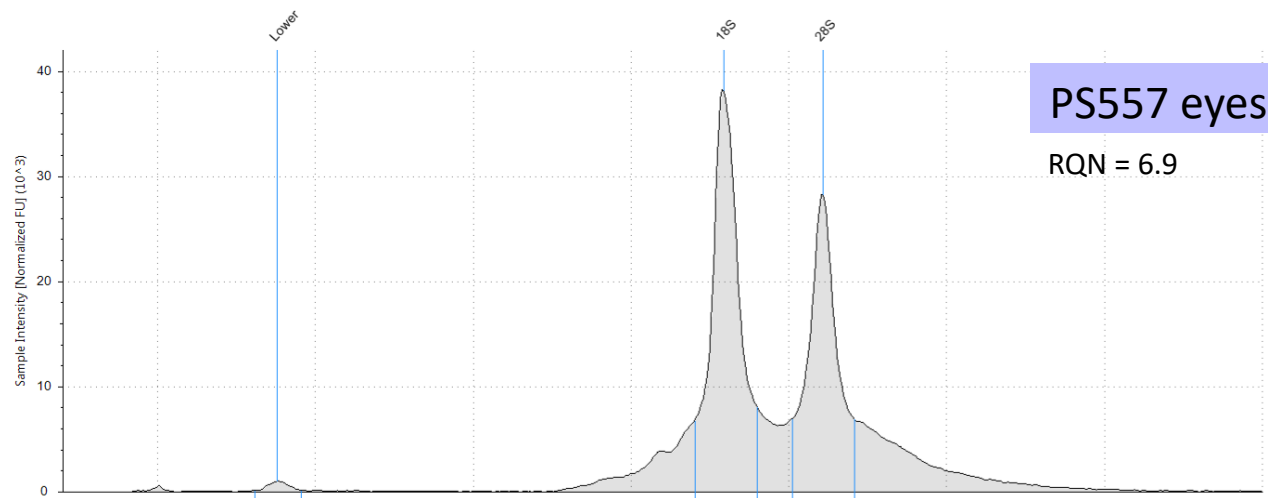

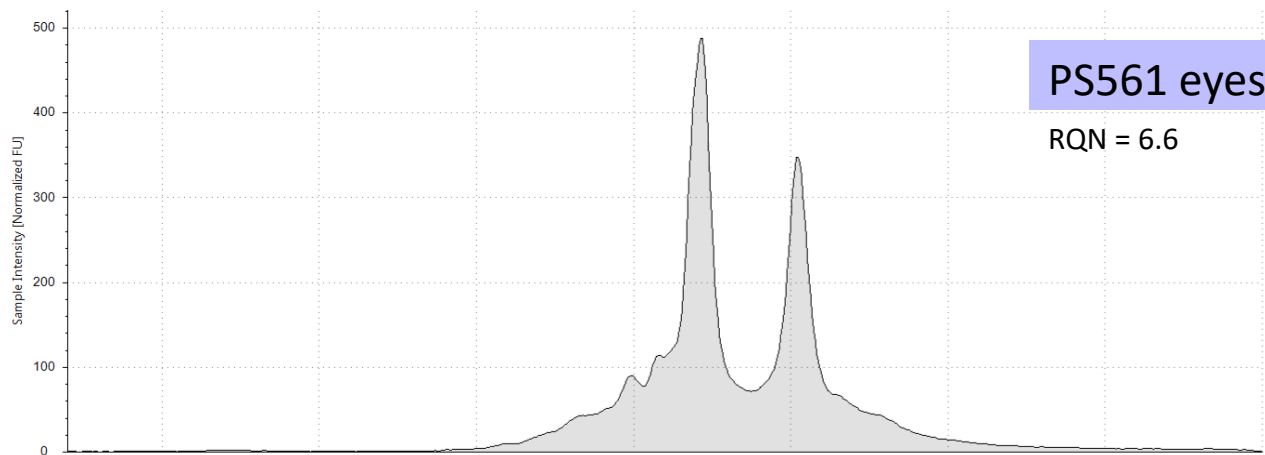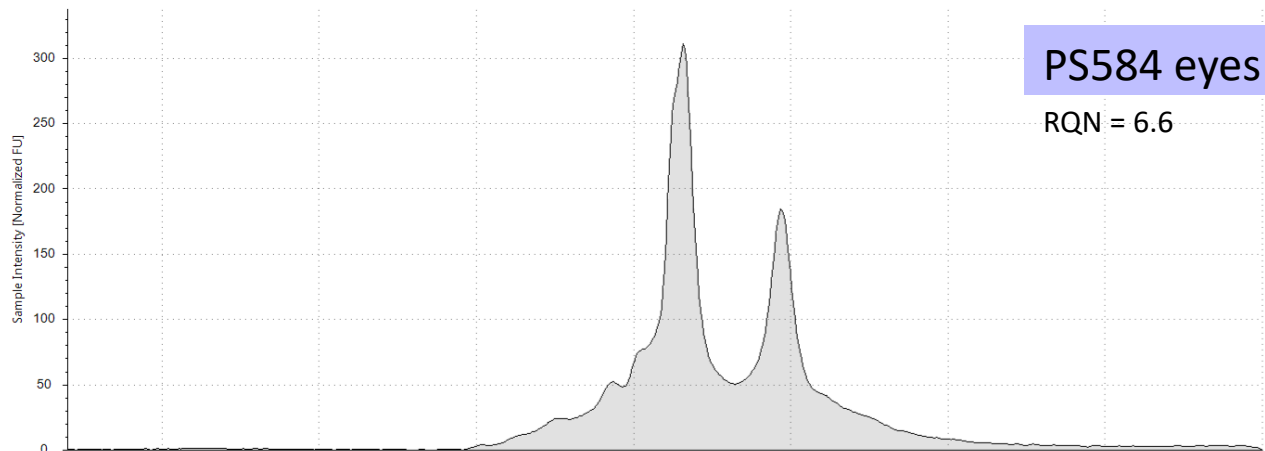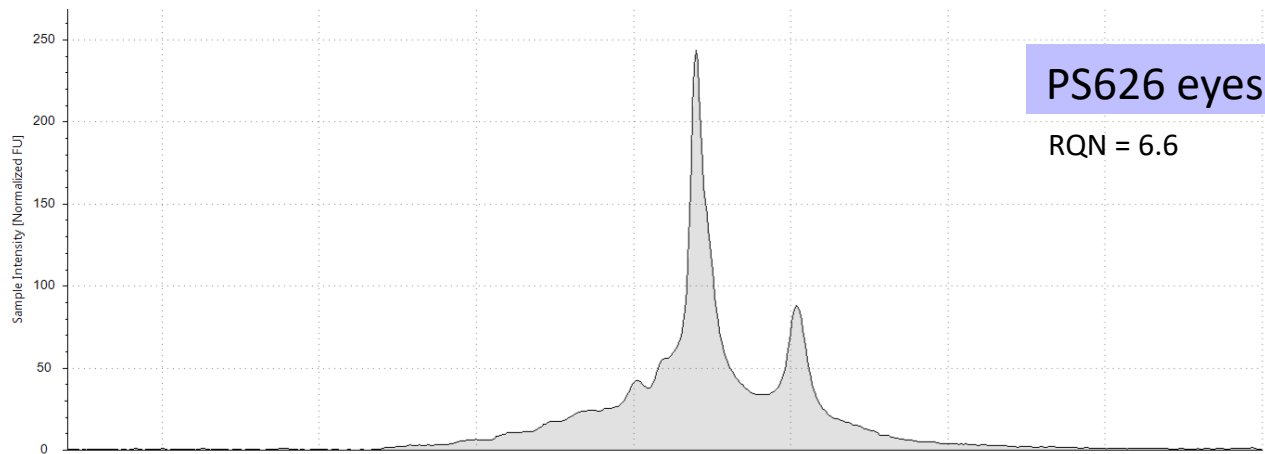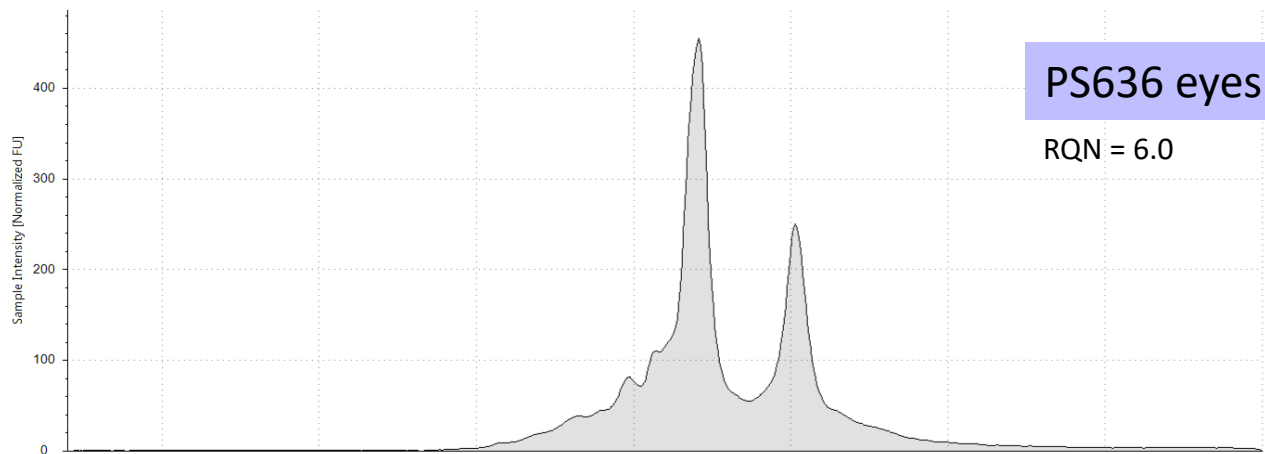

Supplement: Supplementary file 4 — Supplementary Material 4 [file 12864_2024_10542_MOESM4_ESM.pdf]
